# Utility of Circulating Cell-Free DNA in Assessing Microsatellite Instability and Loss of Heterozygosity in Breast Cancer Using Human Identification Approach

**DOI:** 10.3390/genes13040590

**Published:** 2022-03-25

**Authors:** Norah A. Al Sharhan, Safia A. Messaoudi, Saranya R. Babu, AbdulRauf B. Chaudhary, Abdullah A. Alsharm, Abdulmajeed F. Alrefaei, Sultan Kadasah, Muhammad Abu-Elmagd, Mourad Assidi, Abdelbaset Buhmeida, Ángel Carracedo, Wassim Y. Almawi

**Affiliations:** 1 Department of Biopharmaceutical, Laboratories and Research Sector, Saudi Food and Drug Authority, Riyadh 3292, Saudi Arabia; nourahh24@hotmail.com; 2 Department of Forensic Sciences, College of Criminal Justice, Naif Arab University for Security Sciences, Riyadh 14812, Saudi Arabia; smassoudi@nauss.edu.sa (S.A.M.); sramesh@nauss.edu.sa (S.R.B.); achaudhary@nauss.edu.sa (A.B.C.); 3 Surgery Department, King Fahad Medical City, Riyadh 12231, Saudi Arabia; 4 Medical Oncology Department, King Fahad Medical City, Riyadh 12231, Saudi Arabia; aalsharm@kfmc.med.sa; 5 Department of Biology, Umm Al-Qura University, Makkah 24382, Saudi Arabia; afrafaei@uqu.edu.sa; 6 Department of Biology, Faculty of Sciences, University of Bisha, Bisha 61922, Saudi Arabia; sultan.f.kadasah@gmail.com; 7 Center of Excellence in Genomic Medicine Research (CEGMR), King Abdulaziz University, Jeddah 21589, Saudi Arabia; mabuelmagd@kau.edu.sa (M.A.-E.); mourad.assidi@gmail.com (M.A.); abuhme@utu.fi (A.B.); 8 Medical Laboratory Department, Faculty of Applied Medical Sciences, King Abdulaziz University, Jeddah 21589, Saudi Arabia; 9 Grupo de Medicina Xenómica, Fundación Instituto de Investigación Sanitaria de Santiago de Compostela (FIDIS), Center for Research in Molecular Medicine and Chronic Diseases (CiMUS), CIBERER, Universidade de Santiago de Compostela, 15782 Santiago de Compostela, Spain; angel.carracedo@usc.es; 10 Faculté des Sciences de Tunis, Université de Tunis—El Manar, Tunis 1068, Tunisia

**Keywords:** breast cancer, cell-free DNA, diagnosis, liquid biopsy, loss of heterozygosity, short terminal repeats

## Abstract

The diagnostic and prognostic utility of circulating cell-free DNA (cfDNA) in breast cancer (BC) patients was recently reported. Here, we investigated the use of cfDNA to examine microsatellite instability (MSI) and loss of heterozygosity (LOH) for early BC diagnosis. cfDNA and genomic DNA from 41 female BC patients and 40 healthy controls were quantified using NanoDrop spectrophotometry and real-time PCR. The stability of genomic and cfDNA was assessed using a high-resolution AmpFlSTR MiniFiler human identification kit. Significant increases in cfDNA plasma concentrations were observed in BC patients compared to controls. The genotype distribution of the eight autosomal short tandem repeat (STR) loci *D7S820*, *D13S317*, *D21S11*, *D2S1338*, *D18S51*, *D16S539*, *FGA*, and *CSF1PO* were in Hardy–Weinberg equilibrium. Significant differences in the allele frequencies of *D7S820* allele-8, *D21S11* allele-29, allele-30.2, allele-32.2, and *CSF1PO* allele-11 were seen between BC patients and controls. LOH and MSI were detected in 36.6% of the cfDNA of patients compared to genomic DNA. This study highlights the utility of plasma-derived cfDNA for earlier, less invasive, and cost-effective cancer diagnosis and molecular stratification. It also highlights the potential value of cfDNA in molecular profiling and biomarkers discovery in precision and forensic medicine.

## 1. Introduction

Breast cancer (BC) is the most common cancer in women, and is a leading cause of cancer-related deaths worldwide [1], including Saudi Arabia, with 18.7% of all cancer mortality in 2014, and 3629 new BC cases in 2018 reportedly affecting 14.8% of registered Saudi citizens [2]. BC is a complex, multifactorial disease, and is influenced by genetic and environmental factors including gender, age, hormones, obesity, BC family history, breastfeeding, and lifestyle [3,4]. As current BC screening focuses on detecting the associated genetic factors, including *ATM* (Ataxia Telangiectasia Mutated), *CHEK2* (*checkpoint kinase 2*), Breast Cancer 1 (*BRCA1*), Breast Cancer 2 (*BRCA2*), and *PALB2* (partner and localizer of BRCA2) [5], this necessitated the need for the discovery of BC biomarkers with sufficient diagnostic and prognostic sensitivity and specificity.

Microsatellite instability (MSI) and loss of heterozygosity (LOH) are genomic instabilities reported in BC and proliferative breast disease (PBD) [6]. MSI is characterized by nucleotide gain or loss from short tandem repeat (STR) tracts [7], and manifests as novel alleles of varying length [8] due to a lack of DNA mismatch repair [9,10]. On the other hand, LOH involves one allele mutation, followed by the deletion of the remaining alleles [11], partly due to chromosomal deletion, mitotic recombination (MR), gene conversion, point mutations, or intragenic allelic inactivation [12]. The demonstration of MSI events in primary BC samples, and LOH events in stage II and III cancers, indicates that MSI occurs at early stages of carcinogenesis, in contrast to LOH, which occurs at later stages [13,14]. Structurally, LOHs form as a consequence of defective DNA damage as a repair mechanism of double-strand breaks involving interhomolog recombination or gene conversion. This, in turn, leads to the mechanism for generating the gene mutations required for carcinogenesis and the progression of cancer cells [14,15].

First described by Mandel in 1948, cell-free DNA (cfDNA) is extracellular nucleic acid sequences found in body fluids, especially serum and plasma. The properties of cfDNA, including fragmentation profiles, sequence composition, epigenetic modifications, and others, are of significance in health and disease states [16]. For example, the cfDNA sera of BC patients were higher than those of healthy individuals [17], which correlated with cancer stage and response to treatment [17], thus highlighting the role of cfDNA as an alternative (non-invasive) circulating diagnosis biomarker. Although its exact origin has not been fully elucidated, elevated cfDNA levels in cancer patients were attributed to the induction of necrosis, apoptosis, and/or spontaneous active release [18]. The diagnostic utility of cfDNA was studied in many disorders, including myocardial infarction [19], sepsis [20], trauma [21], and liver fibrosis [22], and was also useful in the diagnosis and/or prognosis of cancers, such as ovarian [23], colon [24], prostate [25], lung [26], and breast [27] cancer.

The utility of cfDNA as diagnostic marker in BC and other cancers requires elevated blood concentrations, and cfDNA was shown to harbor tumor-specific DNA mutations for early disease detection [28,29,30]. Genetic/epigenetic alterations, including point mutations, LOH, microsatellite alterations, and methylation [31], are predictive of the metastatic burden in BC patients [32]. Furthermore, as cfDNA levels are higher in *BRCA1* and *BRCA2* mutation carriers compared to non-carriers [33], cfDNA levels were demonstrated to be associated with tumor size and staging [34,35], prompting the speculation that cfDNA is useful in assessing the response to therapy, and is predictive of disease recurrence [33].

An increasing interest in the importance of cfDNA in forensic medicine was evidenced from the analysis of touched surfaces [36], and was demonstrated in many samples, such as blood and saliva. This highlighted the potential to increase the DNA yield in forensic casework samples in general, and in contact traces in particular [37]. It is evidence that cfDNA deposited by handling provides genetic information, evidenced by the average yield of 11.5 ng of DNA recovered from 1 mL cell-free sweat samples. This supports the notion that suitable length cfDNA for standard DNA profiling is transferred during handling or touching items [36].

While there are no universal or standard guidelines examining distinct cfDNA for different applications, the workflow of their assessments including critical steps (sample collection, storage, transportation, extraction, laboratory and bioinformatics analyses, statistical evaluation) can potentially influence the outcomes and informational value of the performed analysis [16]. This study evaluated the utility of cfDNA levels as diagnostic markers for BC, and to investigate the MSI and LOH of eight autosomal STR markers in cfDNA isolated from BC patients and healthy controls. This study also highlights the utility of plasma cfDNA STR profiling for forensic purposes in other settings.

## 2. Materials and Methods

### 2.1. Study Subjects

This was a retrospective case-control study conducted in the Department of Forensic Biology at the College of Forensic Sciences, Naïf Arab University for Security Sciences (Riyadh, Saudi Arabia). The recruitment of the 41 BC patients and 40 age- and ethnicity-matched healthy control women was conducted at King Fahad Medical City (KFMC) between December 2015 and March 2016. The inclusion criteria included histologically confirmed invasive BC and subjects who did not receive chemotherapy, radiotherapy, or hormone therapy, while the exclusion criteria included a history of other cancers. The controls consisted of cancer-free, healthy women with no personal or family history of any cancer. The Research Ethics Committee of King Fahad Medical City approved the research protocol (IRB approval number: FWA00018774), and the participants were required to sign an informed consent form before participating in the study.

### 2.2. Blood Collection and DNA Extraction

Peripheral blood was collected from participants in 2 mL EDTA-containing tubes, and plasma and buffy coat fractions were isolated within 6 h of collection. The plasma samples were stored at −20 °C pending analysis. cfDNA isolation and genomic DNA extraction from peripheral blood leukocytes (as internal control) were isolated using a QIAamp^®^ DNA Mini Kit according to the manufacturer’s instructions (Qiagen, Hilden, Germany), and stored at −20 °C until processing. The extracted DNA was quantified by NanoDrop™ and real-time PCR using a Quantifiler^®^ Duo DNA Quantification Kit according to the manufacturer’s instructions (Thermo Fisher, Riyadh, Saudi Arabia).

### 2.3. Amplification of STR Markers

The STR markers were amplified using an AmpFlSTR^®^ MiniFiler™ PCR Amplification Kit according to the manufacturer’s instructions (Thermo Fisher, Riyadh, Saudi Arabia). This validated human tool with enhanced throughput allows for the simultaneous amplification and separation of *D7S820*, *D13S317*, *D21S11*, *D2S1338*, *D18S51*, *D16S539*, *FGA*, and *CSF1PO* autosomal STR loci, in addition to the sex-determining marker, amelogenin. PCR was carried out in a Gene-Amp^®^ PCR 9700 thermal cycler (Applied Biosystems, Waltham, MA, USA; Thermo Fisher, Riyadh, Saudi Arabia), with initial incubation at 95 °C for 11 min, followed by 30 cycles of denaturation (94 °C, 20 s), annealing (59 °C, 2 min), and extension (72 °C, 1 min). The data were analyzed using 7500 System Sequence Detection Software (SDS) v1.2.3 (Applied Biosystems, Waltham, MA, USA) with a baseline of 3–15 cycles and a threshold of 0.2. The PCR products were analyzed by capillary electrophoresis using an ABI 3130 Genetic Analyzer^®^ according to the manufacturer’s instructions (Applied Biosystems, Waltham, MA, USA). The samples were analyzed using GeneMapper^®^ IDX version 1.1 analysis software (Thermo Fisher, Riyadh, Saudi Arabia). The STR Allele frequencies were calculated using GenAlEx V. 6.503.

### 2.4. Statistical Analysis

Statistical analysis was performed using SPSS (Statistical Package for the Social Sciences) version 22.0 (IBM, Armonk, NY, USA). Qualitative data were expressed as number and percent of the total and compared using a χ^2^ goodness-of-fit test. Continuous variables were expressed as mean ± standard deviation (SD) and were compared with a Student’s *t*-test (two-sided). *p* < 0.05 (two-tailed) was considered statistically significant.

## 3. Results

### 3.1. Clinical and Demographic Characteristics of Study Cohorts

The clinical and demographic characteristics of the 41 BC patients and the 40 cancer-free control subjects are shown in Table 1. No significant differences were found in the mean age at study inclusion (*p* = 0.61) and previous use of oral contraceptives (*p* = 0.91) between BC patients and cancer-free controls. Significant differences between the BC patients and controls were found in BMI (*p* = 0.01), obesity (*p* = 0.015), breastfeeding (*p* = 0.008), and family history of BC (*p* < 0.0001). Accordingly, these were selected as the main covariates that were controlled for in the subsequent analysis.

### 3.2. Quantitative Analysis of cfDNA

The data from Table 2 show that the cfDNA levels, measured by real-time PCR and spectrophotometry, were significantly higher in BC patients (58.3 (0.0–156.0) ng/mL) than the control subjects (20.4 (4.0–48.5) ng/mL) (*p* = 0.0001). A significant difference in cfDNA levels was only seen among BC patients according to their nodal status (64.9 (36.9–156.0) ng/mL vs. 50.7 (0.0–120.0) ng/mL; *p* = 0.04), but not age (*p* = 0.73), tumor size (*p* = 0.09), pathologic stage (*p* = 0.48), estrogen (ER; *p* = 0.72), progesterone (PR; *p* = 0.88), or human epidermal growth factor (HER; *p* = 0.51) receptor status.

### 3.3. Allelic Frequencies of STR Markers

The genotype distribution of the tested STR markers were in Hardy–Weinberg equilibrium (Table 3). The allelic frequencies of *D7S820* allele 8 (OR (95% CI), 0.26 (0.09, 0.75)), *D21S11* alleles 29 (OR (95% CI), 0.12 (0.03, 0.42)), 31.2 (OR (95% CI), 0.23 (0.05, 1.12)), and 32.2 (OR (95% CI), 0.18 (0.04, 0.85)), and *CSF1PO* allele 11 (OR (95% CI), 0.44 (0.22, 0.86)) were significantly lower in BC patients than in healthy controls, thus imparting a BC-protective nature to these alleles. In contrast, the allelic frequencies of *D2S1338* allele 24 (OR (95% CI), 4.22 (0.87, 20.53)), and *D21S11* allele 30.2 (*p* = 0.046) were significantly higher in the BC patients compared to healthy controls, thus imparting a BC-susceptible nature to these alleles. These associations remained significant after controlling for BMI, breastfeeding, and family history of breast cancer.

### 3.4. STR Genotyping and Altered STR Profiles in cfDNA versus WBCs

A panel of eight polymorphic STR markers was profiled in cfDNA/WBCs matched samples and compared between the sample fractions from each individual. The results obtained demonstrated the likelihood of finding unique STR profiles from extracted cfDNA, even from patients at advanced BC stages and identical to the STR profile obtained from the related control samples (Supplementary Tables S1 and S2). Moreover, the STR profile was comparable between the cfDNA and whole blood samples in the control participants. Full informative profiles were obtained from 66% of BC patients’ samples, while partial profiles were obtained for the remaining samples. Total DNA degradation was seen in the whole blood and cfDNA fractions from Pp1, Pp5, and Pp32 patients (Supplementary Tables S1 and S2).

**Figure 1:**
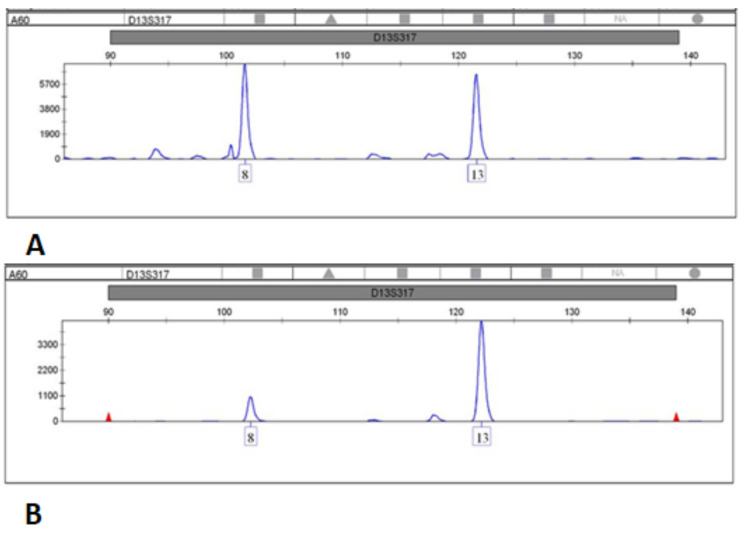
Loss of Heterozygosity observed at locus D13S317. Panels (**A**) represents normal genotypes in genomic DNA, while Panel (**B**) is a representative of MSI in cfDNA.

**Figure 2:**
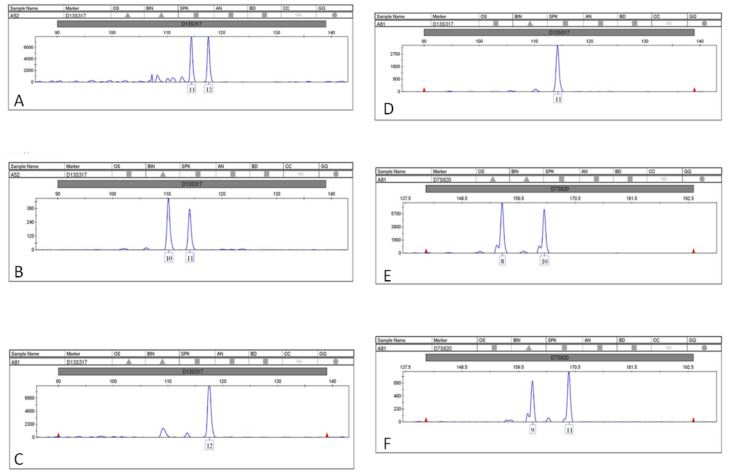
Genomic instability analysis of cfDNA loci. Microsatellite instability observed in two loci (*D13S317*, *D7S820*). Electropherogram (**A**–**D**) indicate the MSI at locus *D13S317*. Electropherogram (**E**,**F**) indicate the MSI at locus *D7S820*.

LOH and MSI were detected in 2 of the 41 (4.88%) blood BC samples (Table 4). This was significantly lower than the LOH and/or MSI detected in 15 cfDNA plasma samples (36.60%). These results further demonstrate that cfDNA is a sensitive and reliable tool for STR analysis. LOH was detected in 31.7% of the samples in at least one locus, and MSI was detected in 6 of the 41 BC samples (14.6%) (Figure 1 and Figure 2). Both LOH and MSI were observed in cfDNA, and only one patient had LOH in more than one locus. On the other hand, MSI were observed in four patients, two of whom had MSI at more than one locus. Furthermore, patient 16 had four altered microsatellites, the highest rate of such alterations, and four patients displayed both LOH and MSI events.

**Figure 3:**
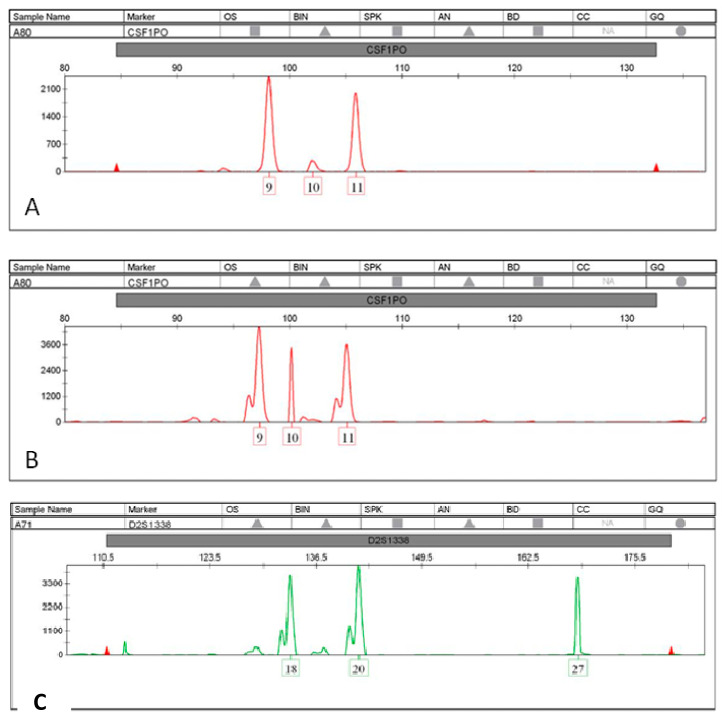
Extra alleles observed at locus CSF1P (**A**,**B**), and at locus D2S1338 (**C**).

We also investigated the features associated with unstable loci (Table 4). After stratification according to repeat composition and STR alterations, the compound microsatellites were found to be preferentially unstable compared to other repeat types. *CSF1P0* and *FGA* (altered in patients 10, 13, 15, and 20) were the most susceptible STR loci, followed by *D13S317* and *D2S1338* (altered in patients 8, 11, and 16). In addition, LOH alteration was detected in the *FGA* locus. The insertion of an extra allele was detected in patients 11, 15, and 20 (Figure 3), while the same alleles related to genomic DNA were observed in cfDNA for patient 15. A deletion of one allele was also detected in patients 11 and 20. No correlations were found between LOH and MSI and the grade or stage of tumor. All electropherogram plots can be seen in Supplementary Figure S1.

## 4. Discussion

Cancer is driven by the hyperactivity of cancer-promoting oncogenes and/or the inactivation of tumor suppressor genes [38]. While clinicopathological and clinical variables are helpful in predicting cancer outcomes, the characterization of solid cancers relies on invasive biopsies and/or open surgical sampling. The evaluation of novel biomarkers as alternative diagnostic tools in cancer has been undertaken with varying levels of success. This “liquid biopsy” is based on the findings that less-invasive biological material (whole cells, nucleic acids, and microvesicles) from primary tumors and/or metastatic lesions is excreted in body fluids [39,40]. Previous study evaluating the potential use of cfDNA for medical/forensic purposes showed inconsistent results on whether DNA profiles from cfDNA-concentrated supernatant in different types of samples contain “floating” information not detected by only analyzing the cell pellet. The study suggested that the supernatant phase should be stored for potential additional analysis in case the cell pellet does not result in a useful DNA profile [37,41]. Given the complexity, heterogeneity, and comorbidities of advanced cancer, noninvasive cfDNA analysis is effective and inexpensive. The current study addressed the utility of such a liquid biopsy for studying BC and its capability to provide a useful full/partial STR profile that can be used in precision oncology.

BC patients were characterized based on tumor size, location, stage, histological classification, and the presence of conventional markers, including ER, PR, and HER2, as well as general risk factors (age, BMI, oral contraceptive use, and breastfeeding). While patients were age-matched to controls, BMI was significantly higher in BC patients, consistent with previous reports documenting BMI as a significant risk factor for BC [42,43]. This was also supported by the findings that a chronic low level of inflammation is associated with obesity, and contributes to BC by damaging the DNA [44]. Moreover, cell proliferation in BC is due to the production of excessive amounts of estrogen and adipokines from fat cells [43,45,46].

Plasma-derived cfDNA was shown to have diagnostic and prognostic potential for ovarian [23], colorectal [24], prostate [25], lung [26], and breast [27] cancers and could replace and/or complement tests based on tissue biopsies [47]. cfDNA was reported to be valuable in monitoring the progression of prostate cancer [48], chemotherapy outcomes in colorectal cancer [49], and as a predictor of survival in ovarian cancer [50]. Compared to WBC, our results showed that STR profiling using cfDNA has identified high MSI and LOH, consistent with previous study showing that cfDNA-based profiling is useful in lung cancer, and that the microsatellite analysis of plasma DNA is a novel tool for tumor staging, management, and detection [51]. It is noteworthy that cfDNA analysis has not reached the level of required validity needed for wider application in clinical diagnostics, mostly due to preanalytical (biological, environmental, technical), analytical variability, and postanalytical variability, with error margins ranging from 10 to 60% [52]. This highlights the need for standardizing preanalytical conditions.

Plasma cfDNA was significantly higher in the BC patients than the healthy controls, in agreement with earlier studies that reported elevated cfDNA levels in the serum/plasma of cancer patients, particularly in metastatic more than non-metastatic cases [41,53,54]. No correlation was found between serum DNA concentrations and primary tumor size or location [17,55], similar to what was reported for lung cancer, where circulating plasma DNA levels were 85-fold higher than in healthy individuals [56]. Approximately 58% of newly diagnosed prostate cancer cases, 49% of BC patients, and 27% of prostate cancer patients on therapy have elevated DNA levels compared to the control group [57].

In the present study, plasma cfDNA was further characterized by studying STR markers’ gene variants using the AmpFlSTR^®^ MiniFiler PCR Amplification Kit, which increases the likelihood of obtaining a complete STR profile from a degraded sample by bringing the primers closer to the locus repeat regions, thus allowing the generation of smaller amplicons [58]. New STR locus alleles created by insertion or deletion were also identified, and their association with BC was subsequently confirmed. While not tested here, STR mutations in the coding regions, introns, or untranslated regions reportedly affect gene expression or protein function by modulating transcription factor binding, spacing between promoter elements, enhancers, cytosine methylation, and alternative splicing [59]. MSI and LOH were observed in BC patients, affecting all STR markers analyzed in this study.

MSI and LOH are aberrations associated with early steps in tumorigenesis [15,16], and their detection in cfDNA underscores their utility in screening BC patients when using liquid biopsy. In this study, the consecutive accumulation of detected MSI and LOH in multiple cfDNA loci were linked with the deregulation of tumor suppressor genes often found to be inactivated in early precancerous and cancerous cells [52,53,54], hence precipitating secondary malignancies and/or resistant cancer phenotypes. In support of this notion was our finding that MSI- or LOH-associated STR genetic instability was reported in cancer patients, potentially serving as an early prognostic and diagnostic factor in BC. The presence of an extra allele of a different size was seen in 8% of the tumor DNA samples, but not in the normal DNA of the same patient [60]. STR instability was observed in 37% of the BC samples, similar to a previous study in which 42% of the patients had LOH in at least one marker [61]. Unstable cfDNA loci in cancer-associated genes were consistently detected in several studies. For example, *FGA*, located in the 4q28 locus, was only subject to LOH due to mammalian-wide interspersed repeat (MIR) repetitive sequences [62], with an unusual T4G motif possibly responsible for the recurrent deletion [63]. Alterations in the *FGA* locus have been detected in invasive ductal carcinomas, highlighting the importance of this chromosomal region [64].

LOH in chromosome 16q was reported in BC. The inactivation of an unknown tumor-suppressor gene on 16q24.2-qter, which includes the D16S539 locus, was involved in the initiation of sporadic BC, regardless of tumor stage and grade [65]. Moreover, LOH on chromosome 13 loci was shown to play a role in carcinogenesis [66,67,68]. The D13S317-region harboring 13q22-31 exhibited higher LOH (69%) in BRCA1-associated adnexal carcinomas, thus harboring putative tumor suppressor genes involved in the carcinogenesis of this hereditary cancer [69].

cfDNA in cancer patients contains both tumor and non-tumor DNA, confirming the previous findings that tumor cells, mostly from the tumor microenvironment, are the main source of cfDNA release [70]. Several studies documented that neoplastic cfDNA alterations, such as MSI, LOH, or mutations, contribute to oncogenesis, and can be detected in the tissues and blood of cancer patients [71,72]. In our study, cfDNA-specific STR profiles were more informative than WBC-extracted DNA STR profiles, an indication that cfDNA-instability STR analysis is a powerful tool to assess cfDNA origin (tumor cells vs. microenvironment). No association was found between MSI or LOH and cancer stage, in contrast to previous reports [13,14], likely due to the small size of the cohort studied.

STR marker microsatellite instability, caused by mutations in the mismatch repair system (MMR) genes, occurs in cancer because of the accumulation of mutations during carcinogenesis [9]. The inactivation of tumor suppressor genes by intragenic mutation in one allele and the subsequent loss of the corresponding (wild) allele lead to LOH [73], while the association between cancer and the LOH of a specific STR suggests a likely cause–effect relationship.

However, this study could have some limitations related to the (inherent) bias of a case-control study and reverse causality and possible variation of cfDNA levels among patients due to different stages and/or treatments, as well as a the relatively small sample size of the cohort. Therefore, further studies with a larger sample size at different cancer stages, followed by validation using cancer tissue biopsies, are recommended.

## 5. Conclusions

This is the first study in Saudi Arabia to highlight the promising use of STR and LOH as potential targets for the discovery of cancer biomarkers, particularly in BC diagnosis. This study reported interesting STR and LOH markers using blood liquid biopsy-driven cfDNA analysis in BC patients. Our results confirm that the cfDNA levels are elevated in the peripheral blood. Notably, the identified genetic alterations in the cfDNA samples were also found in BC tissues or WBCs. These results also highlight the potential value of the biomarker discovery approach in both human identification studies and forensic cases. This argues for the utility of this approach as a non- or less invasive application in molecular profiling and biomarker discovery, either in precision or forensic medicine.

## Figures and Tables

**Table 1:** Demographics and clinical characteristics of study subjects.

Characteristics	Healthy Controls (*n* = 40)	BC Patients (*n* = 41)	*p*-Value ^1^
Age (years) ^2^	49.1 ± 11.0	48.80 ± 8.28	0.61
BMI (kg/m^2^) ^2^	27.6 ± 5.66	32.89 ± 7.96	0.01
Obesity ^3^	21 (52.50)	32 (78.05)	0.015
Use of oral contraceptives ^3^	23 (57.50)	19 (46.34)	0.91
Breastfeeding ^3^	20 (50.00)	9 (21.95)	0.008
Family history ^3^	41 (100.00)	25 (59.51)	<0.0001

^1^ Student’s *t*-test (2-tailed) for continuous variables, Pearson’s χ^2^ test for categorical variables. ^2^ Mean ± SD. ^3^ Number of subjects (percent total).

**Table 2:** Cell-free DNA in breast cancer patients and control subjects ^1^.

Parameters	Number (% Total)	Cell Free DNA ^1^	*p*-Value ^2^
Status (Cases:Controls)	41:40	58.3 (0.0–156.0):20.4 (4.0–48.5)	<0.0001
Age (<50 year:≥50 year)	25 (61):16 (36)	58.7 (0.0–96.7):57.6 (13.3–156.0)	0.73
Nodal status (−ve:+ve)	14 (34.1):26 (63.4)	64.9 (36.9–156.0):50.7 (0.0–120.0)	0.04
Size (<2 cm:≥2 cm)	6 (17.1):35 (85.4)	11.50 (21.8–76.6):61.13 (0.0–156.0)	0.09
Stage (I:II:III:IV)	6 (14.6):13 (31.7):16 (39.0):5 (12.2)	54.5 (36.9–68.0):62.0 (13.3–156.0):44.2 (0.0–99.1):49.9 (33.8–120.0)	0.48
ER status (+ve:−ve)	29 (68.3):11 (29.3)	58.5 (0.0–156.0):62.0 (13.3–120.0)	0.72
HER2 (+ve:−ve)	23 (56.1):15 (36.6)	61.4 (0.0–156.0):58 (13.3–120.0)	0.51
PR status (+ve:−ve)	26 (63.4):13 (31.7)	58.3 (0.0–156.0):60.8 (13.3–120.0)	0.88

HER: human epidermal growth factor receptor; ER: estrogen receptor; PR: progesterone receptor. ^1^ Plasma cfDNA in ng/mL (range). ^2^ 2-tailed Student’s *t*-test (continuous variables), Pearson’s χ^2^ test (categorical variables), and Kruskal-Wallis test for non-parametric independent samples.

**Table 3:** STR allelic frequency in cases and control subjects.

STR	Alleles	Controls	Cases	χ^2^	*p* ^1^	OR (95% CI)
*D13S317*	8	0.113	0.110	0.00	1.000	0.97 (0.36, 2.58)
	9	0.063	0.049	0.15	0.699	0.77 (0.2, 2.98)
	10	0.025	0.085	2.81	0.094	3.64 (0.73, 18.09)
	11	0.263	0.280	0.07	0.791	1.1 (0.55, 2.2)
	12	0.363	0.305	0.61	0.435	0.77 (0.40, 1.48)
	13	0.150	0.098	1.03	0.310	0.61 (0.24, 1.58)
	14	0.025	0.073	2.00	0.157	3.08 (0.6, 15.74)
*D7S820*	7	0.000	0.012	1.01	0.315	Undefined
	8	0.200	0.061	6.94	0.0084	0.26 (0.09, 0.75)
	9	0.125	0.159	0.37	0.543	1.32 (0.54, 3.21)
	10	0.350	0.427	1.01	0.315	1.38 (0.73, 2.60)
	11	0.188	0.146	0.49	0.48	0.74 (0.32, 1.70)
	12	0.125	0.171	0.67	0.41	1.44 (0.60, 3.46)
	13	0.013	0.024	0.31	0.578	1.98 (0.18, 22.28)
*D2S1338*	15	0.000	0.012	1.01	0.315	Undefined
	16	0.075	0.085	0.06	0.806	1.15 (0.37, 3.58)
	17	0.175	0.171	0.01	0.920	0.97 (0.43, 2.19)
	18	0.050	0.061	0.09	0.764	1.23 (0.32, 4.76)
	19	0.125	0.122	0.00	1.000	0.97 (0.38, 2.47)
	20	0.238	0.280	0.39	0.532	1.25 (0.62, 2.53)
	21	0.100	0.037	1.92	0.337	0.39 (0.10, 1.53)
	22	0.038	0.037	0	1.000	1.06 (0.21, 5.43)
	23	0.100	0.049	1.55	0.213	0.46 (0.13, 1.59)
	24	0.025	0.098	3.68	0.04	4.22 (0.87, 20.53)
	25	0.050	0.049	0	1	0.97 (0.23, 4.02)
	26	0.025	0.000	2.08	0.149	0.00 (0.00, ∞)
*D21S11*	27	0.000	0.012	1.01	0.315	Undefined
	28	0.163	0.256	2.14	0.143	1.77 (0.82, 3.84)
	28.2	0.075	0.159	2.73	0.098	2.32 (0.84, 6.44)
	29	0.238	0.037	13.93	0.0002	0.12 (0.03, 0.42)
	29.2	0.150	0.195	0.58	0.446	1.37 (0.60, 3.12)
	30	0.075	0.098	0.06	0.806	1.15 (0.38, 3.49)
	30.2	0.000	0.049	4	0.046	Undefined
	31	0.038	0.049	0.12	0.730	1.32 (0.29, 6.09)
	31.2	0.100	0.024	4	0.040	0.23 (0.05, 1.12)
	32	0.013	0.037	1.02	0.313	3.08 (0.31, 30.24)
	32.2	0.125	0.024	5.98	0.014	0.18 (0.04, 0.85)
	33	0.013	0.049	1.78	0.182	4.05 (0.44, 37.05)
	33.2	0.013	0.000	1.03	0.310	Undefined
	34	0.000	0.012	1.01	0.315	Undefined
*D16S539*	8	0.075	0.061	0.13	0.718	0.8 (0.23, 2.73)
	9	0.138	0.110	0.23	0.632	0.8 (0.31, 2.05)
	10	0.050	0.049	0	1	0.99 (0.23, 4.02)
	11	0.425	0.415	0.07	0.791	0.92 (0.49, 1.72)
	12	0.213	0.159	0.78	0.377	0.7 (0.31, 1.56)
	13	0.088	0.183	2.1	0.147	2.01 (0.77, 5.25)
	14	0.013	0.024	0.34	0.560	2.03 (0.18, 22.85)
*D18S51*	11	0.038	0.000	3.13	0.077	Undefined
	12	0.113	0.159	0.73	0.393	1.49 (0.6, 3.71)
	13	0.175	0.122	2.39	0.122	0.51 (0.22, 1.19)
	13.2	0.050	0.024	0.74	0.390	0.48 (0.09, 2.70)
	14	0.100	0.134	0.46	0.498	1.39 (0.53, 3.66)
	14.2	0.038	0.073	0.98	0.322	2.03 (0.49, 8.41)
	15	0.050	0.061	0.09	0.764	1.23 (0.32, 4.76)
	16	0.163	0.207	0.54	0.462	1.35 (0.61, 3.00)
	17	0.100	0.061	0.84	0.360	0.58 (0.18, 1.86)
	18	0.075	0.061	0.13	0.718	0.80 (0.23, 2.73)
	19	0.063	0.061	0	1	0.97 (0.27, 3.49)
	20	0.025	0.024	0	1	0.98 (0.13, 7.13)
	21	0.013	0.000	1.03	0.310	Undefined
	23	0.000	0.012	1.01	0.315	Undefined
*CSF1PO*	9	0.013	0.061	2.67	0.102	5.13 (0.59, 44.93)
	10	0.275	0.317	0.34	0.560	1.22 (0.62, 2.40)
	11	0.425	0.244	5.98	0.0144	0.44 (0.22, 0.86)
	12	0.263	0.305	0.36	0.549	1.23 (0.62, 2.44)
	13	0.025	0.061	1.27	0.260	2.53 (0.48, 13.44)
		0.000	0.012	1.01	0.315	Undefined
*FGA*	18	0.000	0.024	1.98	0.159	Undefined
	19	0.050	0.049	0	1	0.97 (0.23, 4.02)
	20	0.113	0.110	0	1	0.97 (0.36, 2.58)
	21	0.113	0.073	0.75	0.386	0.62 (0.21, 1.83)
	22	0.113	0.183	1.59	0.207	1.77 (0.73, 4.32)
	23	0.200	0.207	0.01	0.920	1.05 (0.49, 2.26)
	24	0.188	0.134	0.86	0.354	0.67 (0.29, 1.56)
	25	0.150	0.146	0	1	0.97 (0.41, 2.31)
	26	0.025	0.061	1.27	0.260	2.53 (0.48, 13.44)
	27	0.025	0.000	2.08	0.150	Undefined
	28	0.013	0.012	0	1	0.98 (0.06, 15.94)
	29	0.013	0.000	1.03	0.310	Undefined

^1^ Student’s *t*-test (2-tailed) for continuous variables, Pearson’s χ^2^ test for categorical variables (Fisher’s exact test for low numbers).

**Table 4:** Microsatellite instability and loss of heterozygosity in breast cancer patients.

Patient	Age (Years)	Stage	DNA Samples	MSI Detected in	LOH Detected in	RT-(Cf-DNA) ^1^
2	40	4	Plasma		*FGA*	40
4	43	3	Plasma		*D7S820, D16S539*	41
8	70	2	Plasma	*D13S317, D16S539*		71.4
9	42	2	Plasma		*D18S51*	68.1
10	44	2	Plasma		*CSF1P0*	64.9
11	45	4	Plasma	*D2S1338*	*D13S317*	120
13	62	1	Plasma		*CSF1P0*	58.7
15	85	3	Blood-plasma	*CSF1P0*	*FGA*	99.1
16	31	4	Plasma	*D13S317, D7S820, D18S51*	*FGA*	49.9
18	35	2	Plasma		*D2S1338*	62
20	57	2	Blood-plasma	*CSF1P0*	*CSF1P0*	156
30	42	3	Plasma		*FGA*	35.5
36	33	2	Plasma		*D21S11*	58
38	47	2	Plasma		*D21S11*	34
39	49	1	Plasma	*D2S1338*		44.9

MSI: microsatellite instability; LOH: loss of heterozygosity. ^1^ CfDNA concentration (ng/mL) as determined by RT-PCR.

## Data Availability

The authors confirm the availability of the data and materials under reasonable request.

## Supplementary Materials

The following supporting information can be downloaded at: https://www.mdpi.com/article/10.3390/genes13040590/s1, Supplementary Table S1. STR profiling results of CfDNA and genomic DNA extracted from healthy control samples. Supplementary Table S2. STR profiling results of CfDNA and genomic DNA of BC patients. Figure S1. Electropherogram generated from short tandem repeat profiling of cfDNA and genomic DNA extracted from BC patients.

## Abbreviations

BC	Breast cancer
*BRCA1*	Breast Cancer 1 gene
*BRCA2*	Breast Cancer 2 gene
BMI	Body mass index
*CDK6*	Cyclin Dependent Kinase 6 gene
cfDNA	Cell-free DNA
ER	Estrogen receptor
HER2	Human epidermal growth factor receptor2
*IGHG3*	Immunoglobulin heavy constant γ 3 gene
KSA	Kingdom of Saudi Arabia
LOH	Loss of heterozygosity
MMR	Mismatch repair system
MSI	Microsatellite instability
OR	Odds ratio
PR	Progesterone receptor
PBD	Proliferative breast disease
*RPS9*	Ribosomal Protein S9 gene
SDS	System Sequence Detection Software
STR	Short tandem repeat
