# Supplementary material for: Utility of Circulating Cell-Free DNA in Assessing Microsatellite Instability and Loss of Heterozygosity in Breast Cancer Using Human Identification Approach
Source: Genes (Basel). 2022 Mar 25;13(4):590. doi: 10.3390/genes13040590 (PMC9027523; doi:10.3390/genes13040590)
Supplement: Supplementary file 1 [file genes-13-00590-s001.zip › Supplementary Figure S1.pdf]

Applied Biosystems
GeneMapper® ID-X 1.1

Project: Noura_breast_cancer_grp2

Sample Name	SOS	SQ	SSPK	MIX	CGQ
A20					

A20

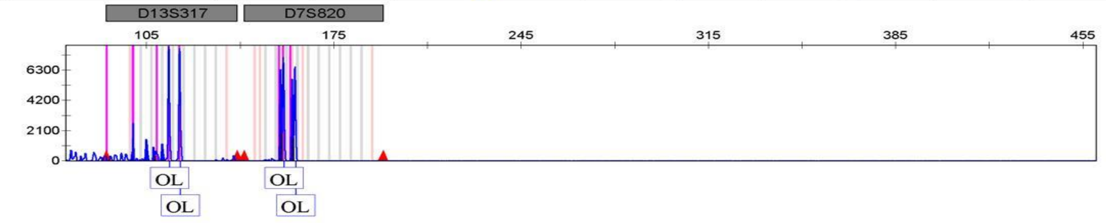

A20

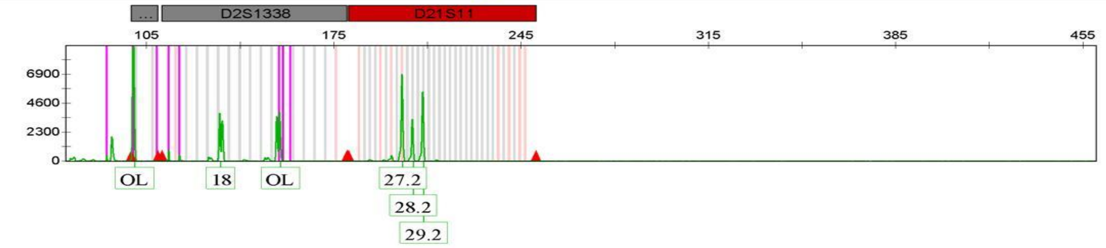

A20

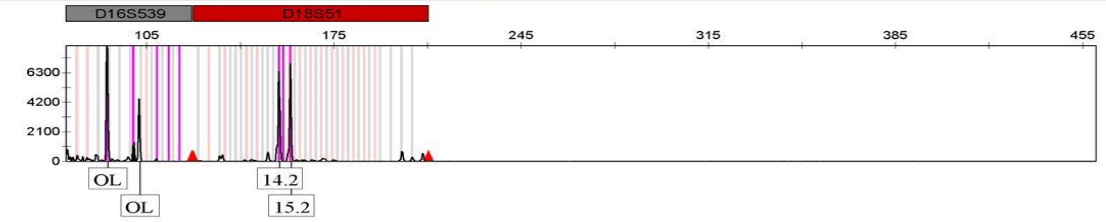

Applied Biosystems
GeneMapper® ID-X 1.1

Project: Noura_breast_cancer_grp2

Sample Name	SOS	SQ	SSPK	MIX	CGQ
A20					

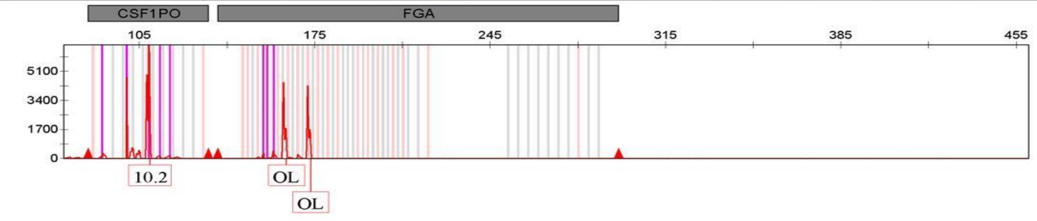

Sample Name	SOS	SQ	SSPK	MIX	CGQ
2B					

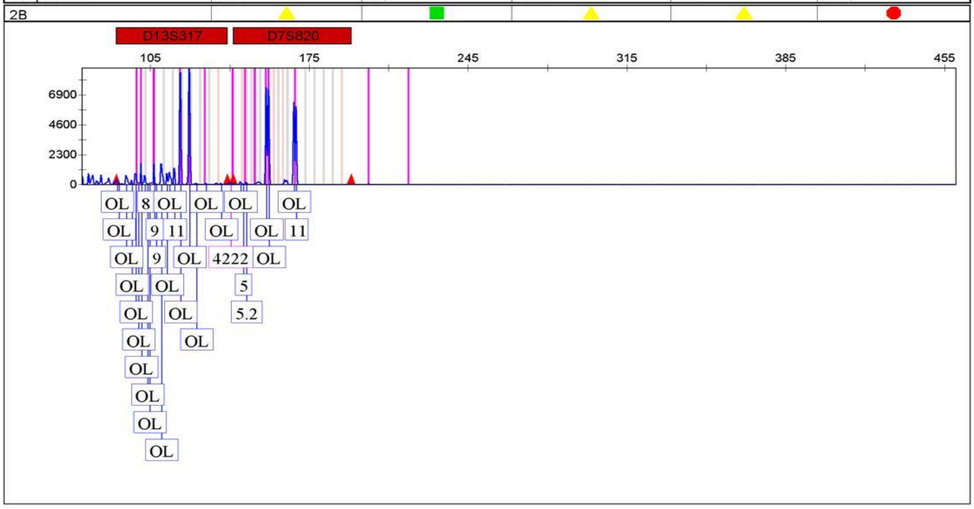

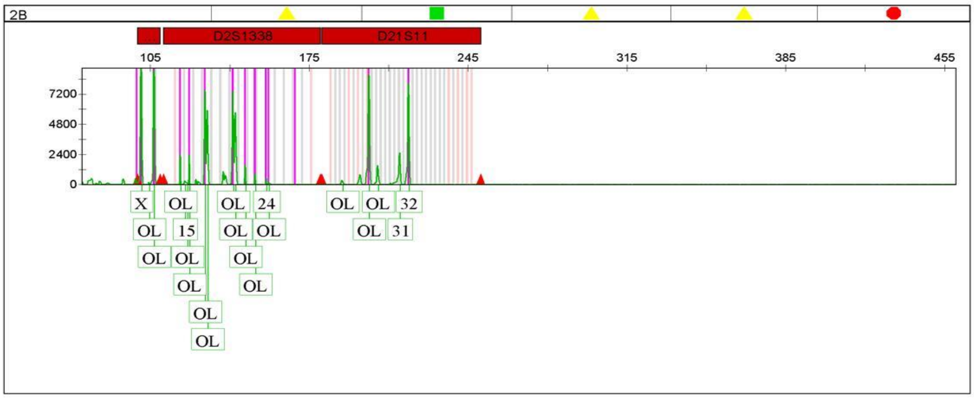

Applied Biosystems
GeneMapper® ID-X 1.1

Project: noura_breast_cancer

Sample Name	SOS	SQ	SSPK	MIX	CGQ
2B					

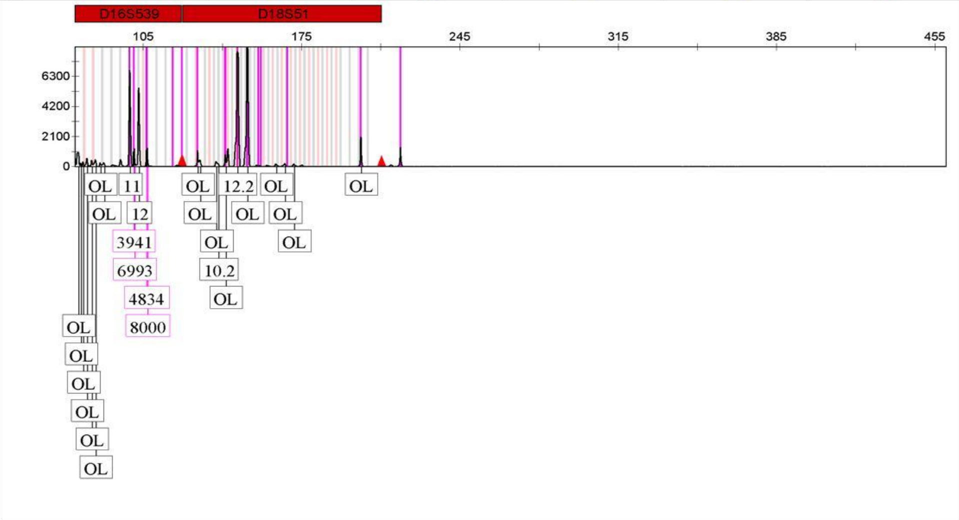

2B					

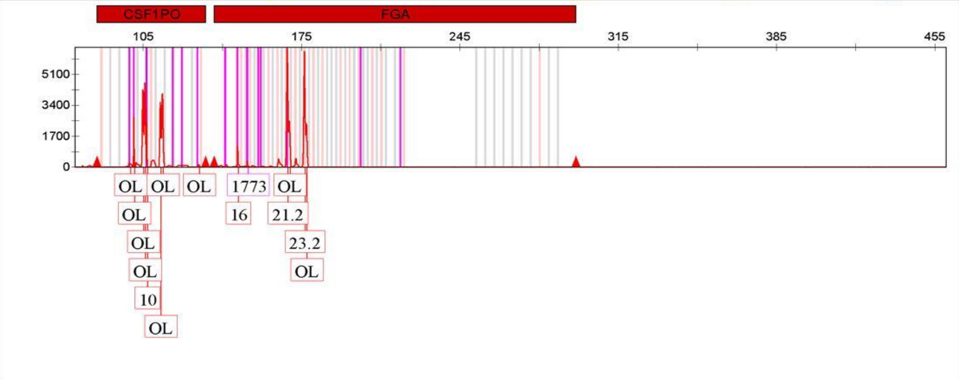

Applied Biosystems
GeneMapper® ID-X 1.1

Project: noura_breast_cancer

Sample Name	SOS	SQ	SSPK	MIX	CGQ
A60					

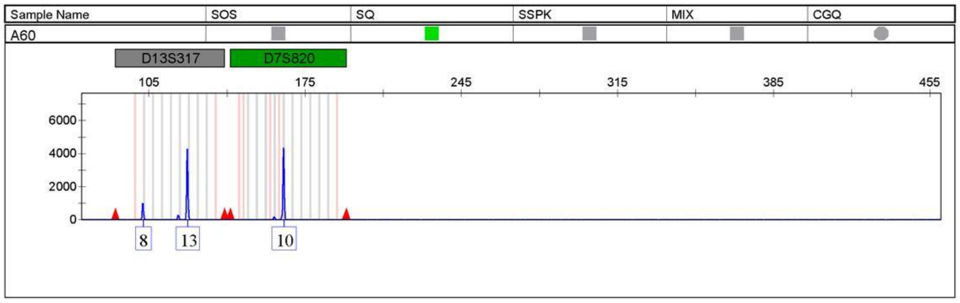

A60					

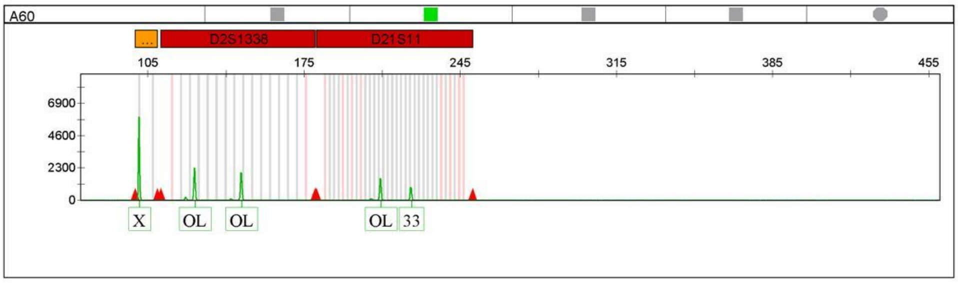

A60					

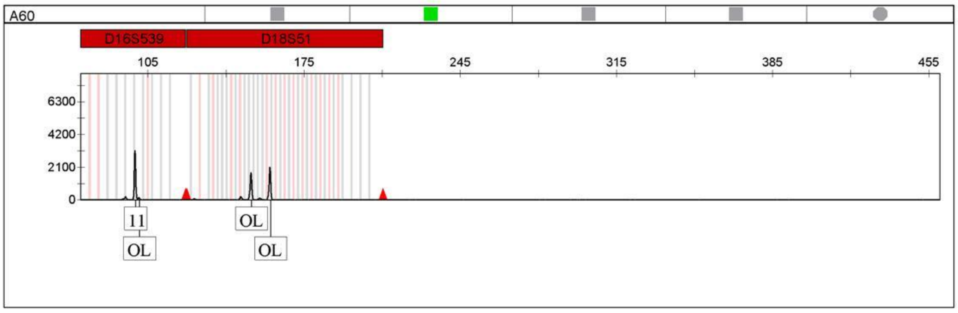

A60					

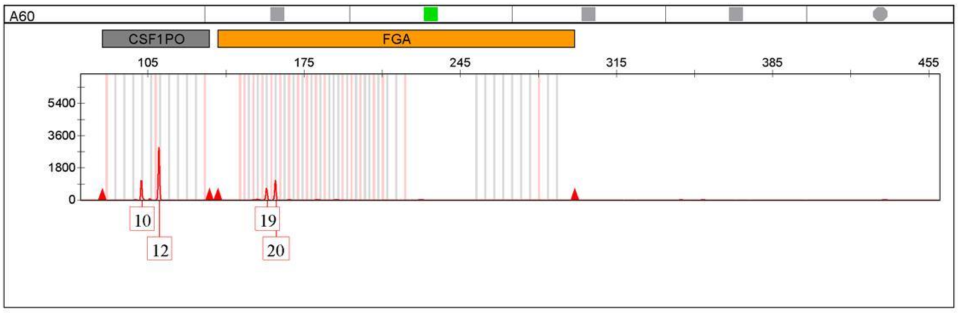

GeneMapper® ID-X 1.1

Project: noura_breast_cancer

Sample Name	SOS	SQ	SSPK	MIX	CGQ
A89					

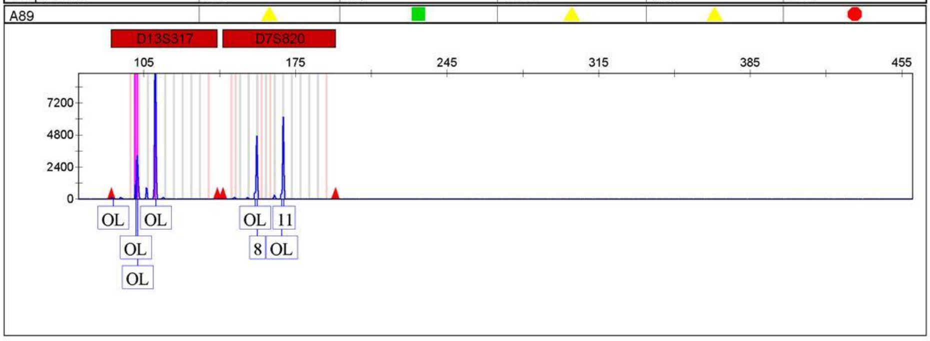

A89					

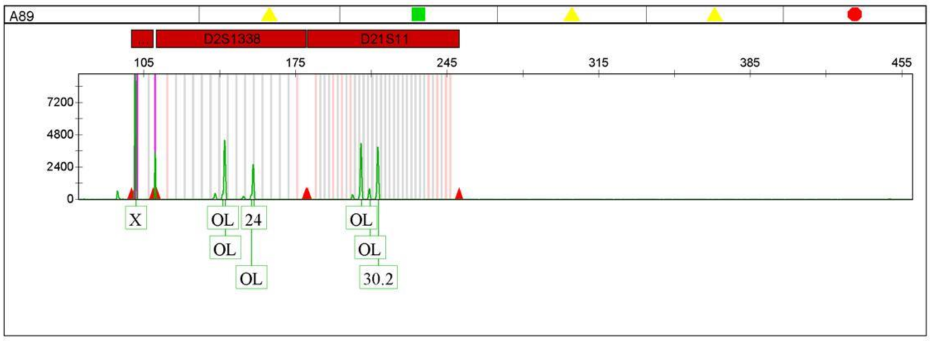

A89					

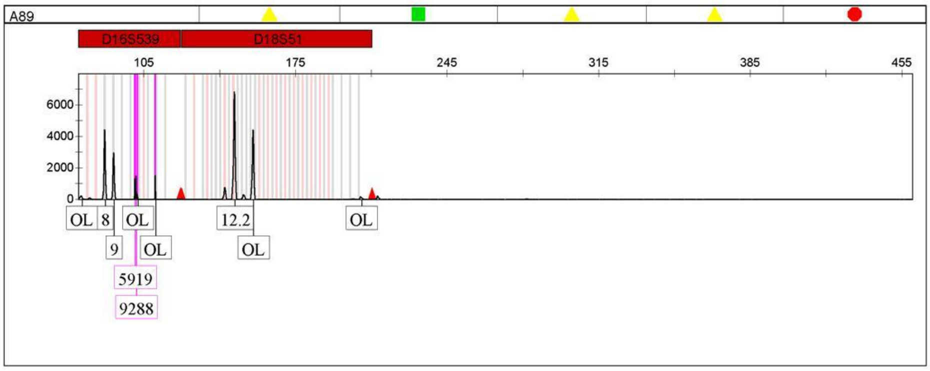

Sample Name	SOS	SQ	SSPK	MIX	CGQ
A89					

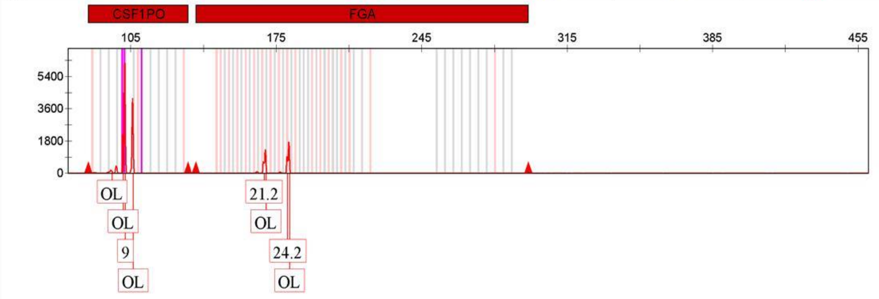

B17					

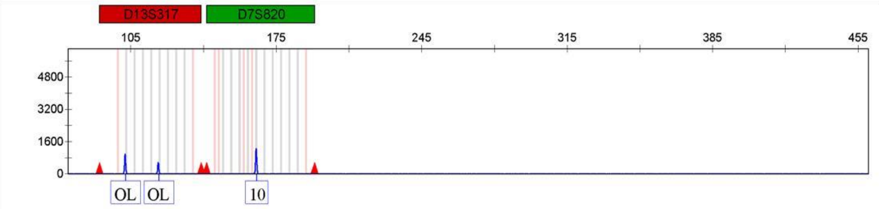

B17					

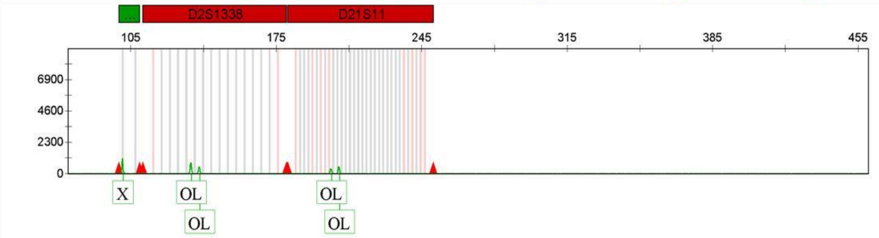

Applied Biosystems
GeneMapper® ID-X 1.1

Project: noura_breast_cancer

Sample Name	SOS	SQ	SSPK	MIX	CGQ
B17					

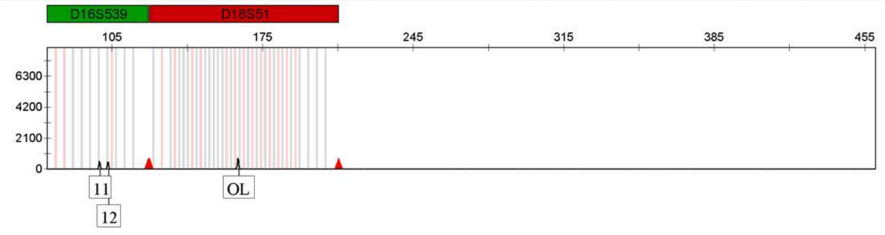

Sample Name	SOS	SQ	SSPK	MIX	CGQ
B17					

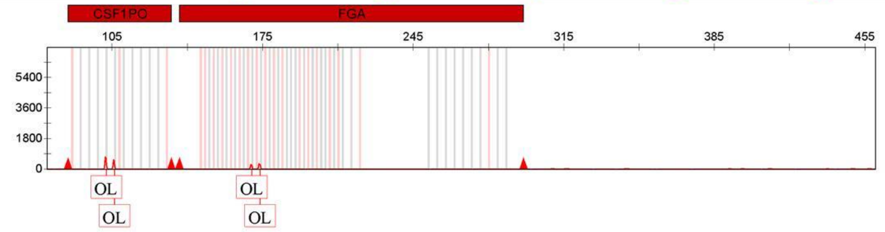

Sample Name	SOS	SQ	SSPK	MIX	CGQ
B35					

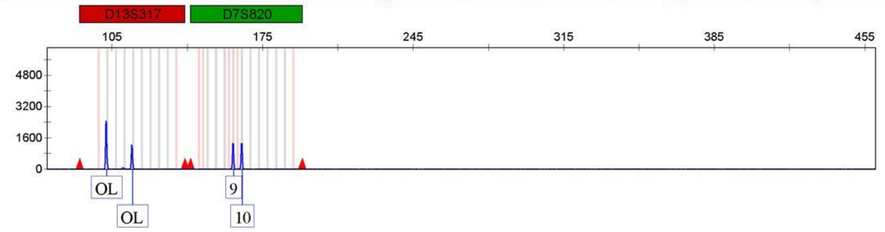

Sample Name	SOS	SQ	SSPK	MIX	CGQ
B35					

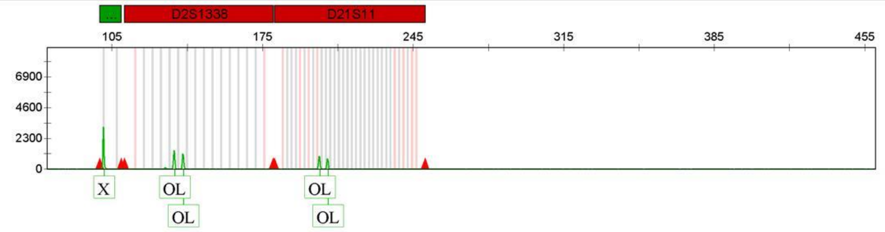

Applied Biosystems
GeneMapper® ID-X 1.1

Project: noura_breast_cancer

Sample Name	SOS	SQ	SSPK	MIX	CGQ
B35					

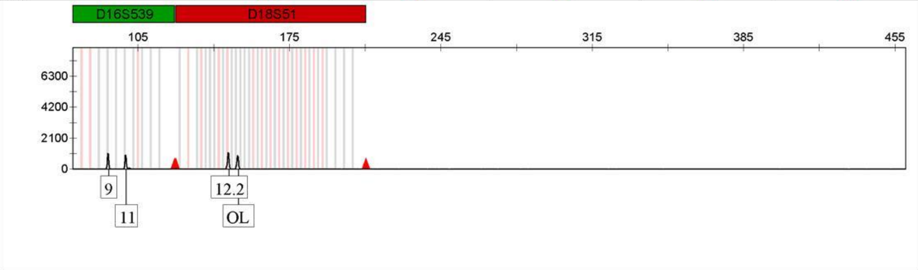

B35

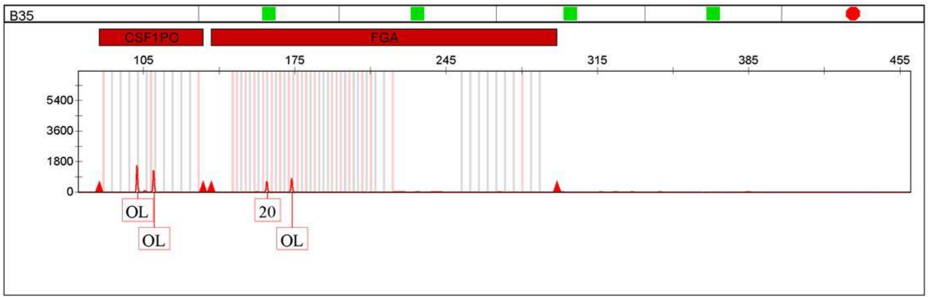

1

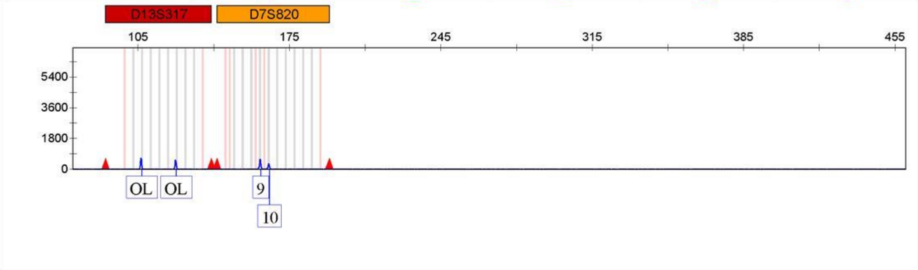

1

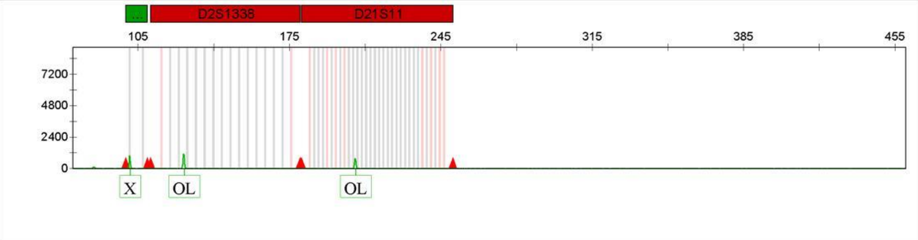

Applied Biosystems
GeneMapper® ID-X 1.1

Project: noura_breast_cancer

Sample Name	SOS	SQ	SSPK	MIX	CGQ
1					

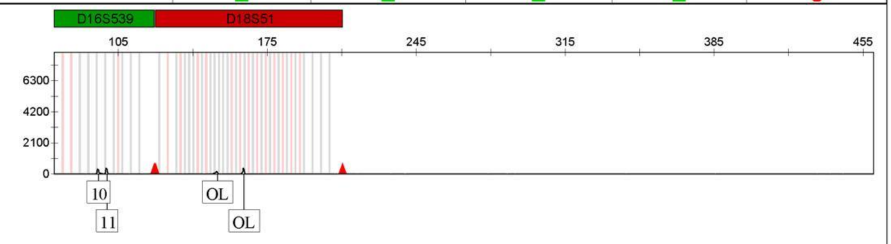

Sample Name	SOS	SQ	SSPK	MIX	CGQ
1					

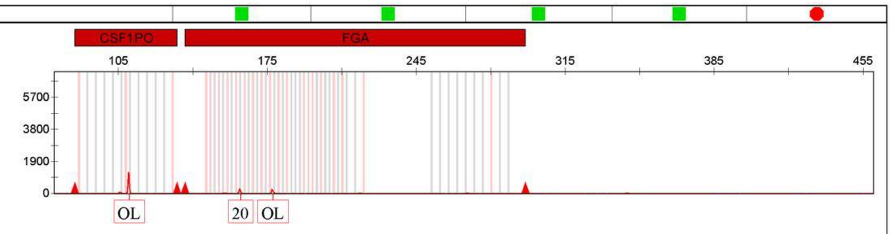

Sample Name	SOS	SQ	SSPK	MIX	CGQ
10					

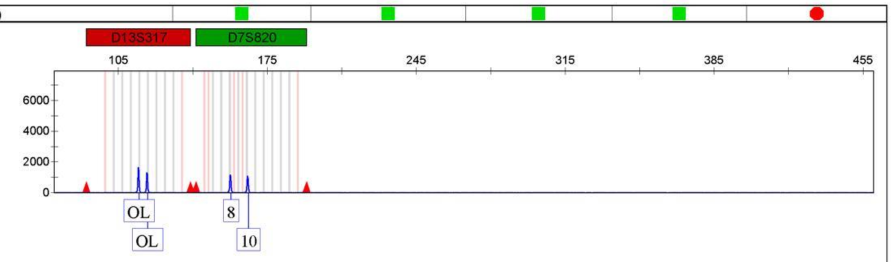

Sample Name	SOS	SQ	SSPK	MIX	CGQ
10					

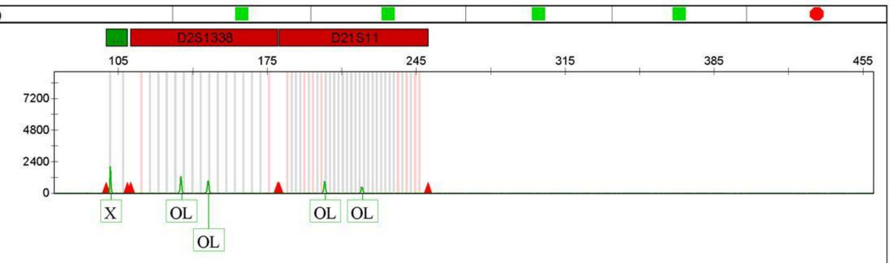

Sample Name	SOS	SQ	SSPK	MIX	CGQ
10					

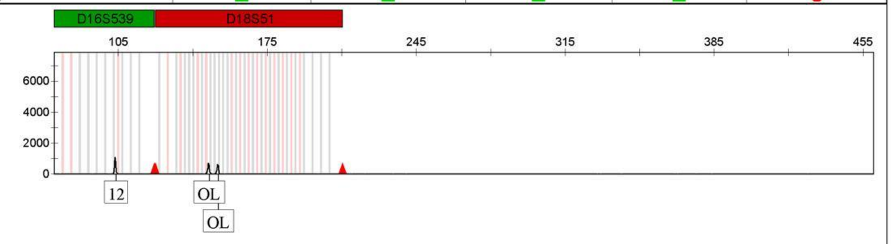

Sample Name	SOS	SQ	SSPK	MIX	CGQ
10					

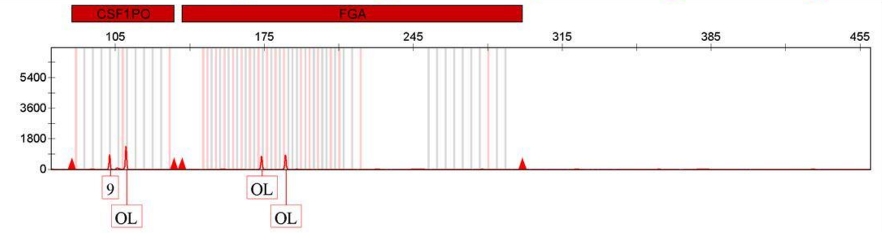

Sample Name	SOS	SQ	SSPK	MIX	CGQ
18					

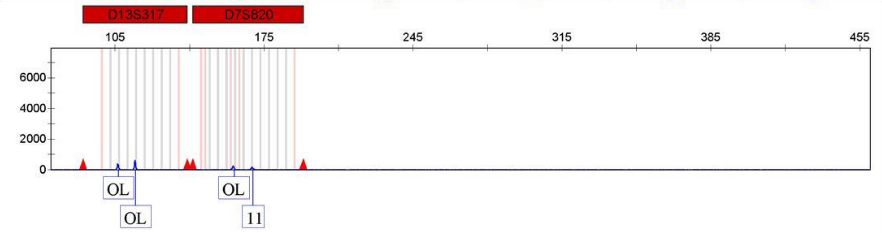

Sample Name	SOS	SQ	SSPK	MIX	CGQ
18					

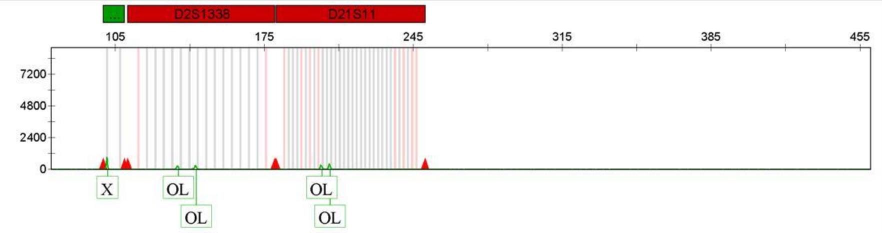

Sample Name	SOS	SQ	SSPK	MIX	CGQ
18					

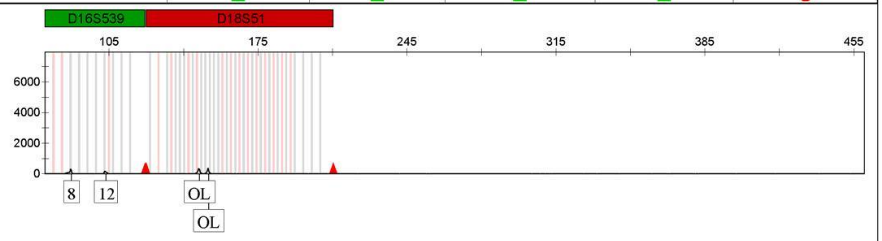

Sample Name	SOS	SQ	SSPK	MIX	CGQ
18					

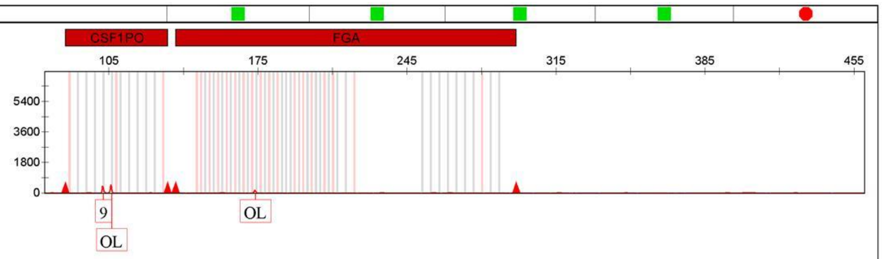

Sample Name	SOS	SQ	SSPK	MIX	CGQ
26					

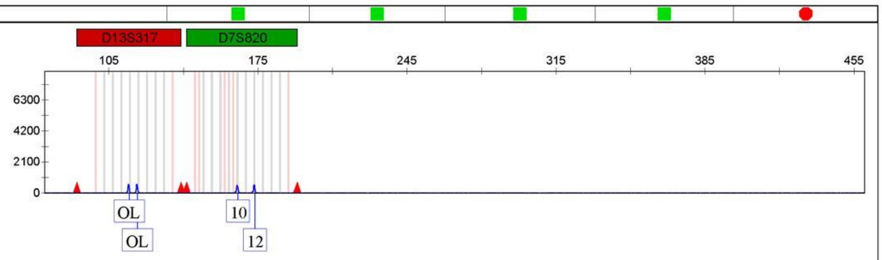

Sample Name	SOS	SQ	SSPK	MIX	CGQ
26					

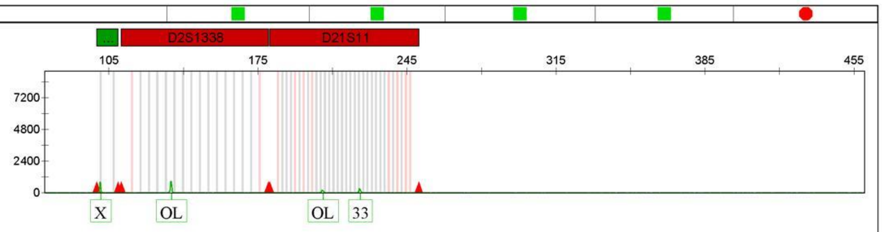

Sample Name	SOS	SQ	SSPK	MIX	CGQ
26					

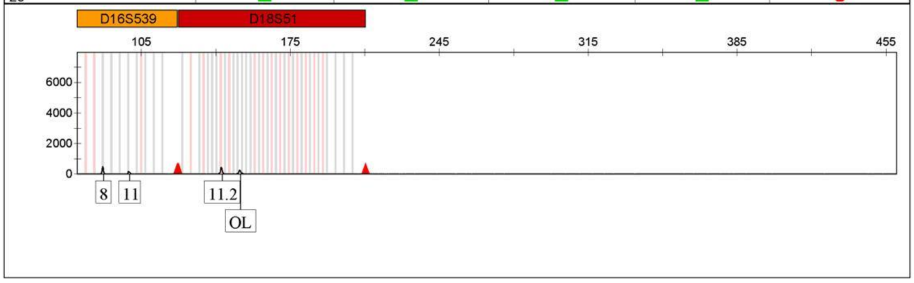

Sample Name	SOS	SQ	SSPK	MIX	CGQ
26					

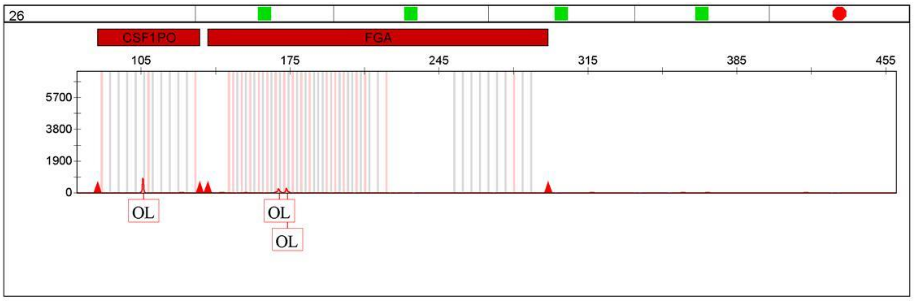

Sample Name	SOS	SQ	SSPK	MIX	CGQ
A80					

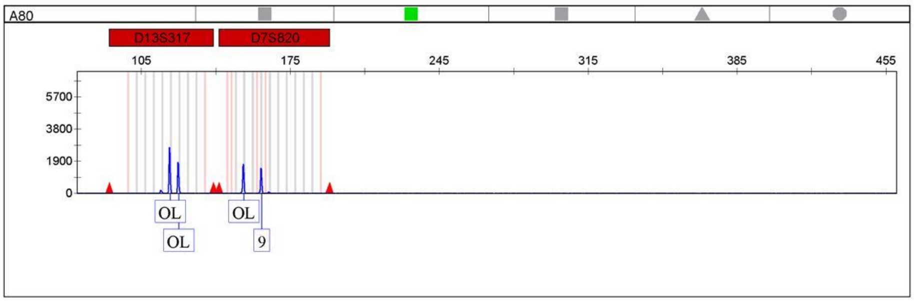

Applied Biosystems
GeneMapper® ID-X 1.1

Project: noura_breast_cancer

Sample Name	SOS	SQ	SSPK	MIX	CGQ
A80					

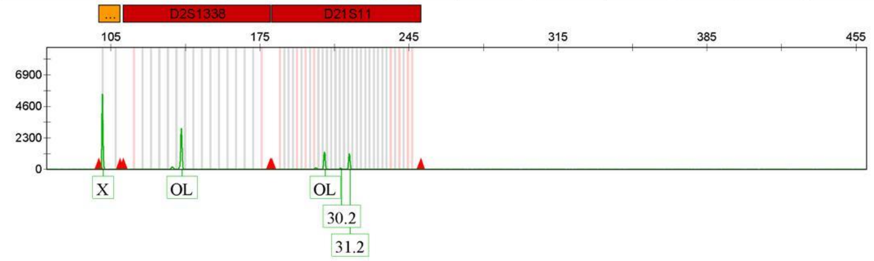

A80

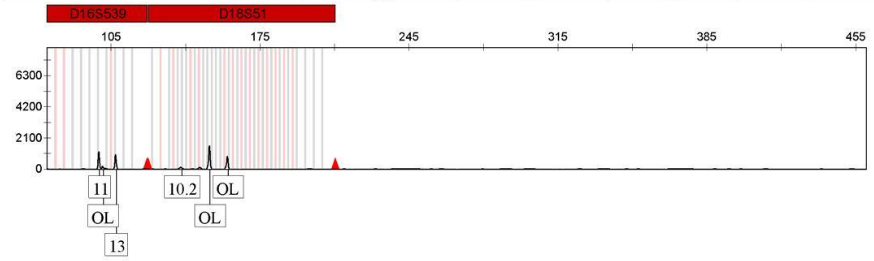

A80

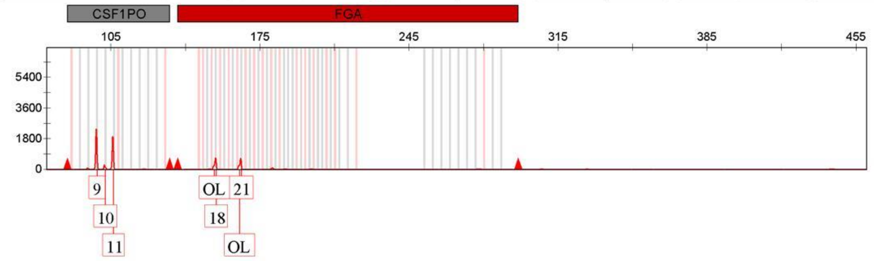

Applied Biosystems
GeneMapper® ID-X 1.1

Project: noura_breast_cancer

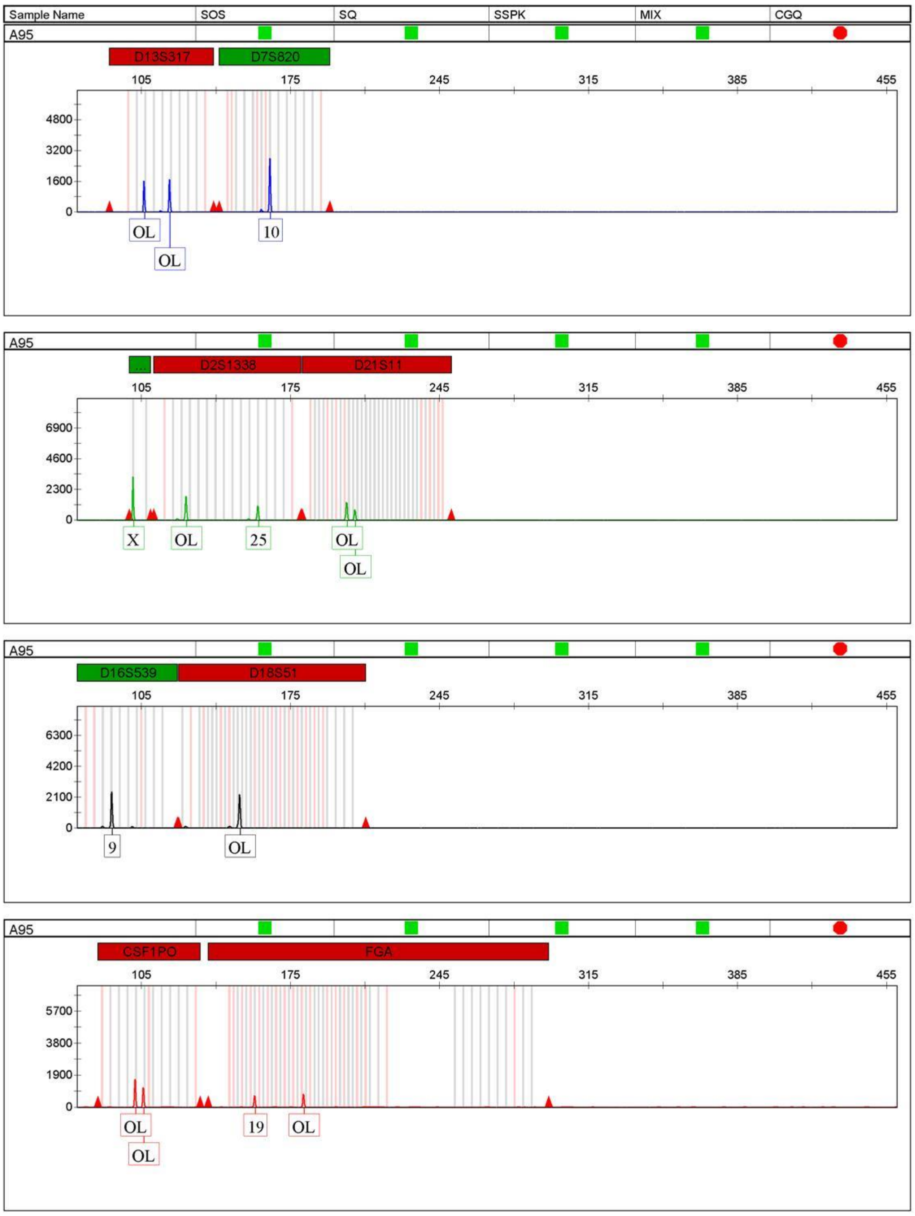

Sample Name	SOS	SQ	SSPK	MIX	CGQ
B39					

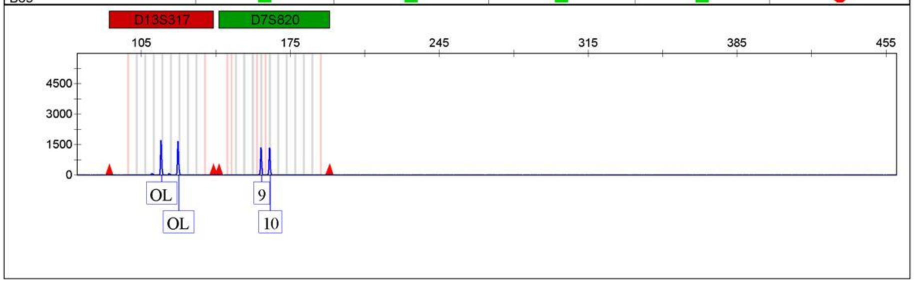

B39					

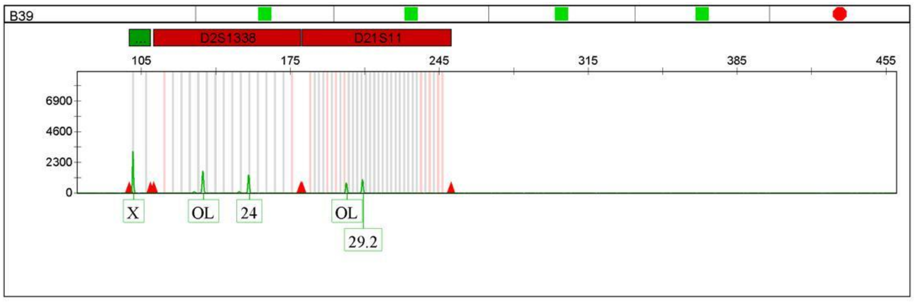

B39					

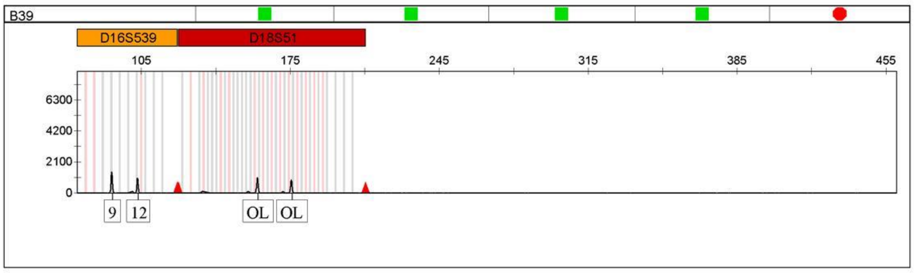

B39					

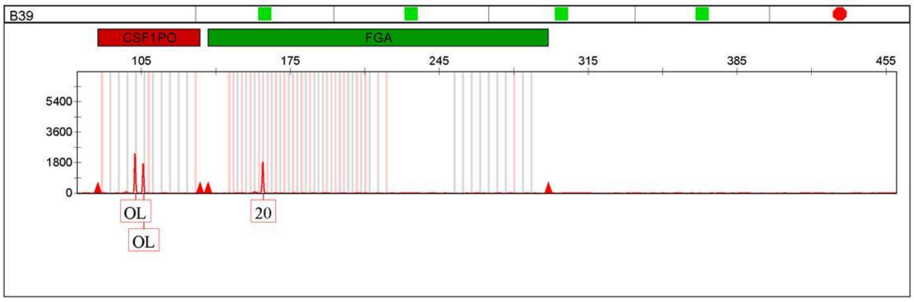

Sample Name	SOS	SQ	SSPK	MIX	CGQ
6					

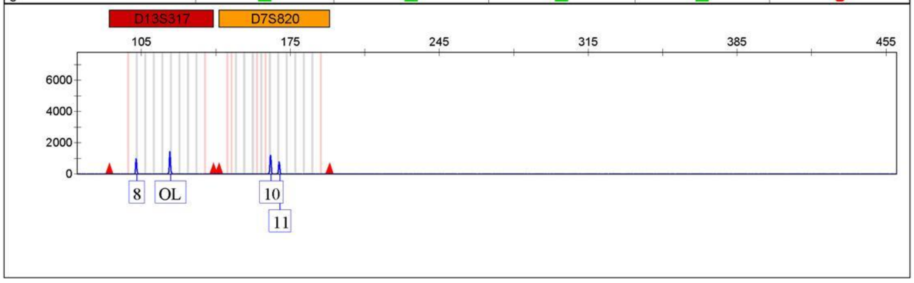

6					

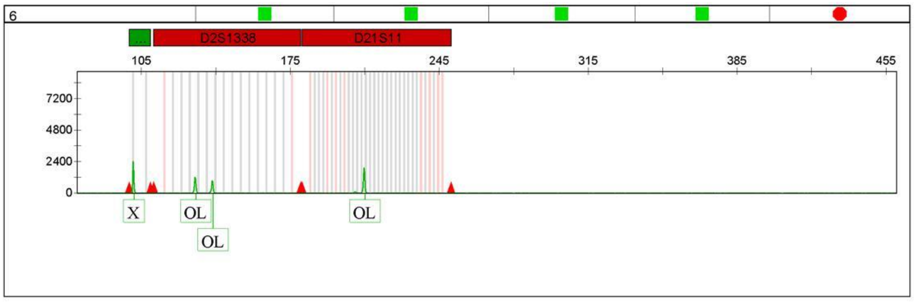

6					

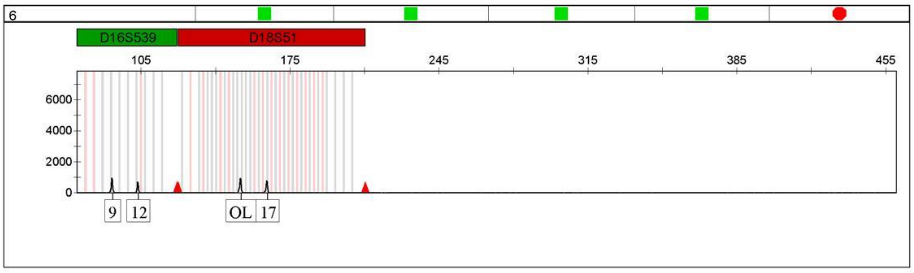

6					

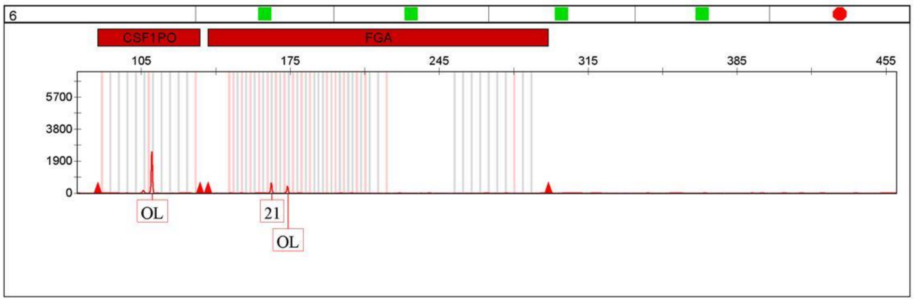

Sample Name	SOS	SQ	SSPK	MIX	CGQ
14					

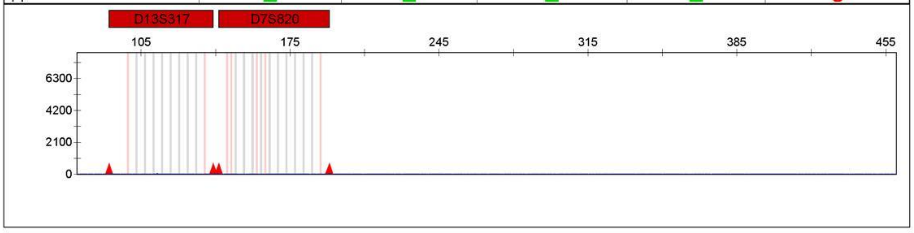

14					

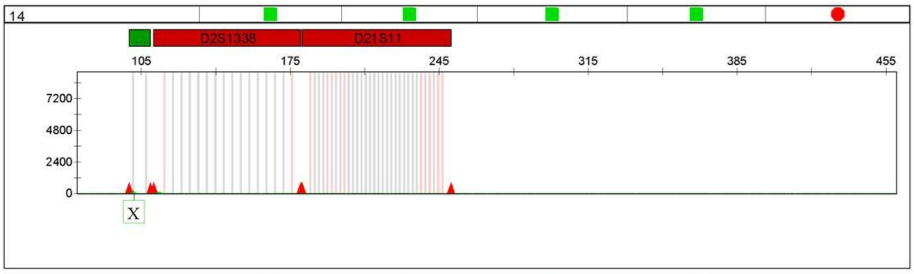

14					

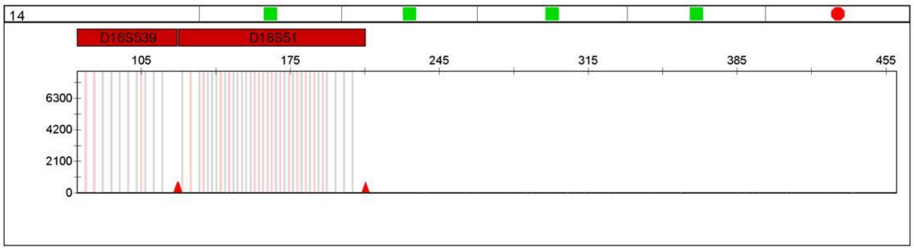

14					

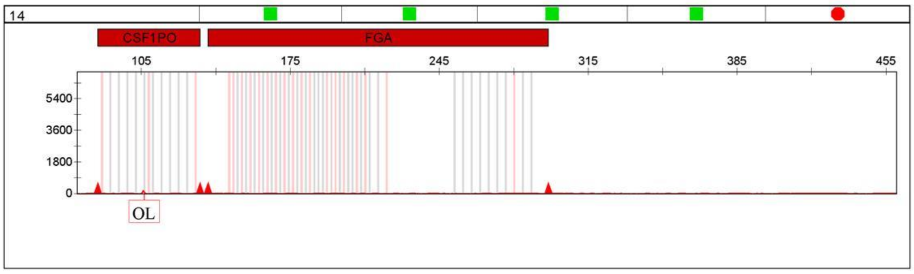

Applied Biosystems
GeneMapper® ID-X 1.1

Project: noura_breast_cancer

Sample Name	SOS	SQ	SSPK	MIX	CGQ
22					

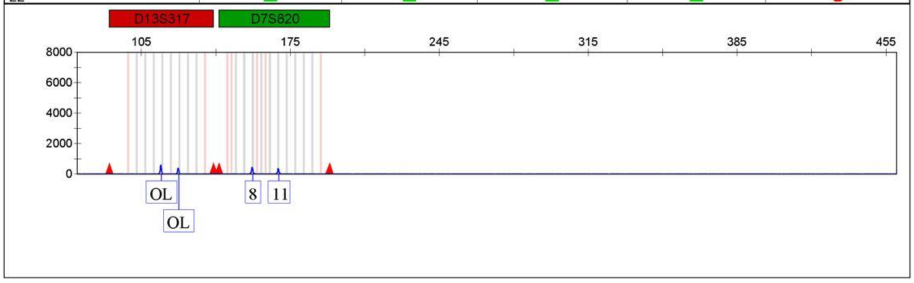

22

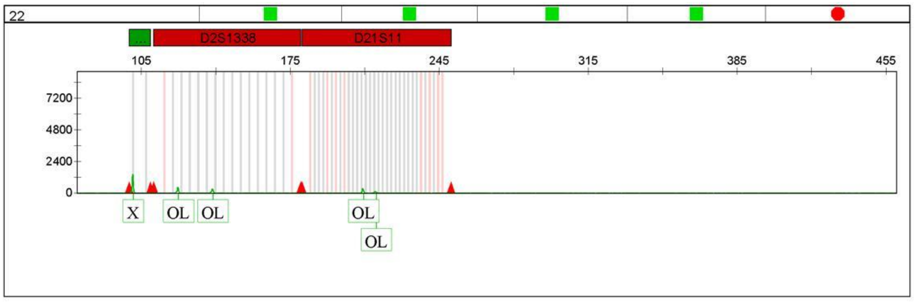

22

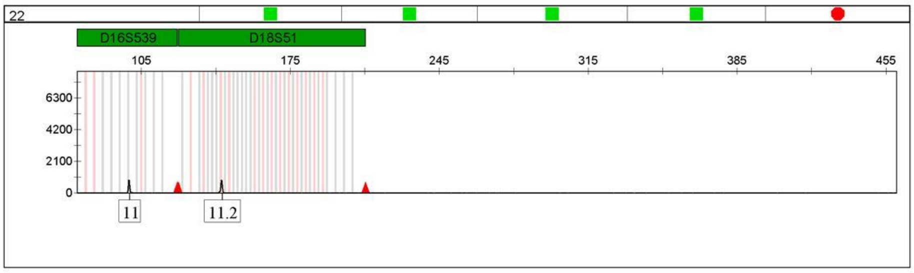

22

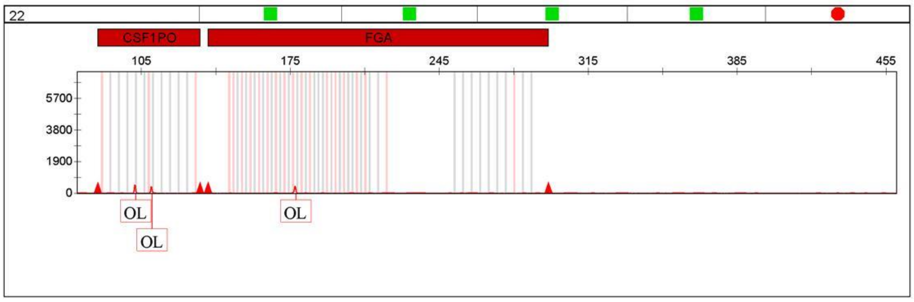

Applied Biosystems
GeneMapper® ID-X 1.1

Project: noura_breast_cancer

Sample Name	SOS	SQ	SSPK	MIX	CGQ
3B					

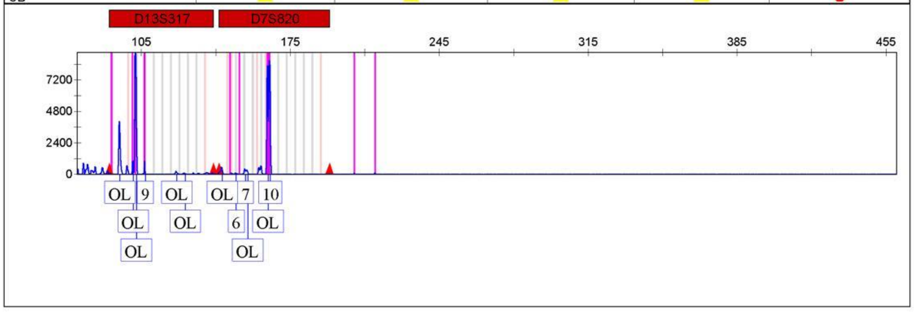

Sample Name	SOS	SQ	SSPK	MIX	CGQ
3B					

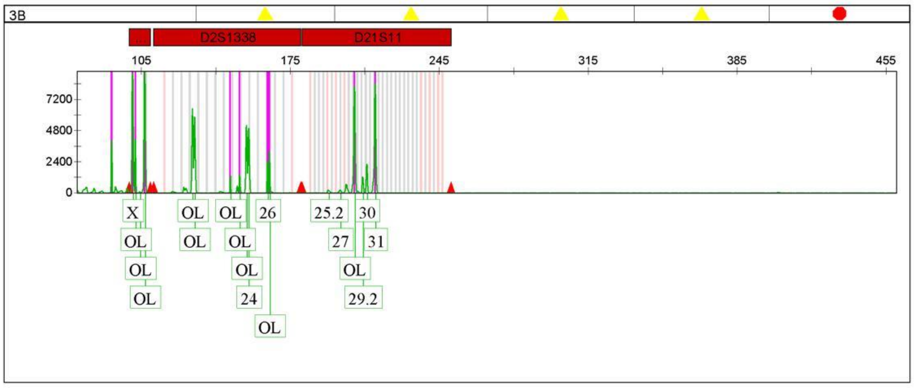

Sample Name	SOS	SQ	SSPK	MIX	CGQ
3B					

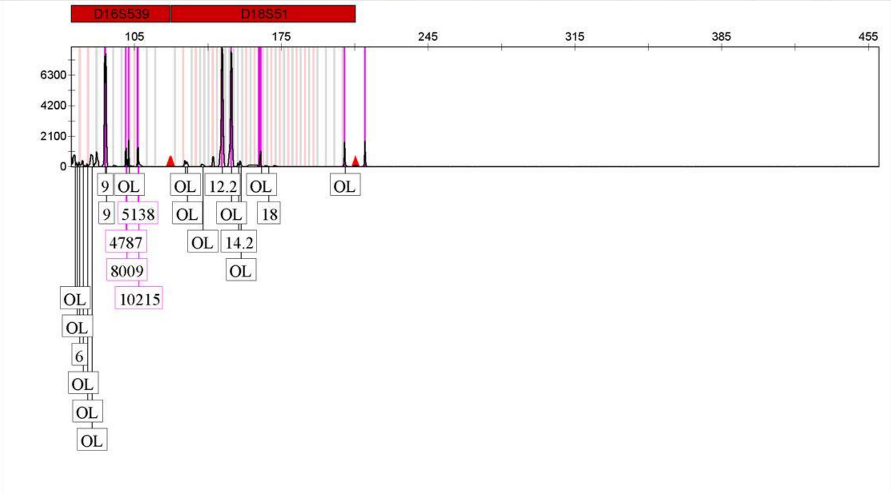

3B

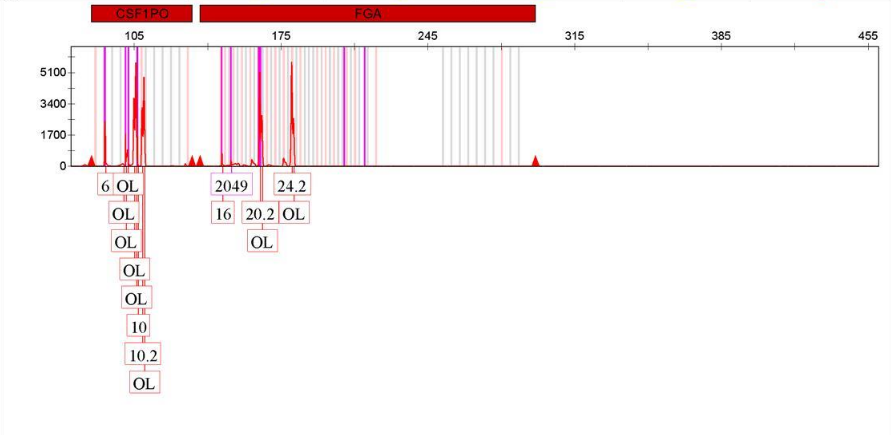

Sample Name	SOS	SQ	SSPK	MIX	CGQ

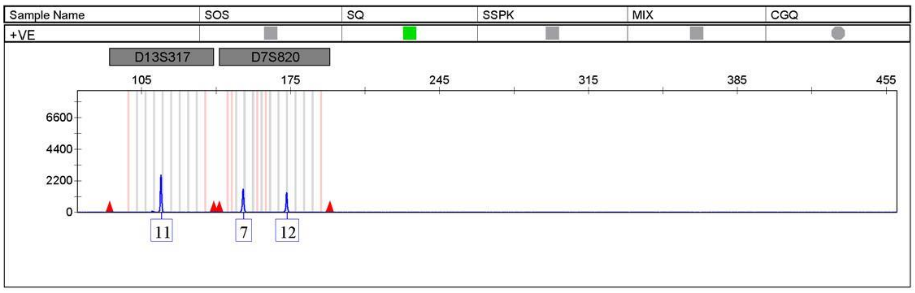

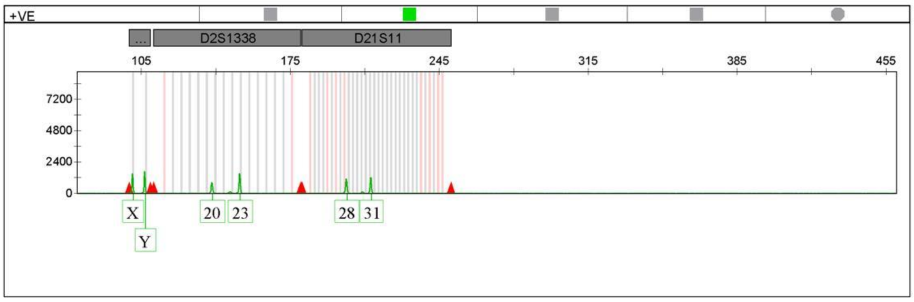

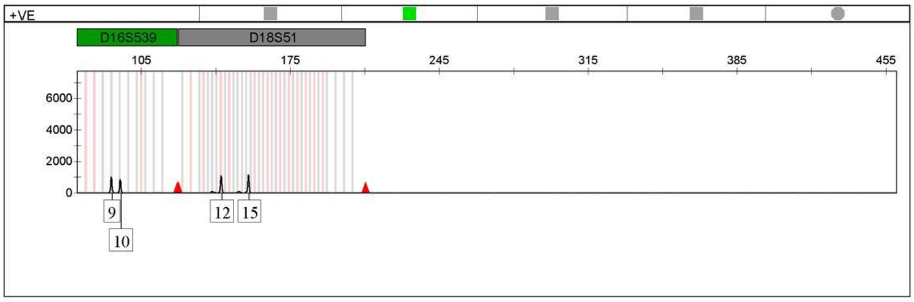

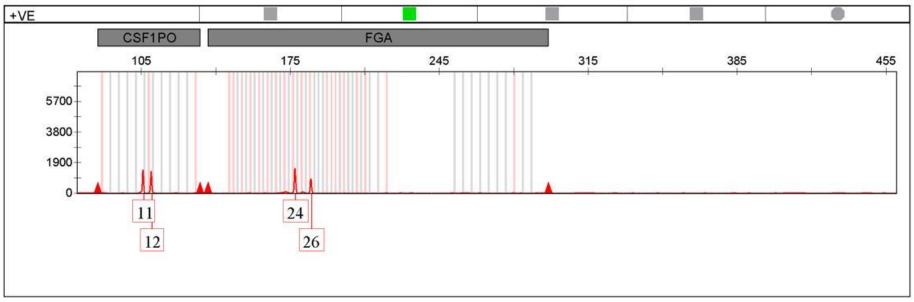

Applied Biosystems
GeneMapper® ID-X 1.1

Project: noura_breast_cancer

Sample Name	SOS	SQ	SSPK	MIX	CGQ
A61					

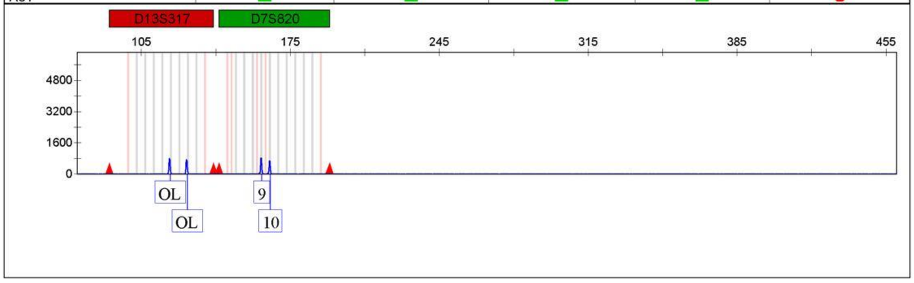

A61

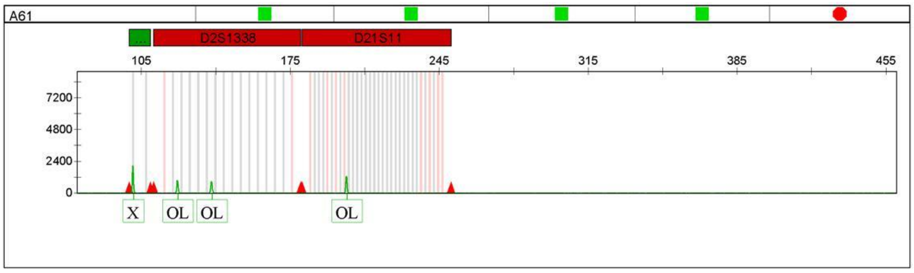

A61

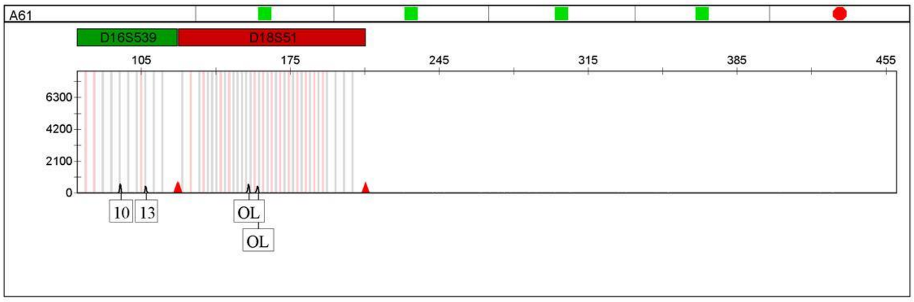

A61

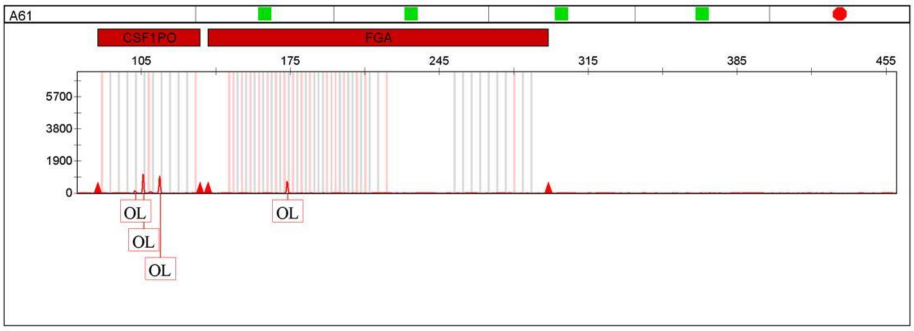

Project: noura_breast_cancer

Sample Name	SOS	SQ	SSPK	MIX	CGQ
A90					

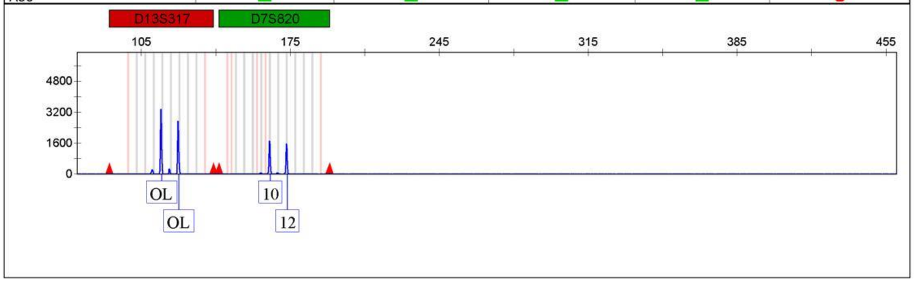

A90					

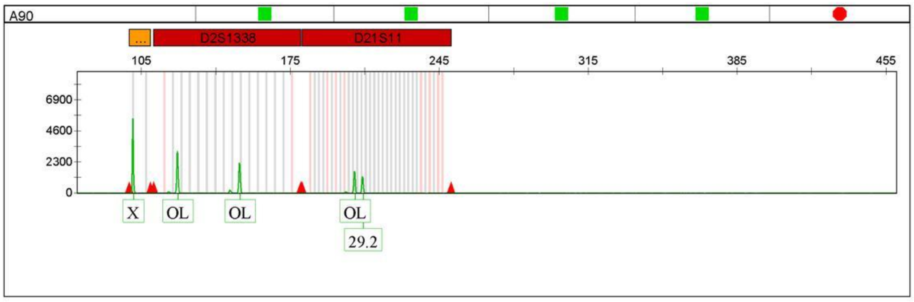

A90					

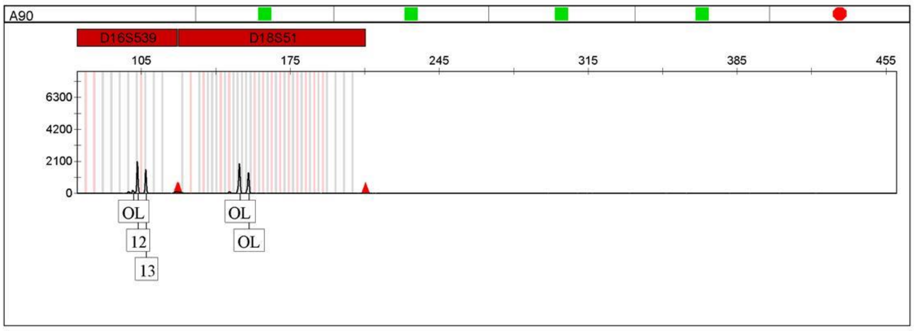

Applied Biosystems
GeneMapper® ID-X 1.1

Project: noura_breast_cancer

Sample Name	SOS	SQ	SSPK	MIX	CGQ
A90					

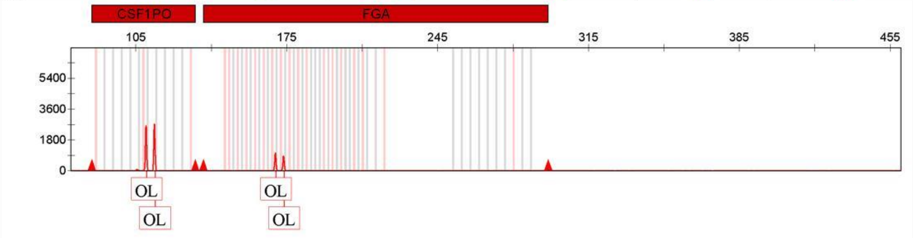

Sample Name	SOS	SQ	SSPK	MIX	CGQ
B23					

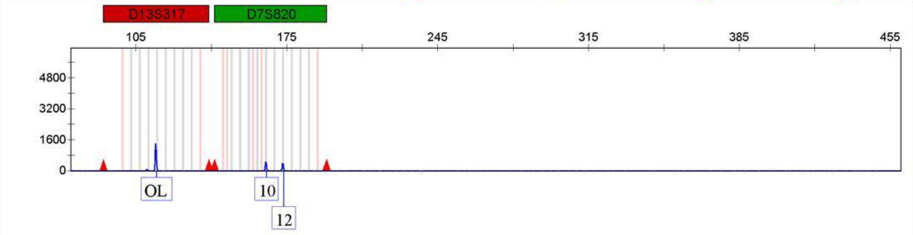

Sample Name	SOS	SQ	SSPK	MIX	CGQ
B23					

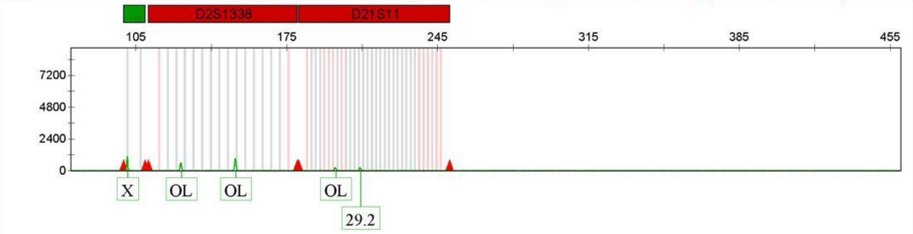

Sample Name	SOS	SQ	SSPK	MIX	CGQ
B23					

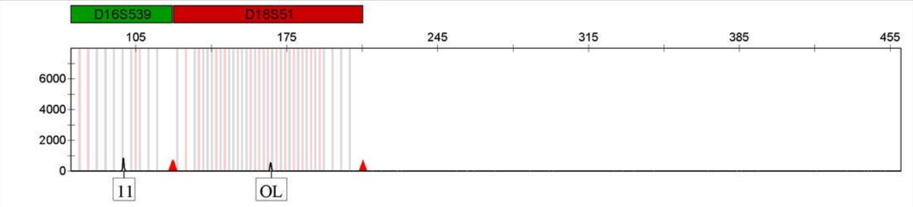

Sample Name	SOS	SQ	SSPK	MIX	CGQ
B23					

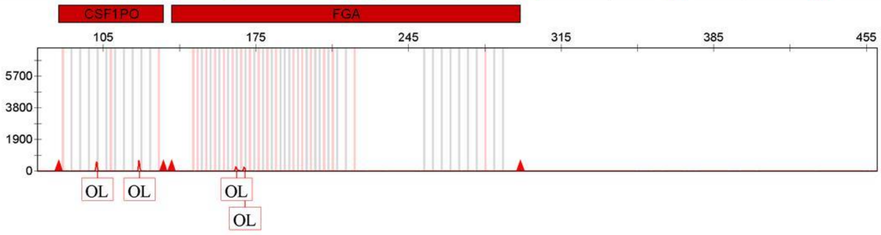

B36					

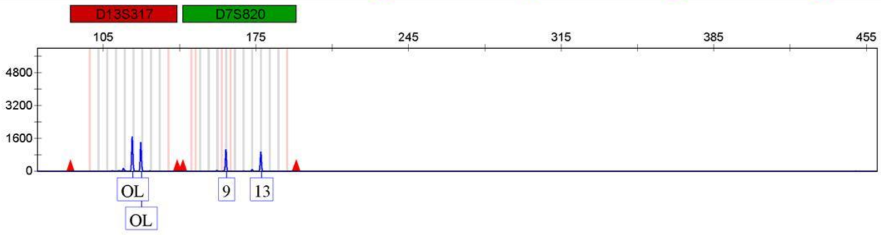

B36					

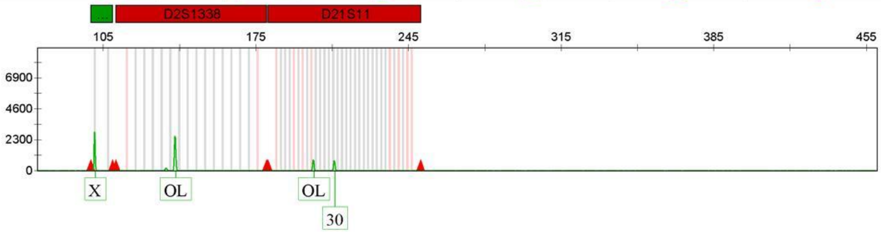

B36					

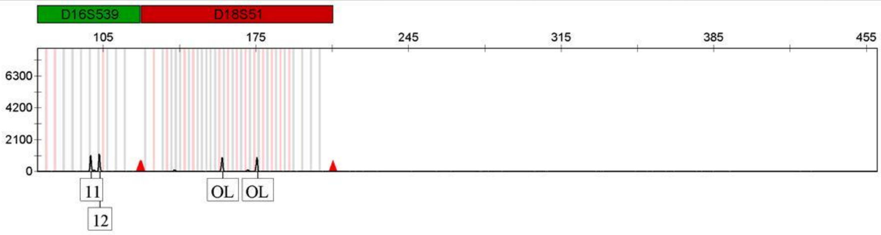

Sample Name	SOS	SQ	SSPK	MIX	CGQ
B36					

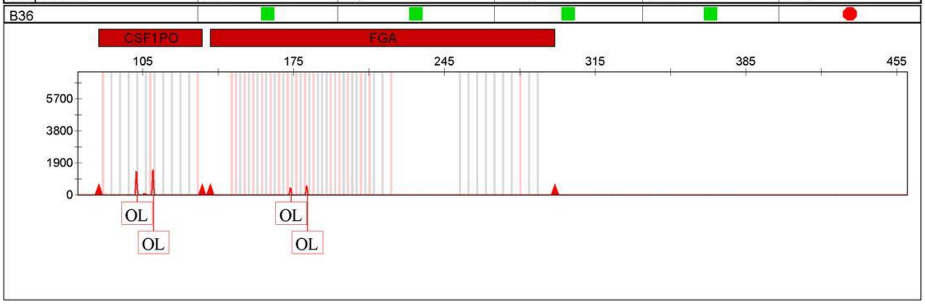

3					

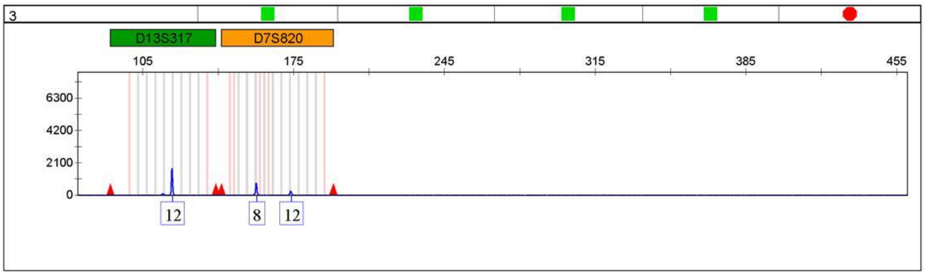

3					

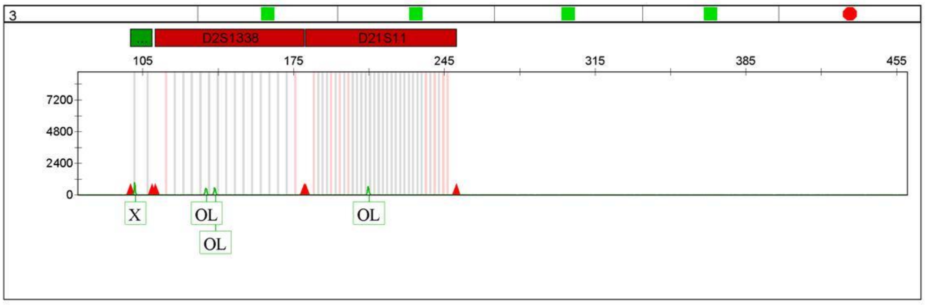

3					

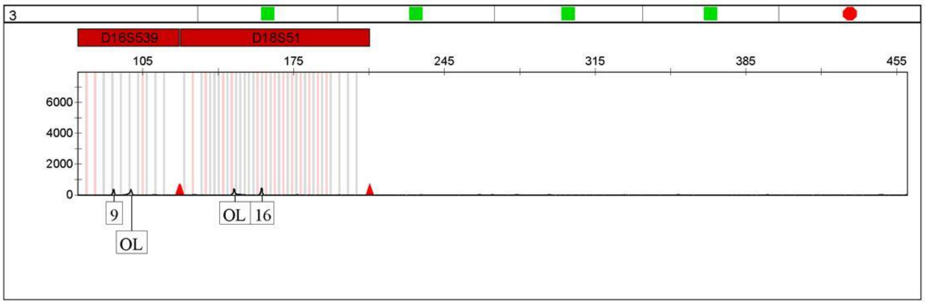

GeneMapper® ID-X 1.1

Project: noura_breast_cancer

Sample Name	SOS	SQ	SSPK	MIX	CGQ
3					

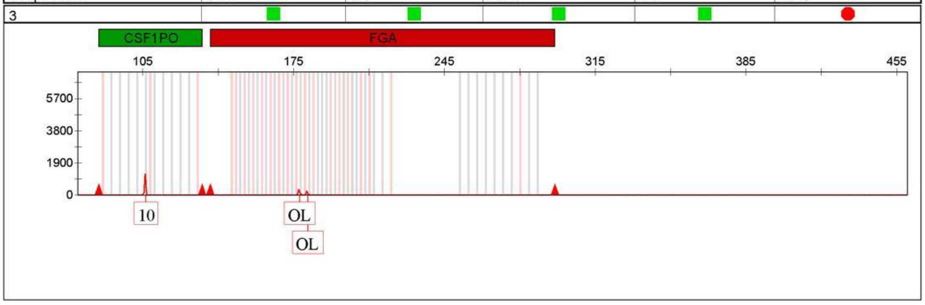

Sample Name	SOS	SQ	SSPK	MIX	CGQ
11					

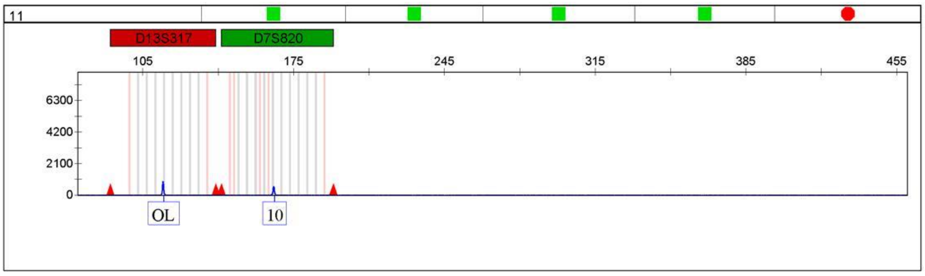

Sample Name	SOS	SQ	SSPK	MIX	CGQ
11					

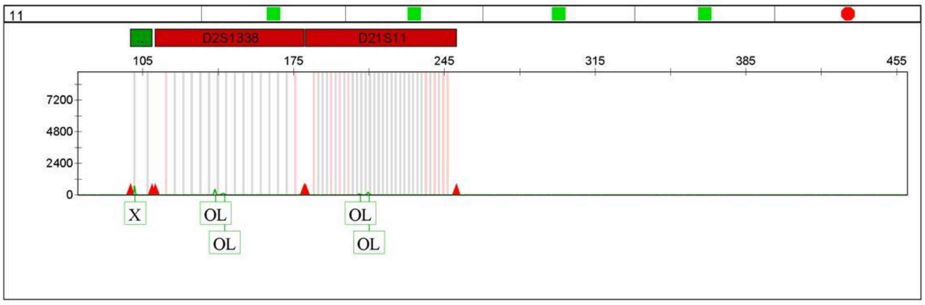

Sample Name	SOS	SQ	SSPK	MIX	CGQ
11					

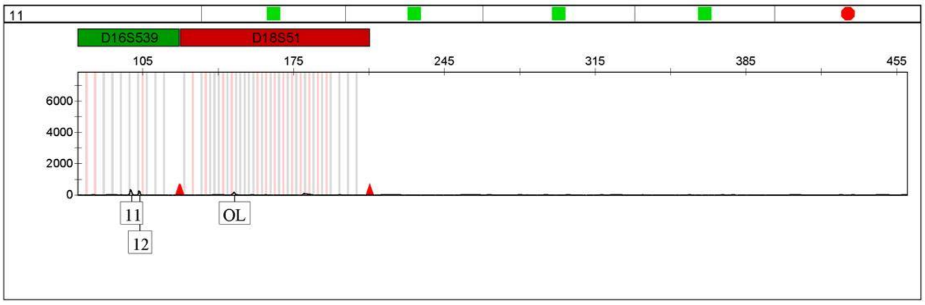

Sample Name	SOS	SQ	SSPK	MIX	CGQ
11					

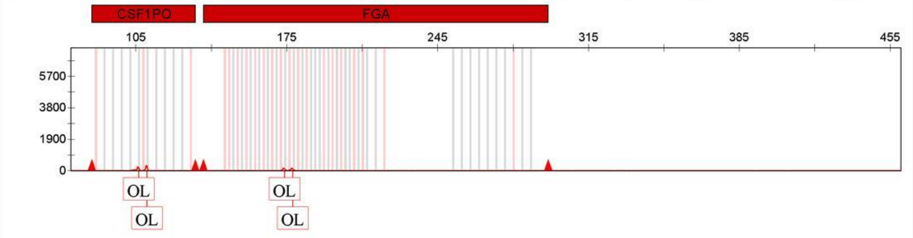

Sample Name	SOS	SQ	SSPK	MIX	CGQ
19					

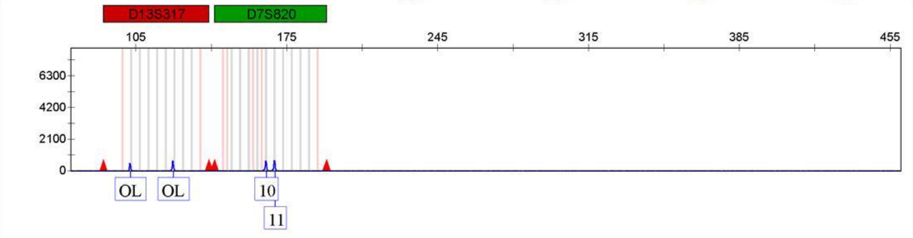

Sample Name	SOS	SQ	SSPK	MIX	CGQ
19					

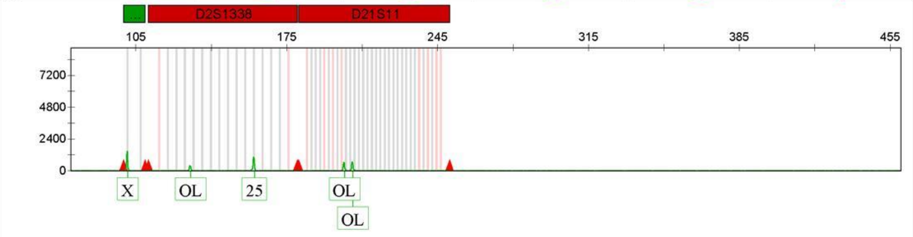

Sample Name	SOS	SQ	SSPK	MIX	CGQ
19					

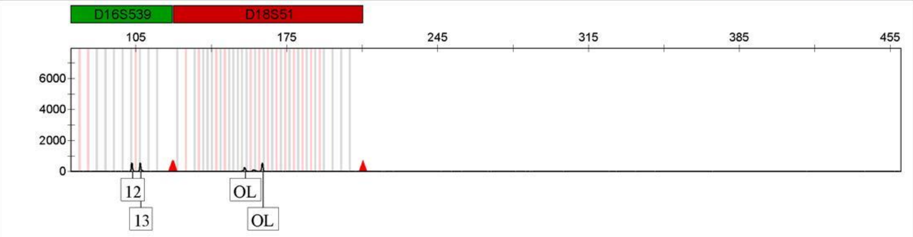

Sample Name	SOS	SQ	SSPK	MIX	CGQ
19					

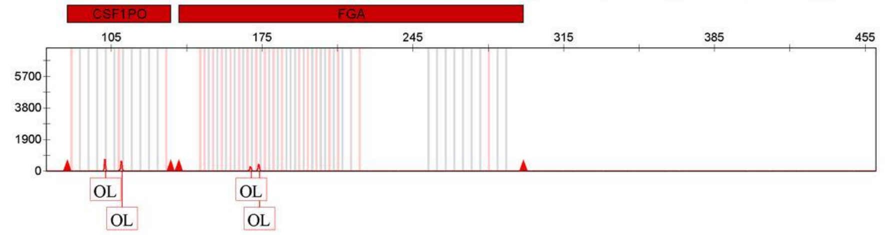

Sample Name	SOS	SQ	SSPK	MIX	CGQ
28					

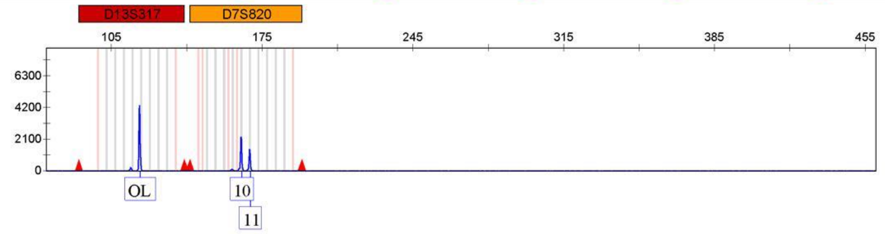

Sample Name	SOS	SQ	SSPK	MIX	CGQ
28					

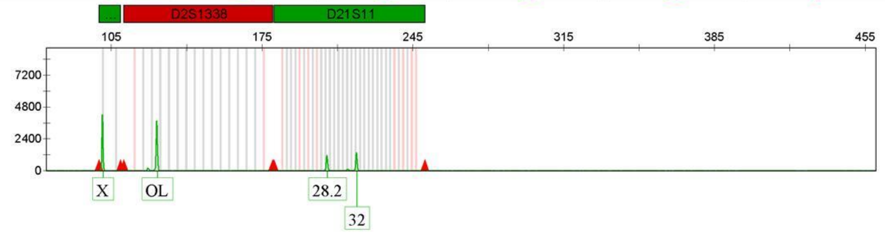

Sample Name	SOS	SQ	SSPK	MIX	CGQ
28					

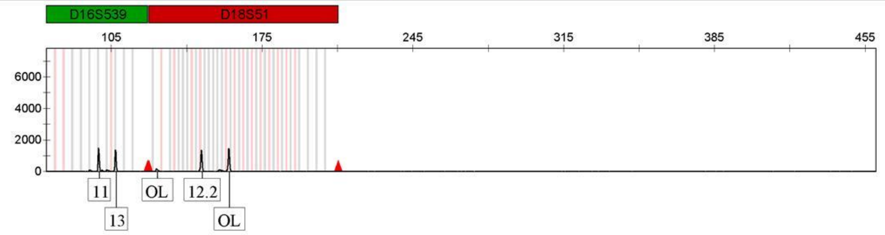

Sample Name	SOS	SQ	SSPK	MIX	CGQ
28					

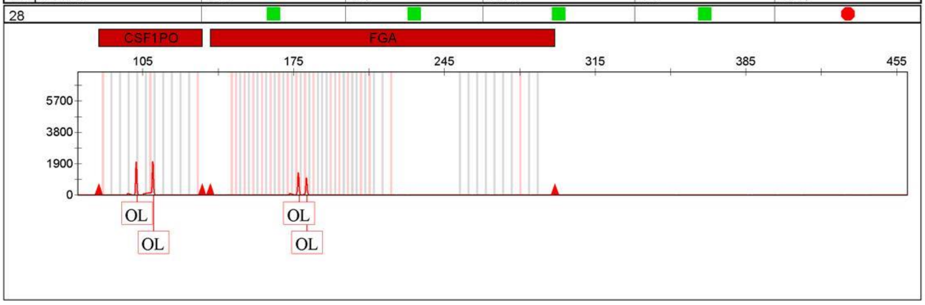

Sample Name	SOS	SQ	SSPK	MIX	CGQ
B16					

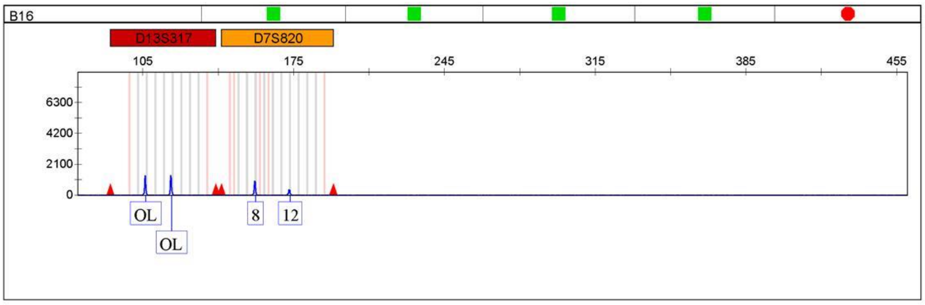

Sample Name	SOS	SQ	SSPK	MIX	CGQ
B16					

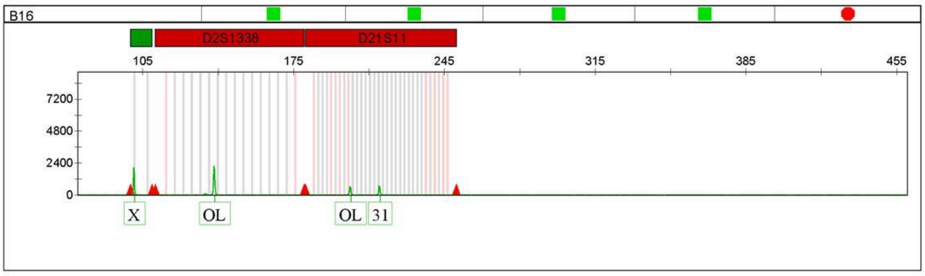

Sample Name	SOS	SQ	SSPK	MIX	CGQ
B16					

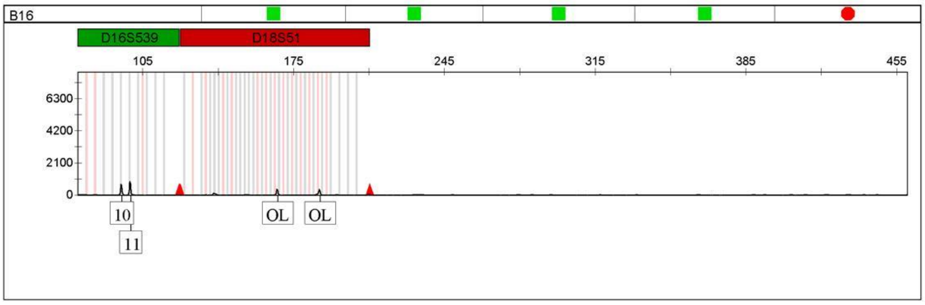

Sample Name	SOS	SQ	SSPK	MIX	CGQ
B16					

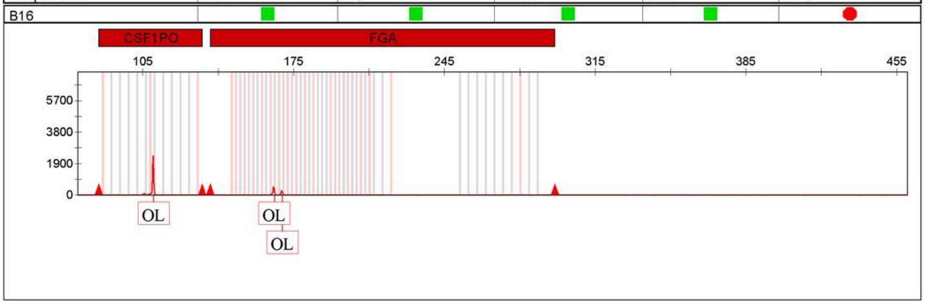

Sample Name	SOS	SQ	SSPK	MIX	CGQ
A9					

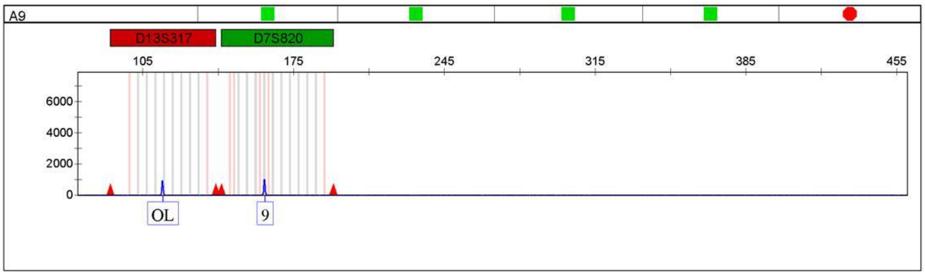

Sample Name	SOS	SQ	SSPK	MIX	CGQ
A9					

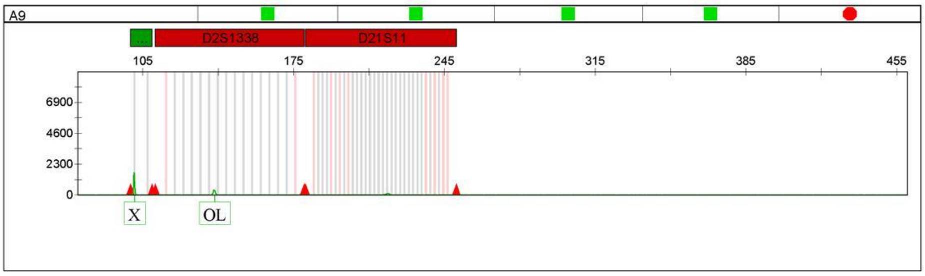

Sample Name	SOS	SQ	SSPK	MIX	CGQ
A9					

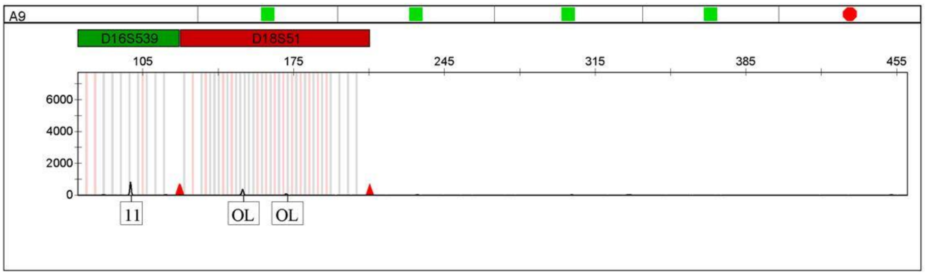

Sample Name	SOS	SQ	SSPK	MIX	CGQ
A9					

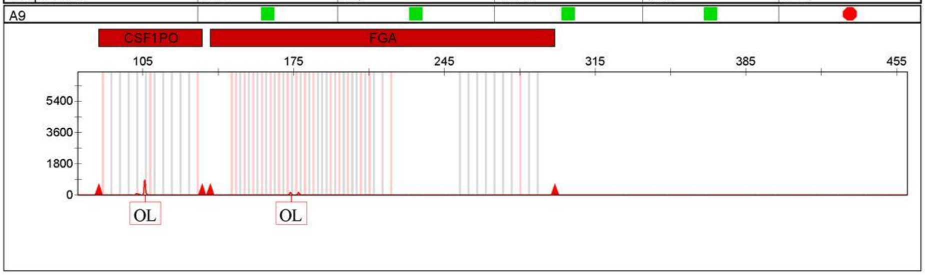

Sample Name	SOS	SQ	SSPK	MIX	CGQ
A54					

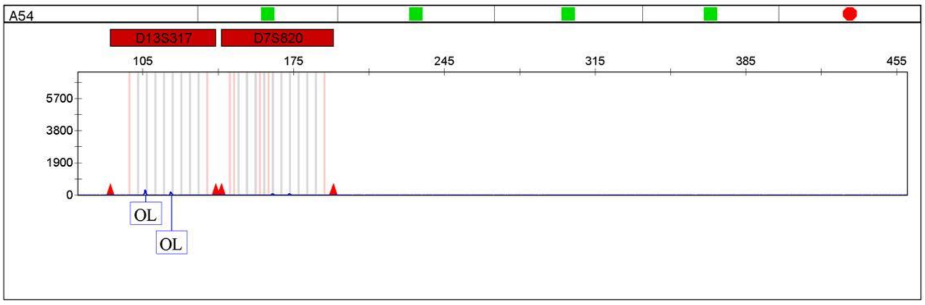

Sample Name	SOS	SQ	SSPK	MIX	CGQ
A54					

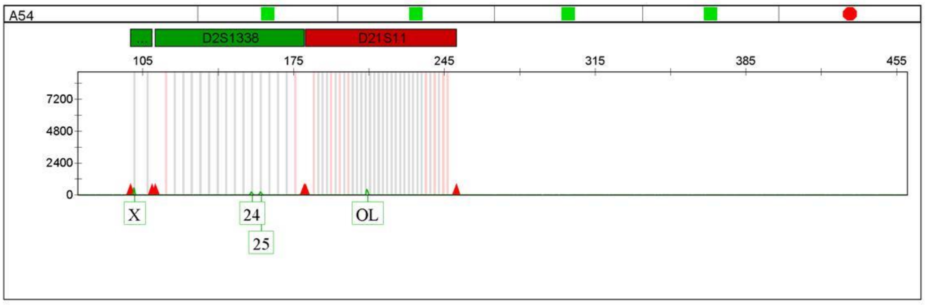

Sample Name	SOS	SQ	SSPK	MIX	CGQ
A54					

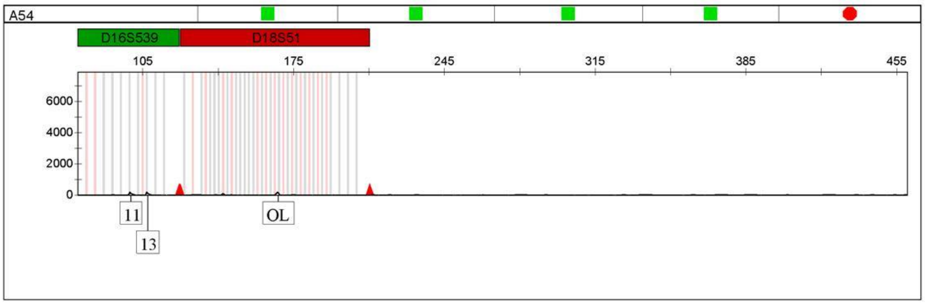

Sample Name	SOS	SQ	SSPK	MIX	CGQ
A54					

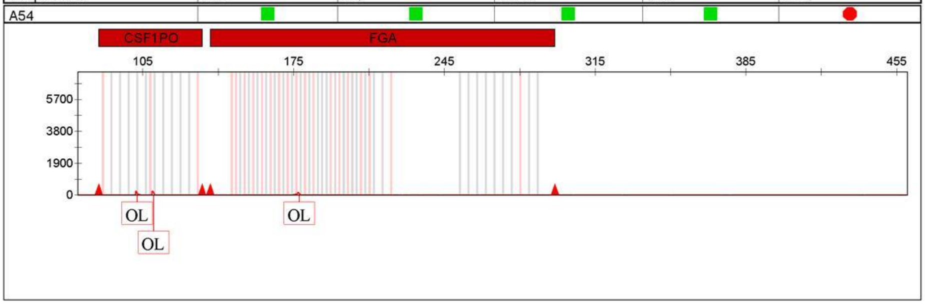

A82					

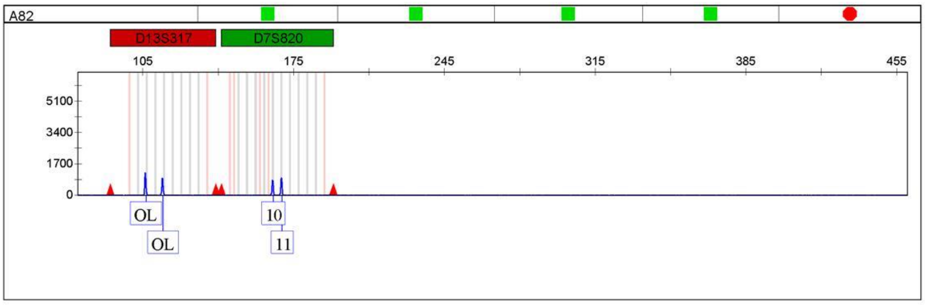

A82					

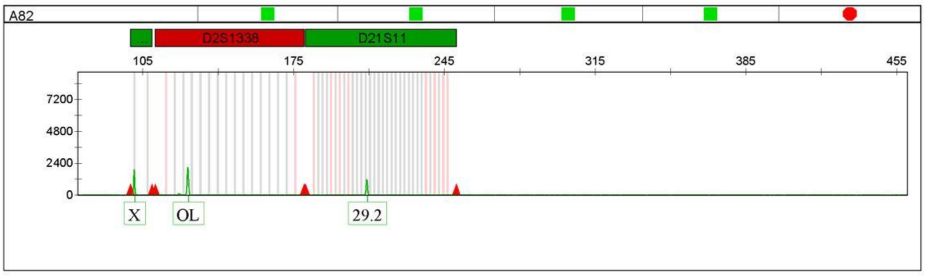

A82					

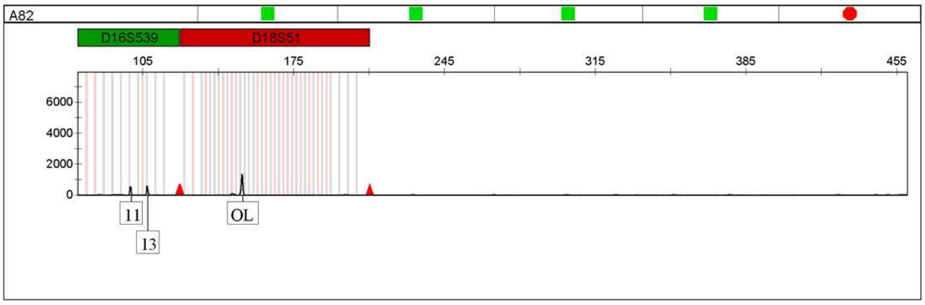

Sample Name	SOS	SQ	SSPK	MIX	CGQ
A82					

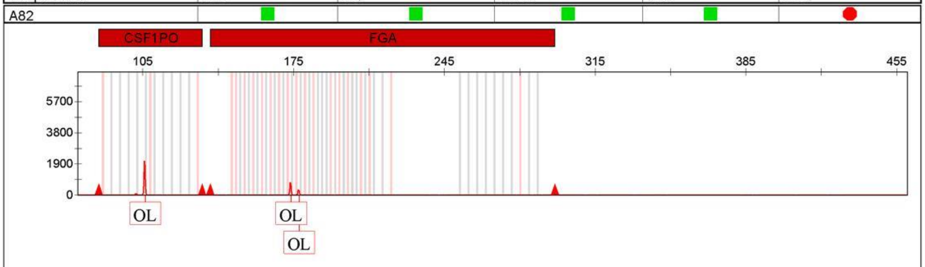

Sample Name	SOS	SQ	SSPK	MIX	CGQ
B11					

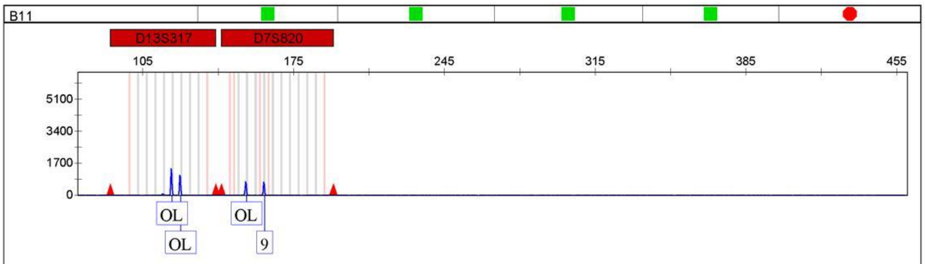

Sample Name	SOS	SQ	SSPK	MIX	CGQ
B11					

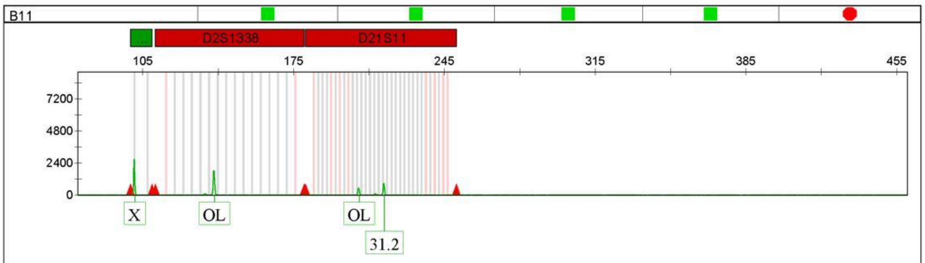

Sample Name	SOS	SQ	SSPK	MIX	CGQ
B11					

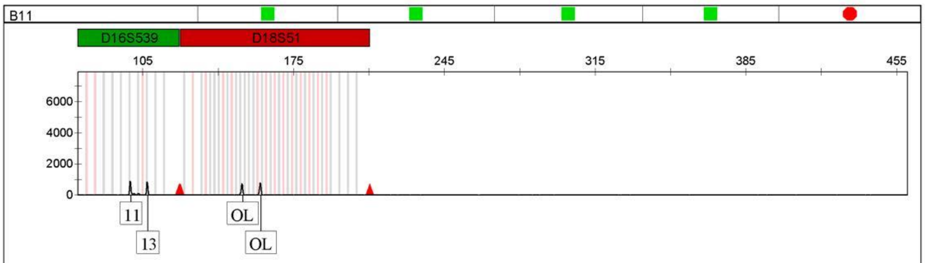

Sample Name	SOS	SQ	SSPK	MIX	CGQ
B11					

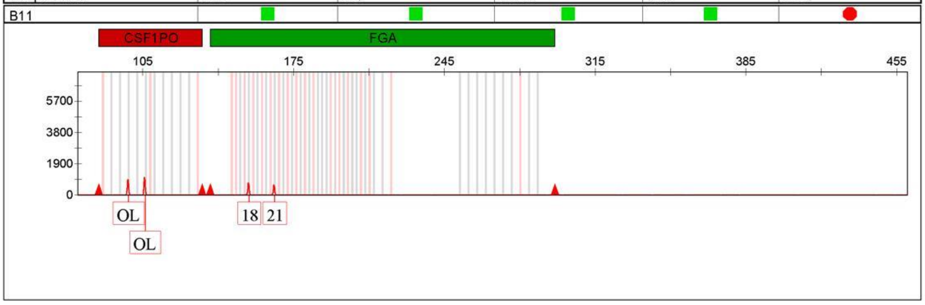

Sample Name	SOS	SQ	SSPK	MIX	CGQ
B41					

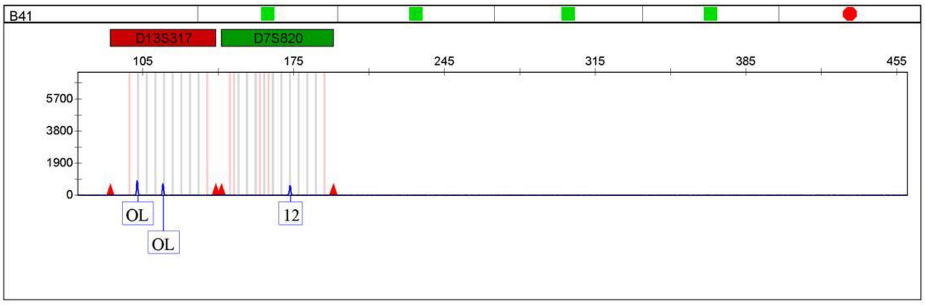

Sample Name	SOS	SQ	SSPK	MIX	CGQ
B41					

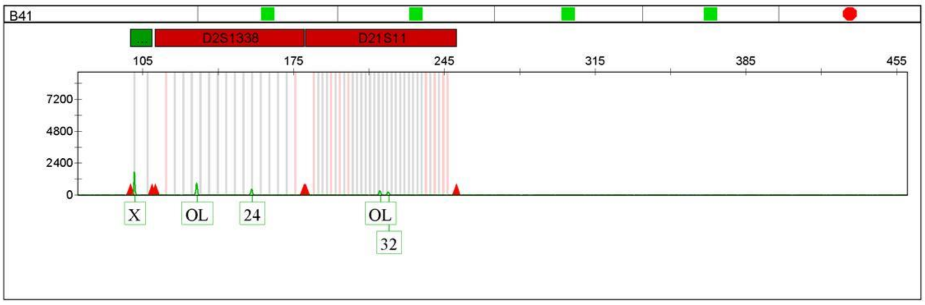

Sample Name	SOS	SQ	SSPK	MIX	CGQ
B41					

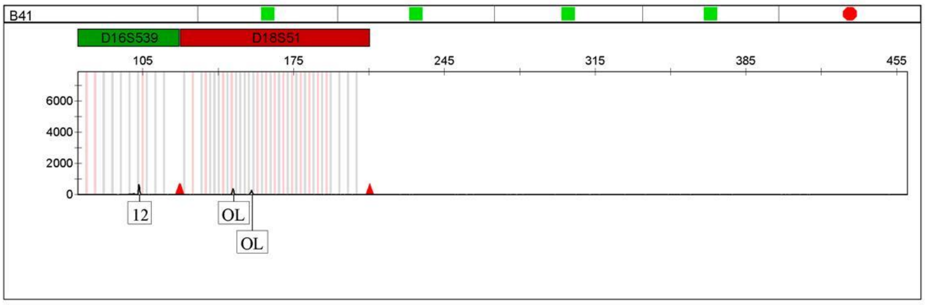

Applied Biosystems
GeneMapper® ID-X 1.1

Project: noura_breast_cancer

Sample Name	SOS	SQ	SSPK	MIX	CGQ
B41					

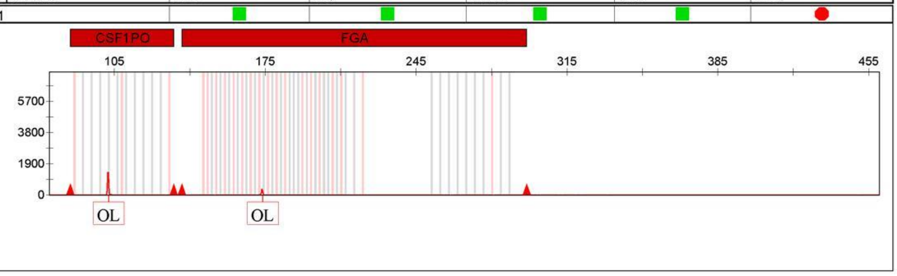

8					

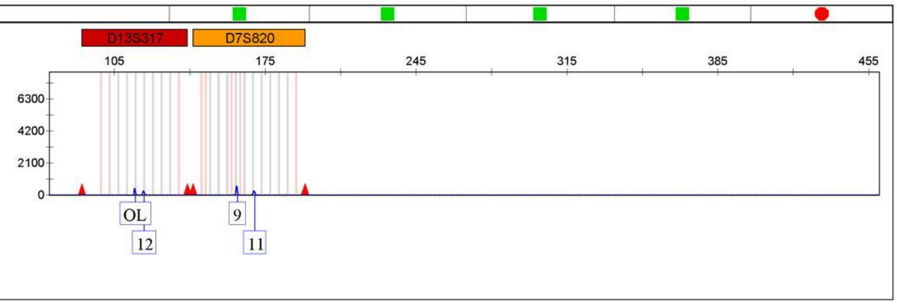

8					

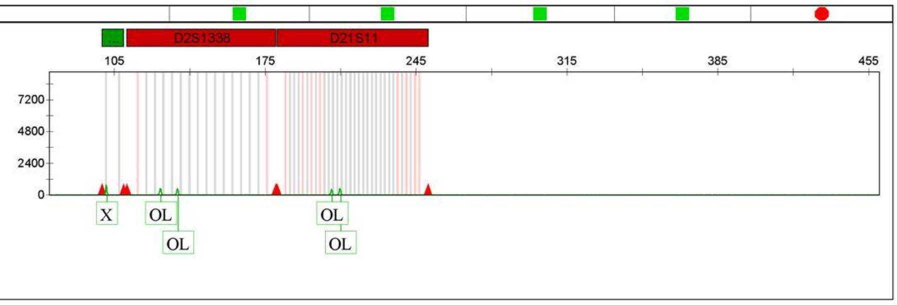

8					

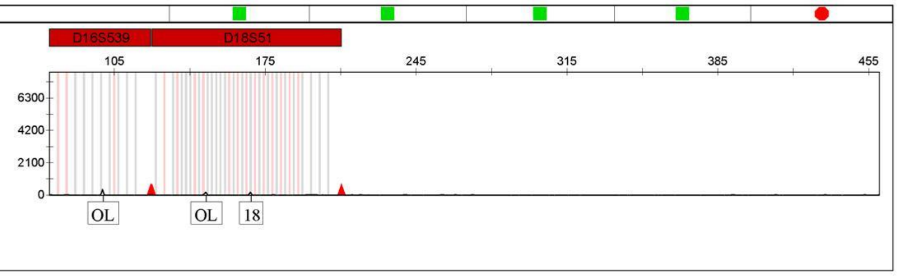

Applied Biosystems
GeneMapper® ID-X 1.1

Project: noura_breast_cancer

Sample Name	SOS	SQ	SSPK	MIX	CGQ
8					

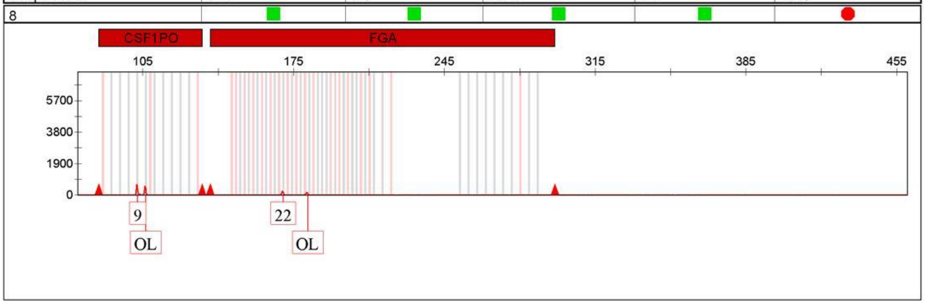

16					

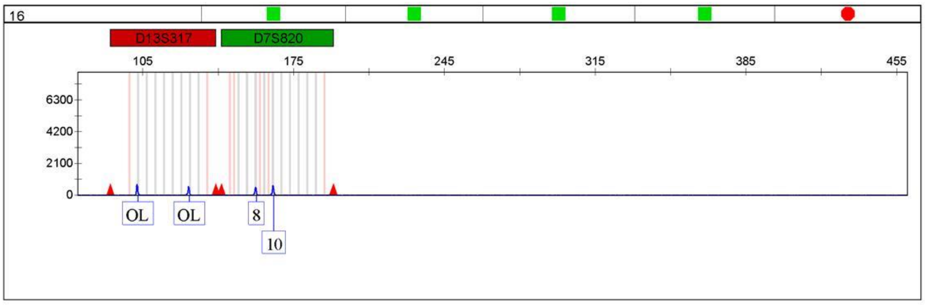

16					

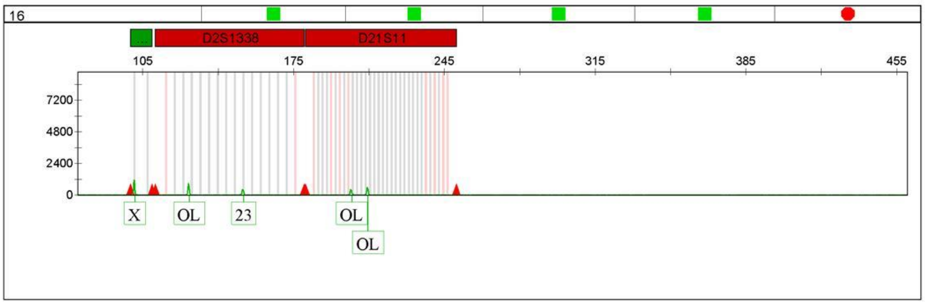

16					

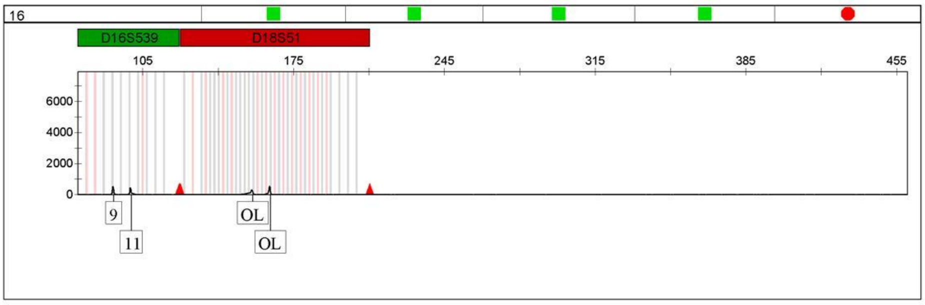

Sample Name	SOS	SQ	SSPK	MIX	CGQ
16					

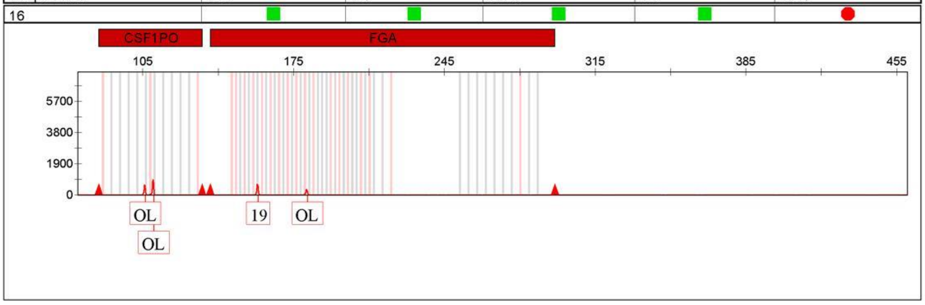

24					

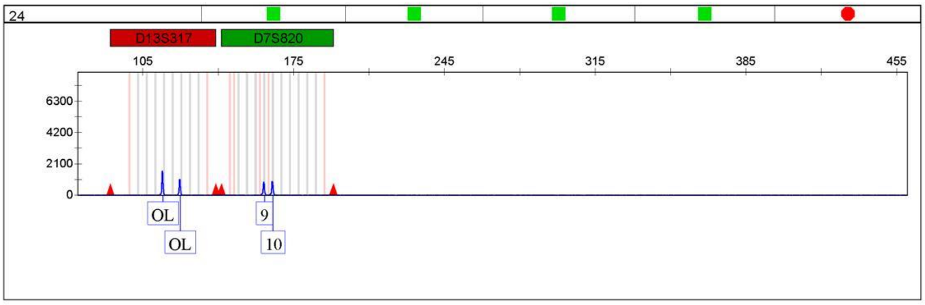

24					

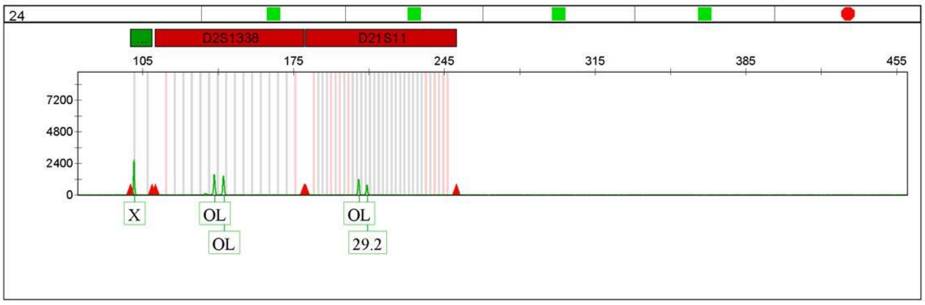

24					

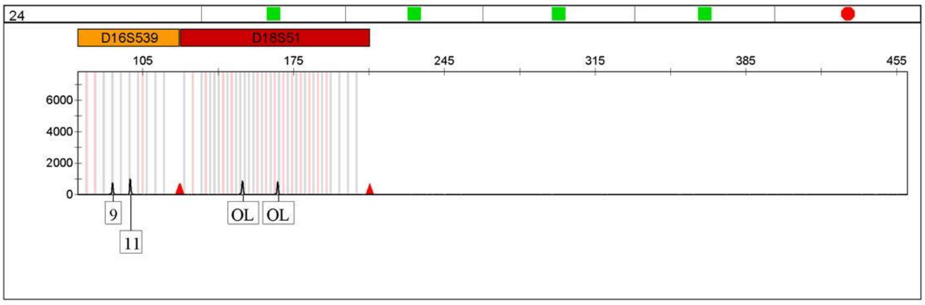

Sample Name	SOS	SQ	SSPK	MIX	CGQ
24					

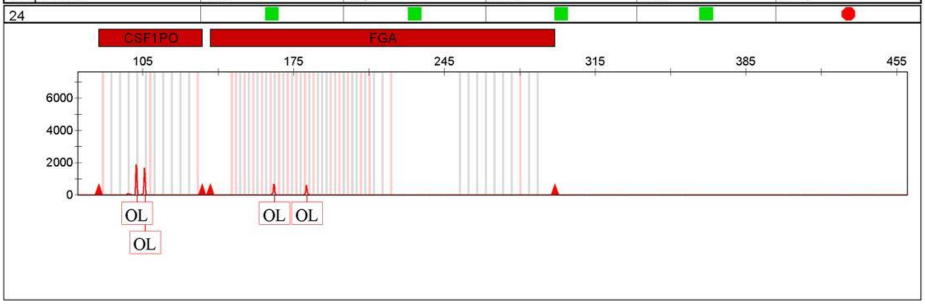

Sample Name	SOS	SQ	SSPK	MIX	CGQ
5B					

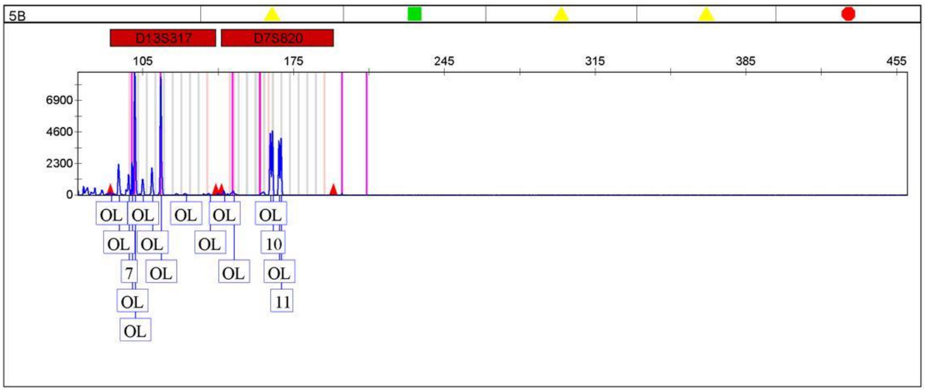

Sample Name	SOS	SQ	SSPK	MIX	CGQ
5B					

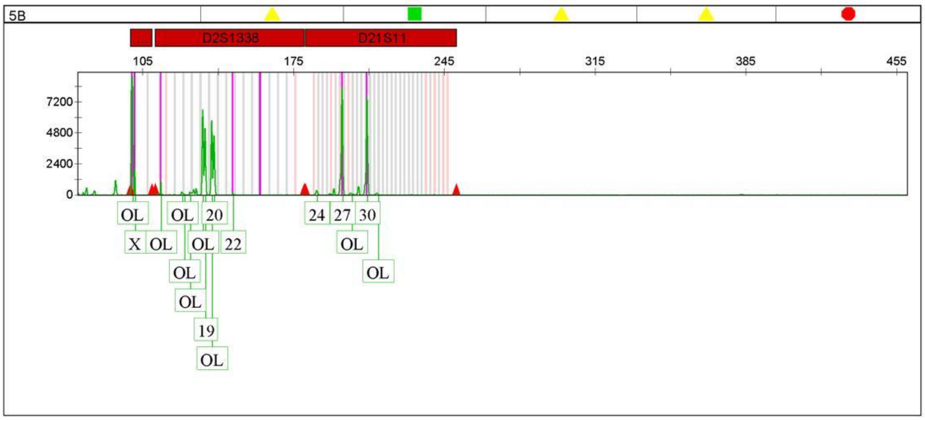

Sample Name	SOS	SQ	SSPK	MIX	CGQ
5B					

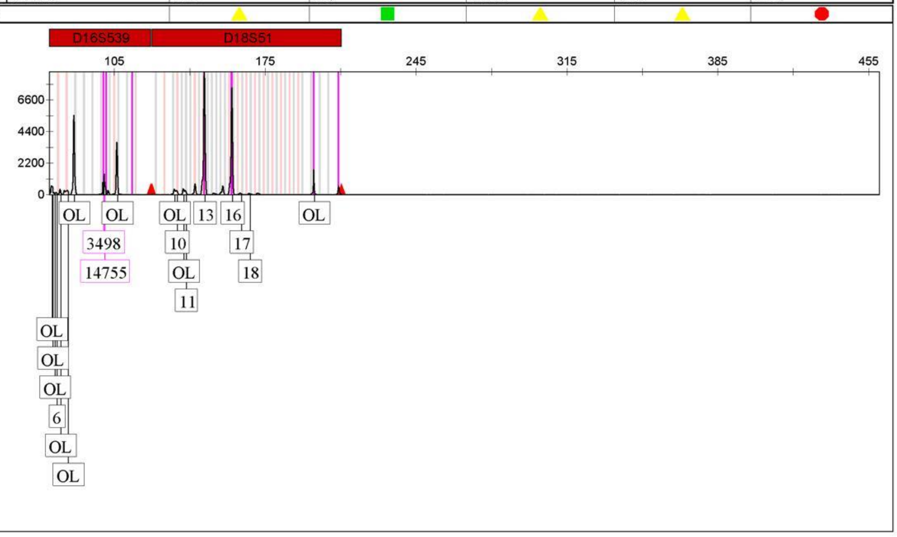

Sample Name	SOS	SQ	SSPK	MIX	CGQ
5B					

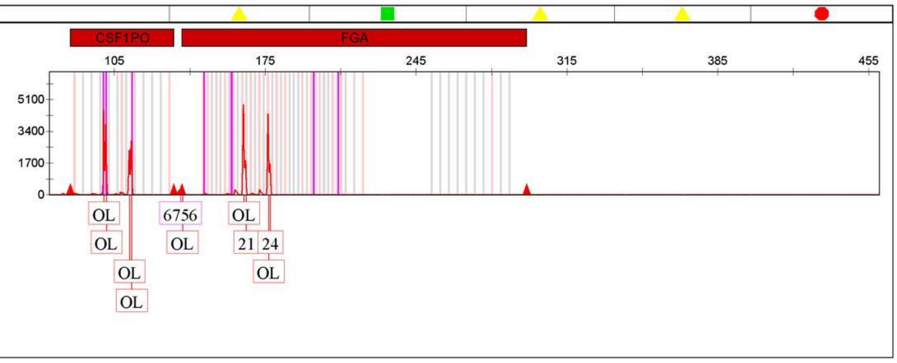

Sample Name	SOS	SQ	SSPK	MIX	CGQ
A71					

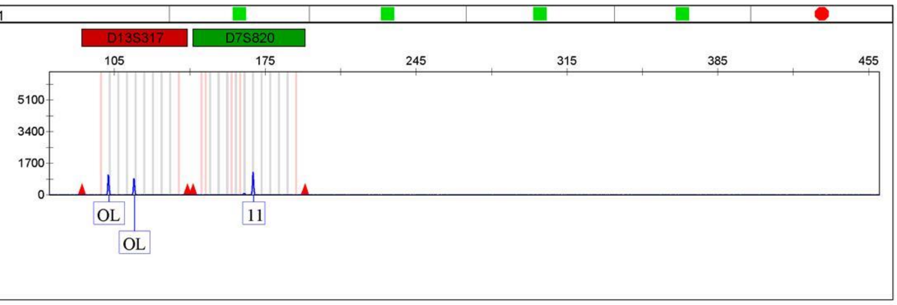

Sample Name	SOS	SQ	SSPK	MIX	CGQ
A71					

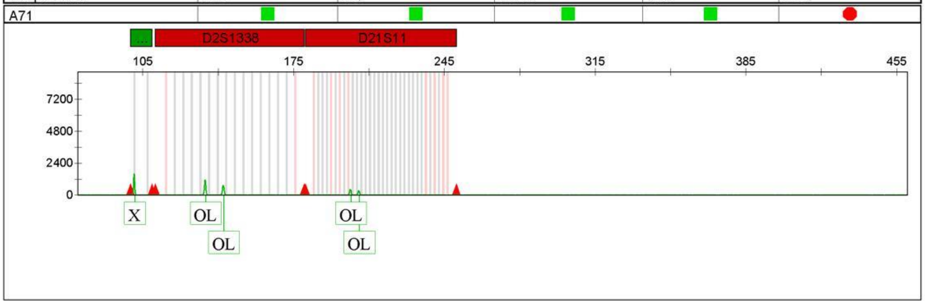

A71					

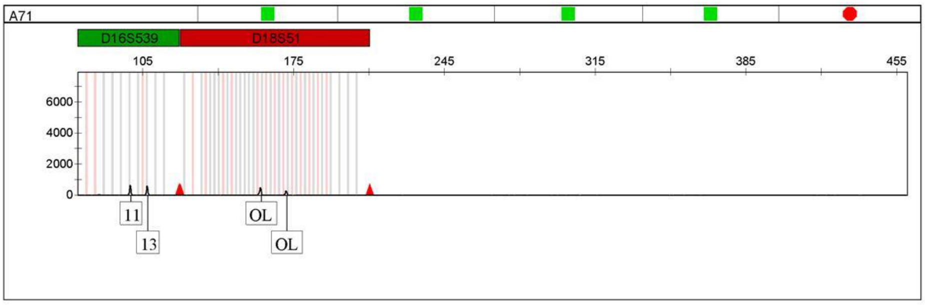

A71					

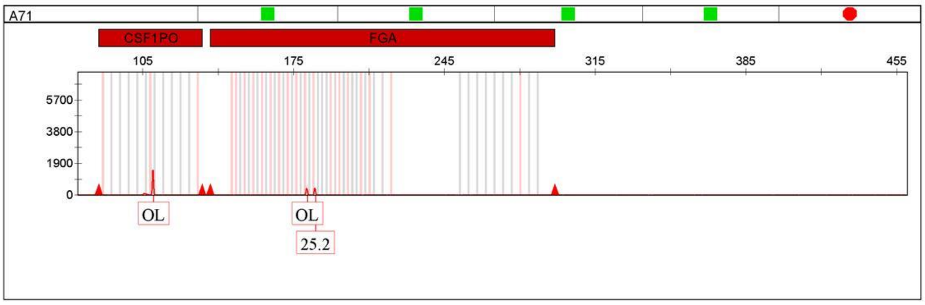

Sample Name	SOS	SQ	SSPK	MIX	CGQ
A93					

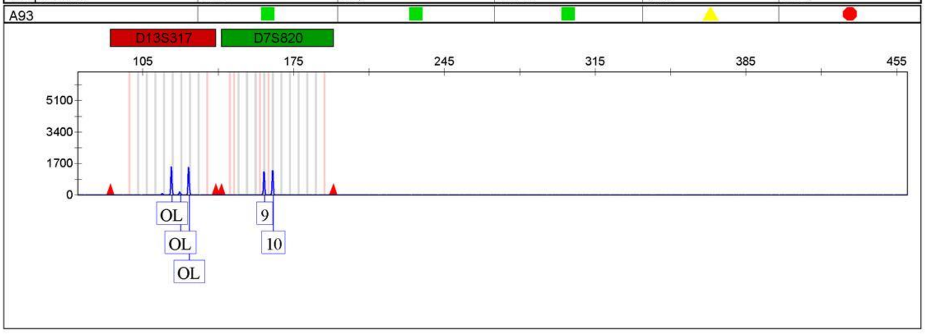

A93					

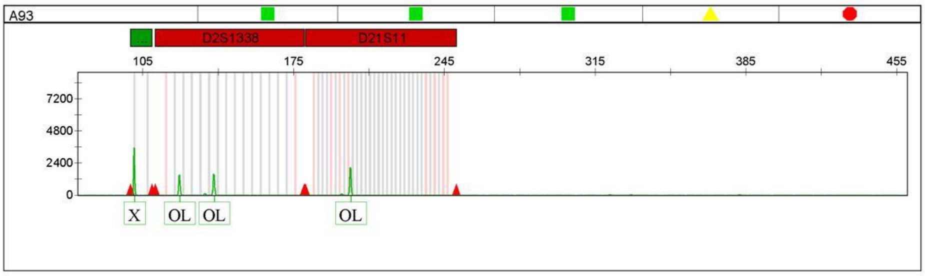

A93					

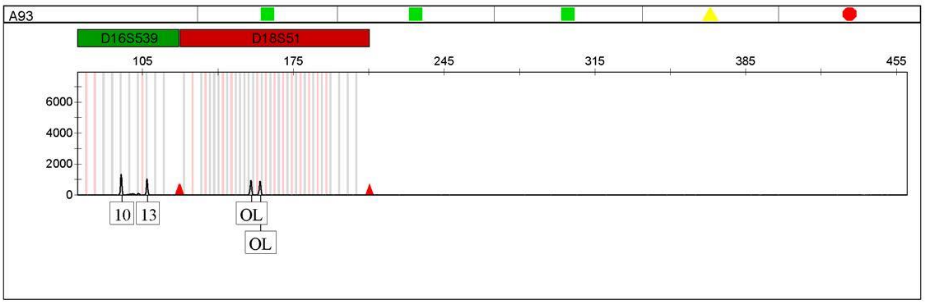

Sample Name	SOS	SQ	SSPK	MIX	CGQ
A93					

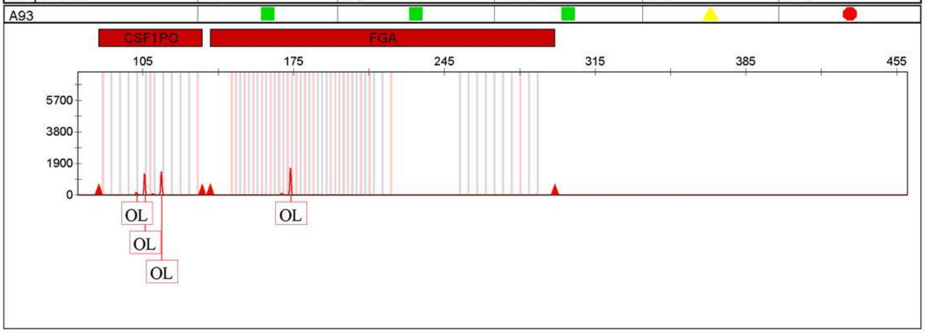

Sample Name	SOS	SQ	SSPK	MIX	CGQ
B24					

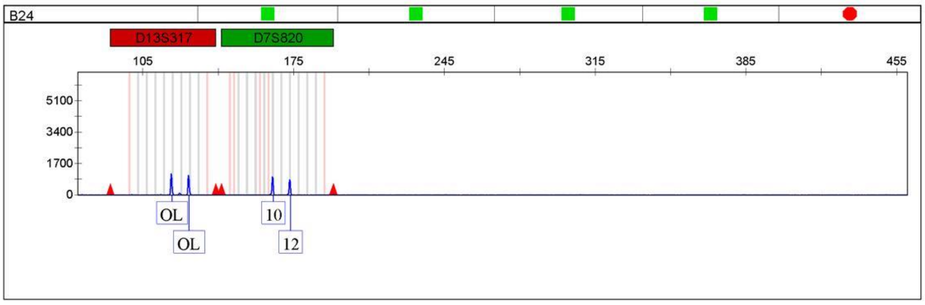

Sample Name	SOS	SQ	SSPK	MIX	CGQ
B24					

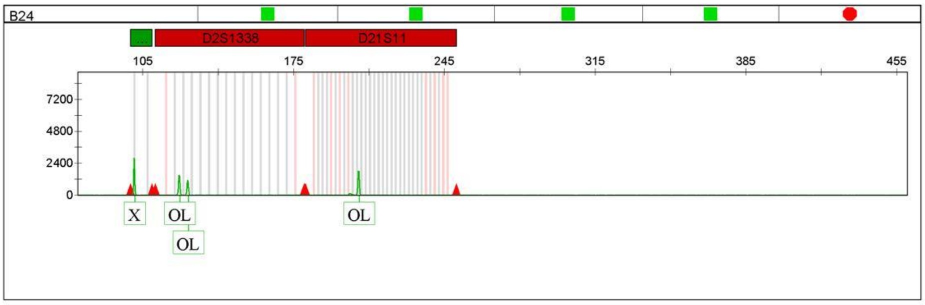

Sample Name	SOS	SQ	SSPK	MIX	CGQ
B24					

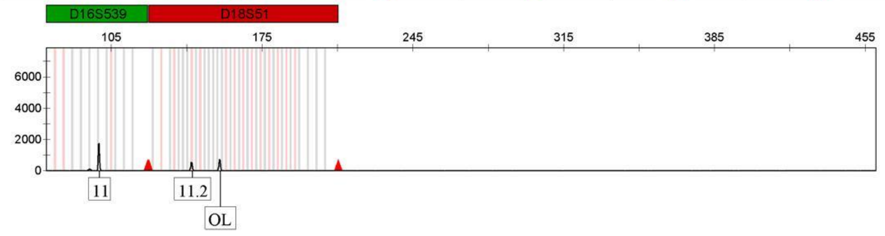

B24					

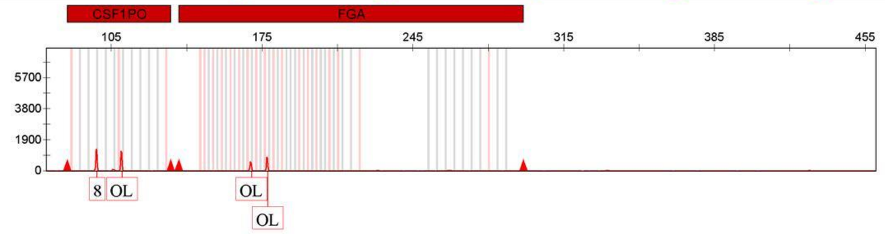

B37					

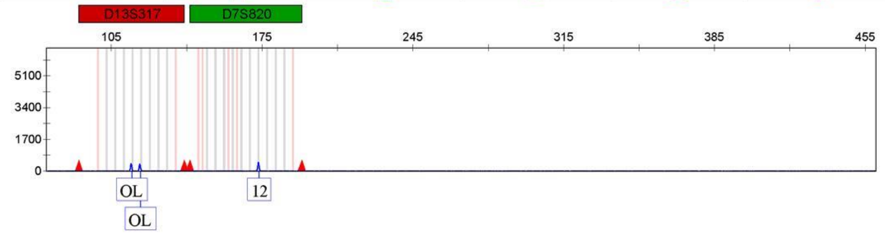

B37					

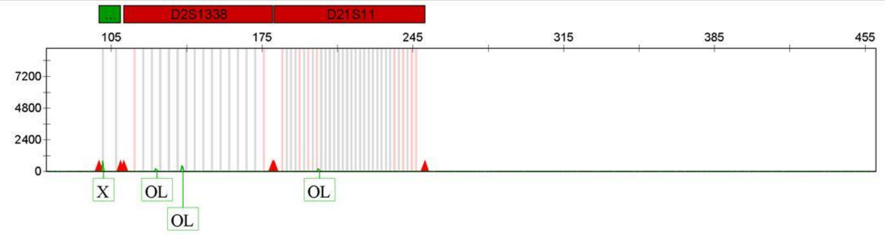

Sample Name	SOS	SQ	SSPK	MIX	CGQ
B37					

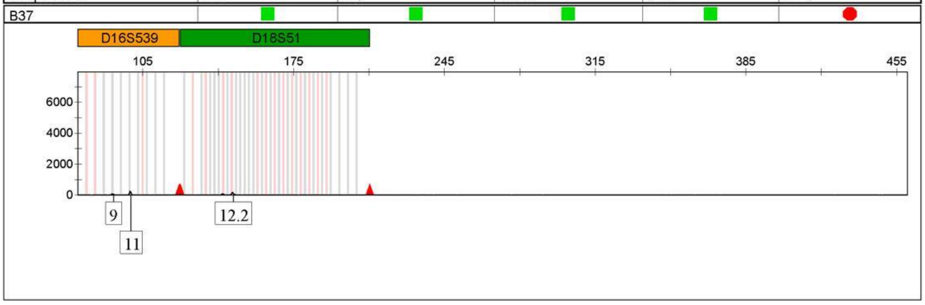

Sample Name	SOS	SQ	SSPK	MIX	CGQ
B37					

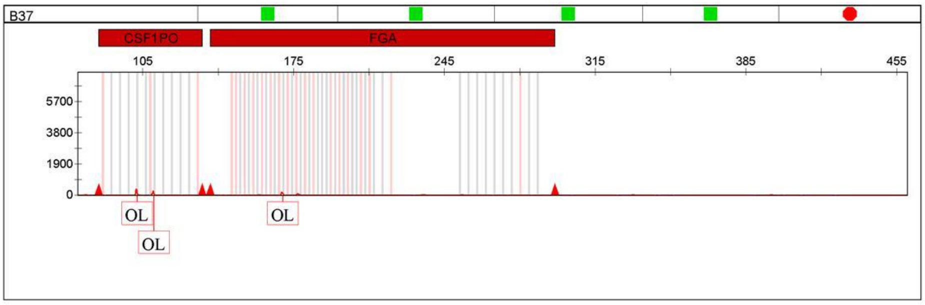

Sample Name	SOS	SQ	SSPK	MIX	CGQ
4					

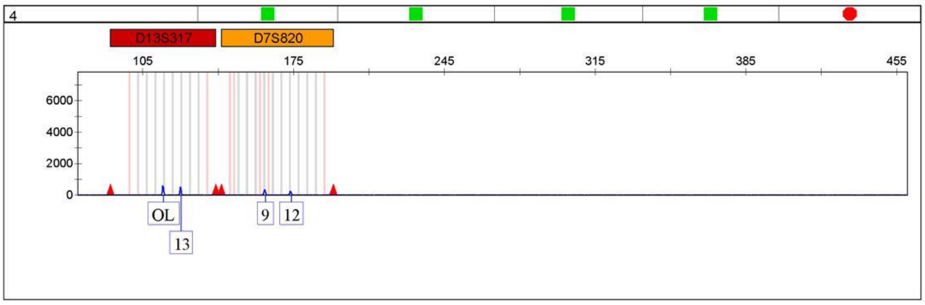

Sample Name	SOS	SQ	SSPK	MIX	CGQ
4					

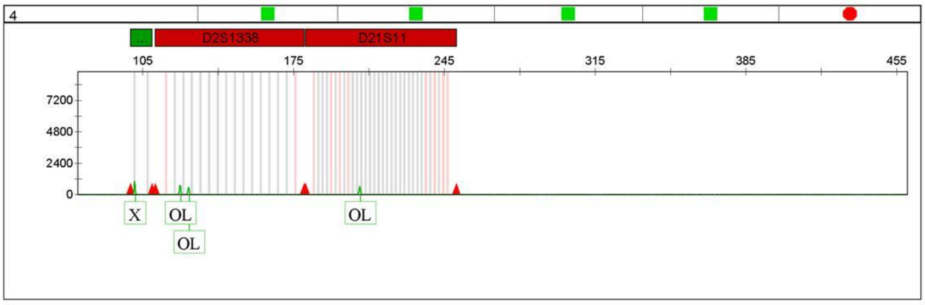

Sample Name	SOS	SQ	SSPK	MIX	CGQ
4					

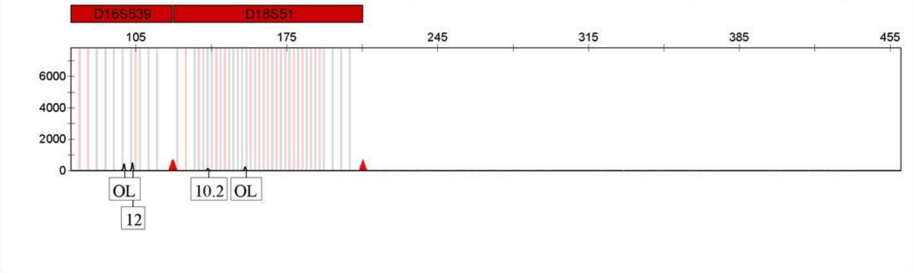

Sample Name	SOS	SQ	SSPK	MIX	CGQ
4					

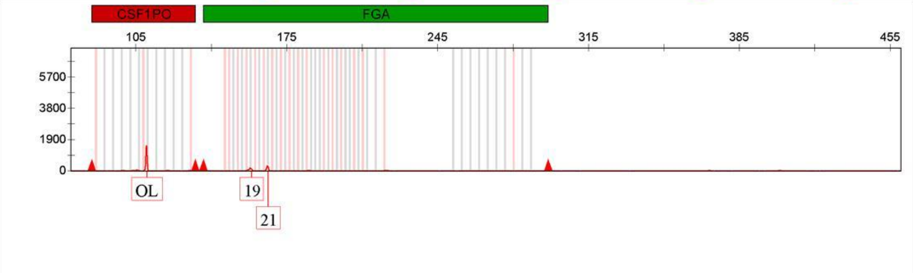

Sample Name	SOS	SQ	SSPK	MIX	CGQ
12					

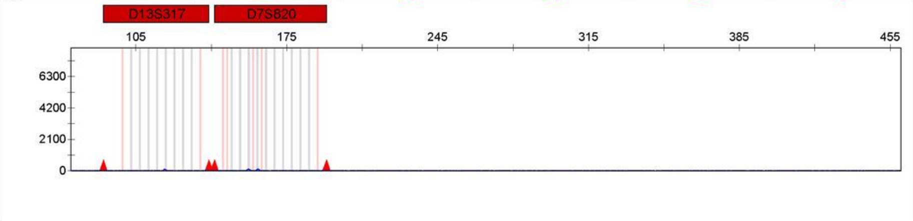

Sample Name	SOS	SQ	SSPK	MIX	CGQ
12					

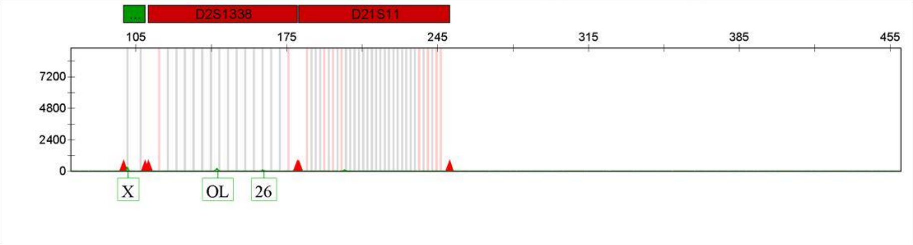

Sample Name	SOS	SQ	SSPK	MIX	CGQ
12					

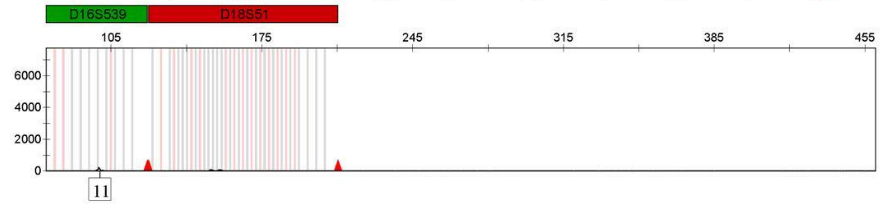

Sample Name	SOS	SQ	SSPK	MIX	CGQ
12					

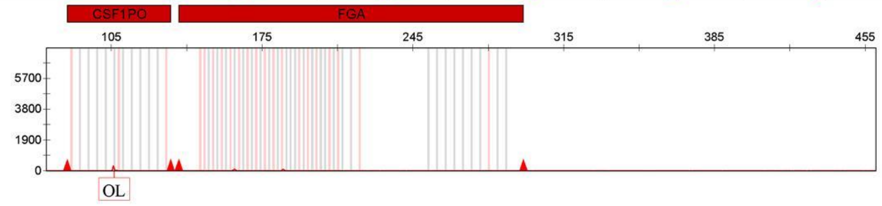

Sample Name	SOS	SQ	SSPK	MIX	CGQ
20					

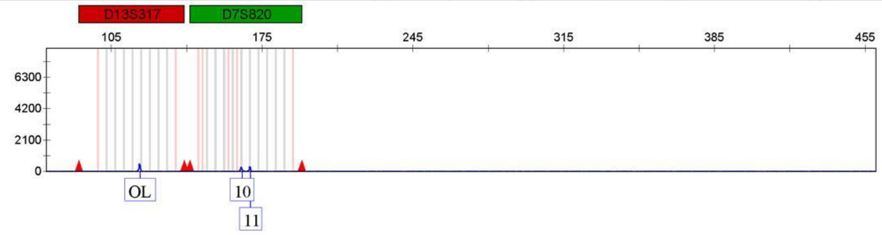

Sample Name	SOS	SQ	SSPK	MIX	CGQ
20					

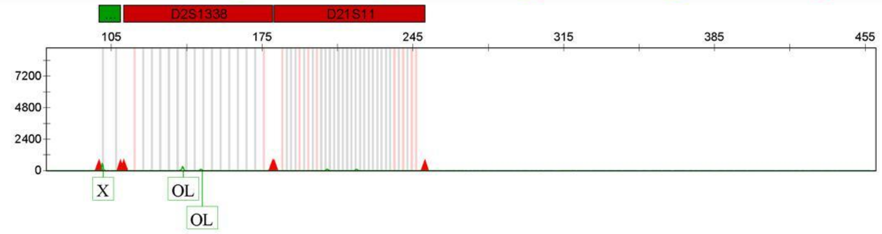

Sample Name	SOS	SQ	SSPK	MIX	CGQ
20					

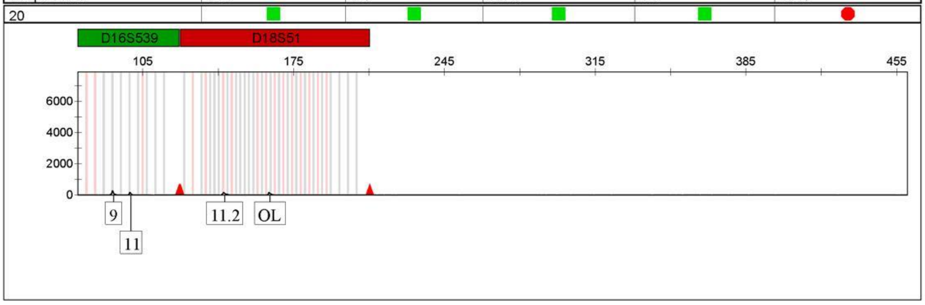

Sample Name	SOS	SQ	SSPK	MIX	CGQ
20					

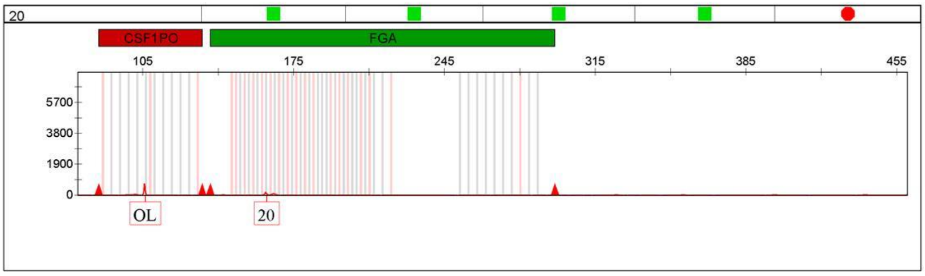

Sample Name	SOS	SQ	SSPK	MIX	CGQ
29					

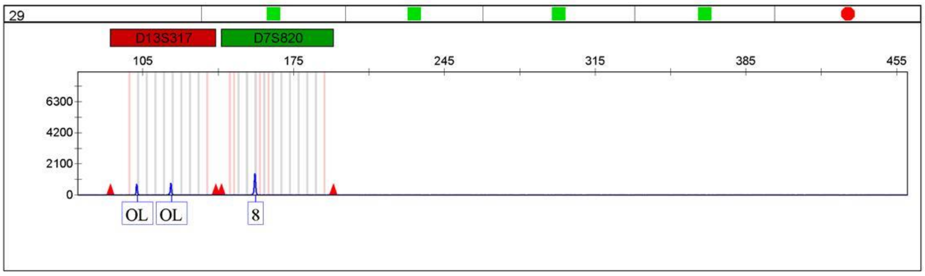

Sample Name	SOS	SQ	SSPK	MIX	CGQ
29					

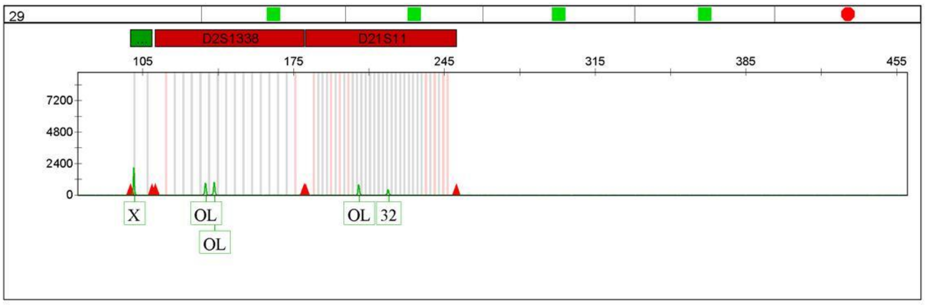

Sample Name	SOS	SQ	SSPK	MIX	CGQ
29					

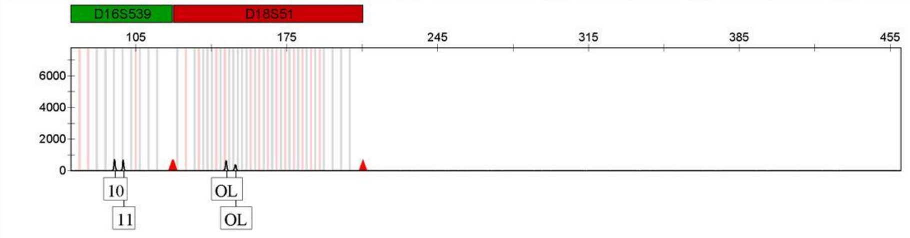

Sample Name	SOS	SQ	SSPK	MIX	CGQ
29					

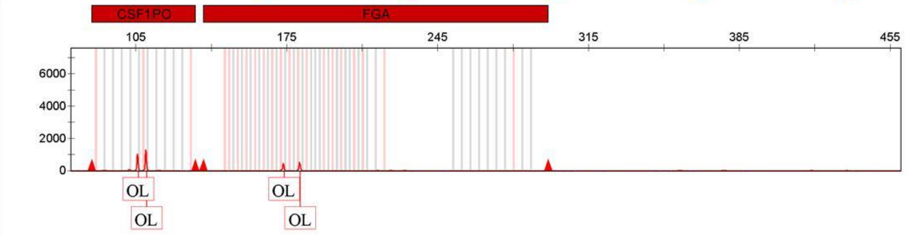

Sample Name	SOS	SQ	SSPK	MIX	CGQ
1B					

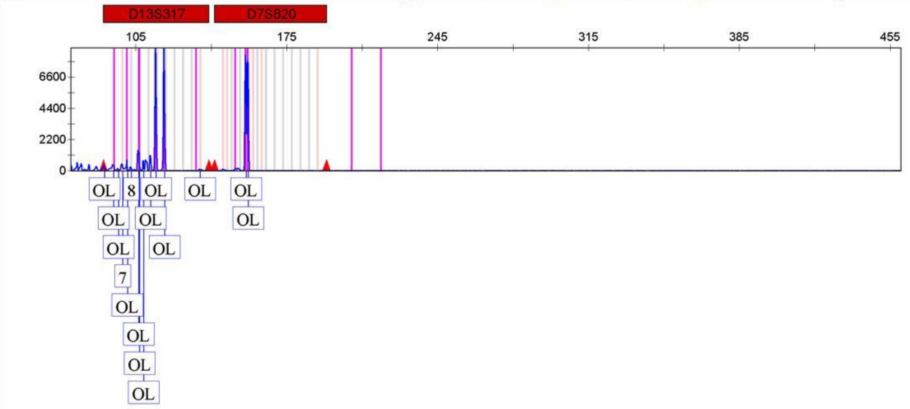

Sample Name	SOS	SQ	SSPK	MIX	CGQ
1B					

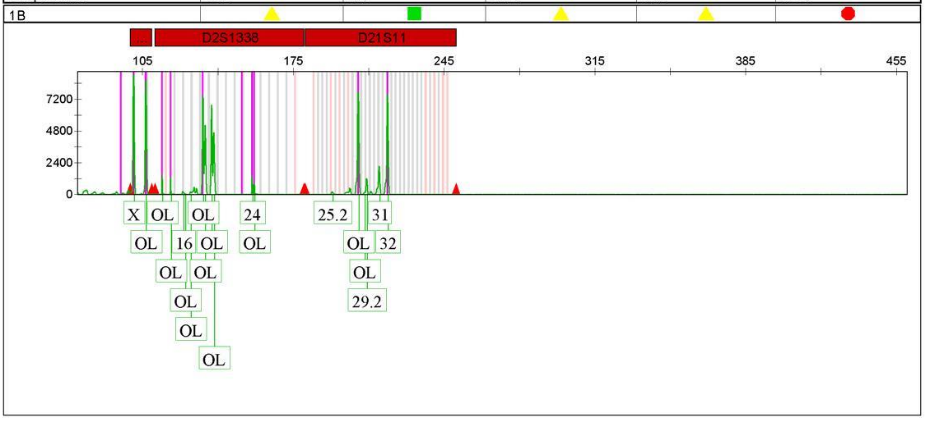

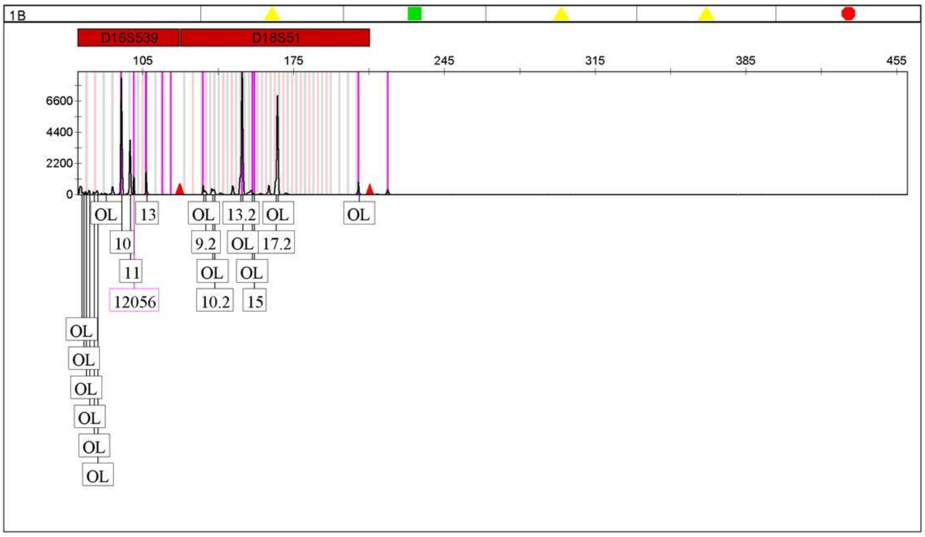

Sample Name	SOS	SQ	SSPK	MIX	CGQ
1B					

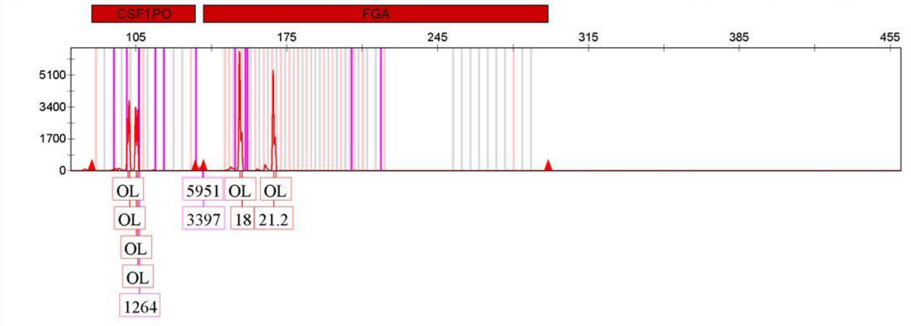

A15					

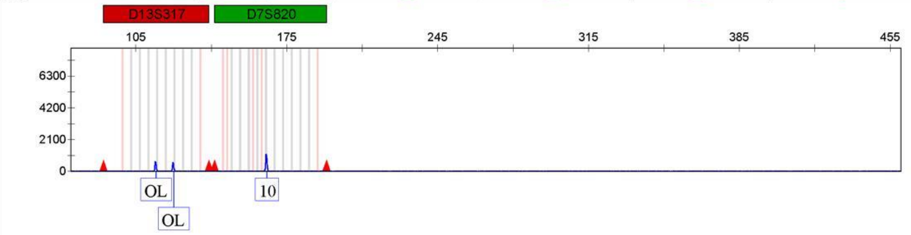

A15					

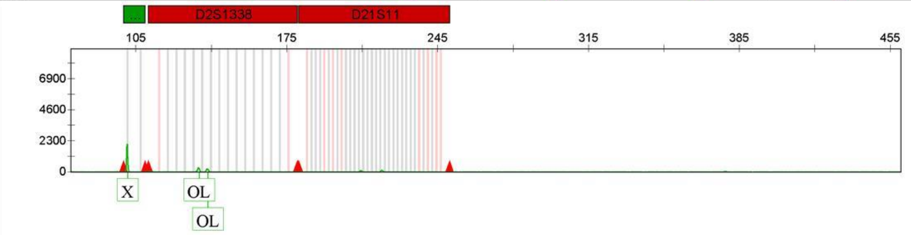

Sample Name	SOS	SQ	SSPK	MIX	CGQ
A15					

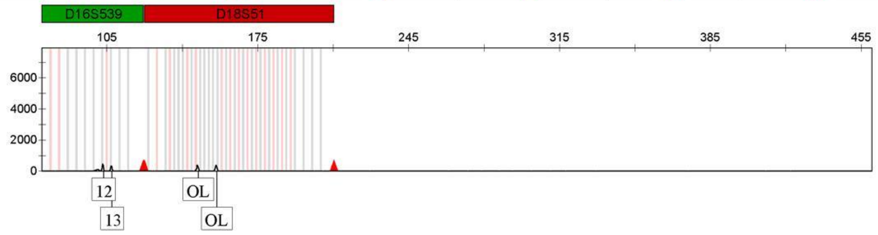

A15					

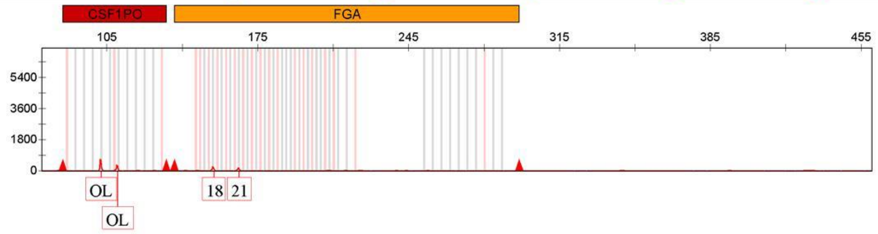

A55					

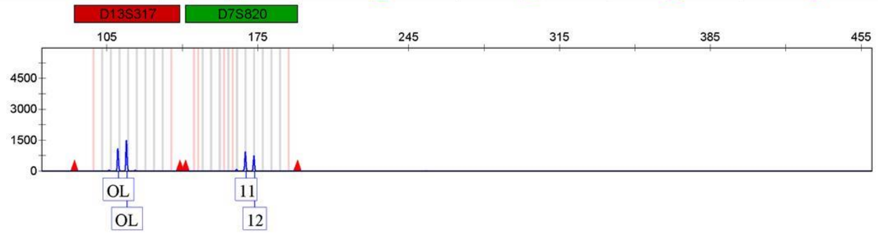

A55					

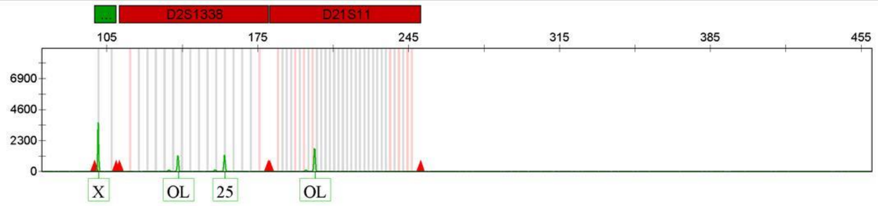

Sample Name	SOS	SQ	SSPK	MIX	CGQ
A55					

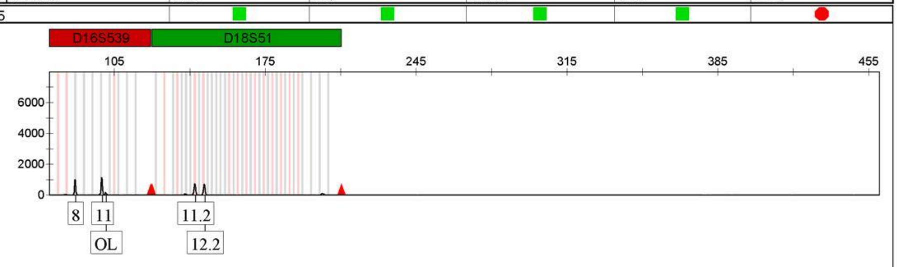

Sample Name	SOS	SQ	SSPK	MIX	CGQ
A55					

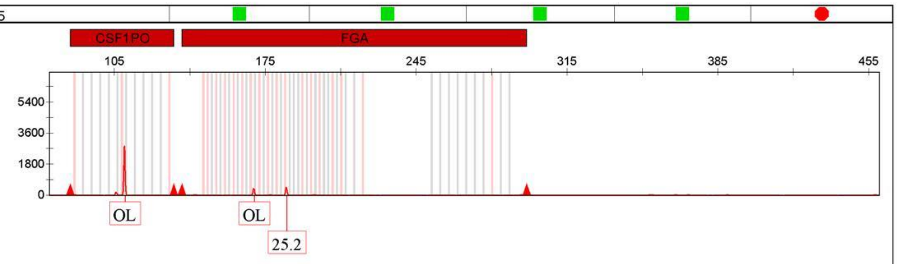

Sample Name	SOS	SQ	SSPK	MIX	CGQ
A88					

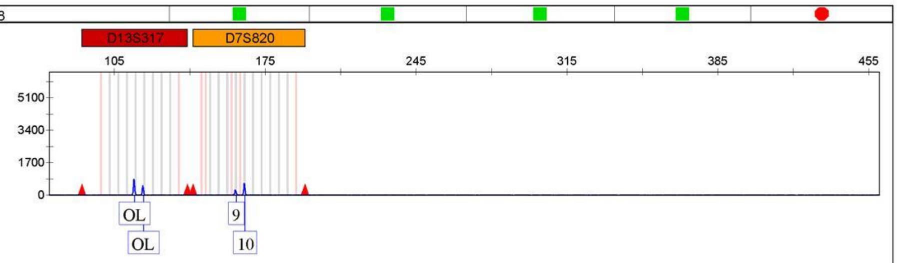

Sample Name	SOS	SQ	SSPK	MIX	CGQ
A88					

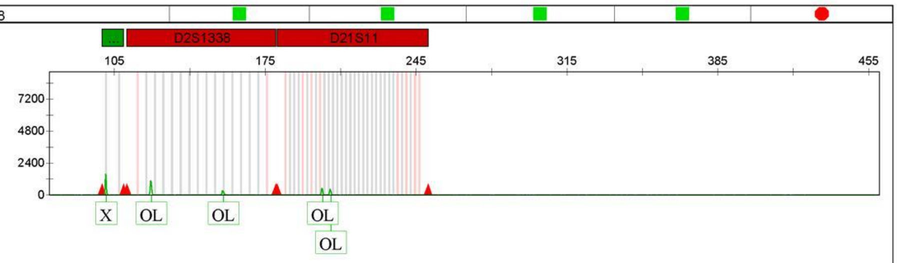

Sample Name	SOS	SQ	SSPK	MIX	CGQ
A88					

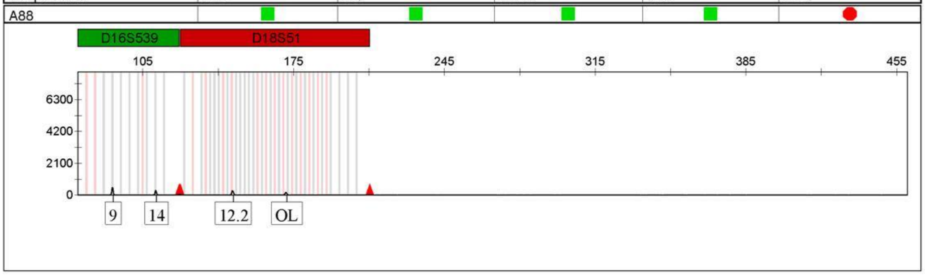

Sample Name	SOS	SQ	SSPK	MIX	CGQ
A88					

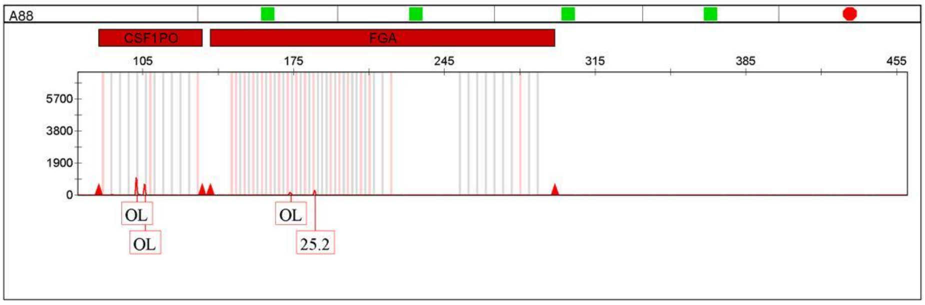

Sample Name	SOS	SQ	SSPK	MIX	CGQ
B13					

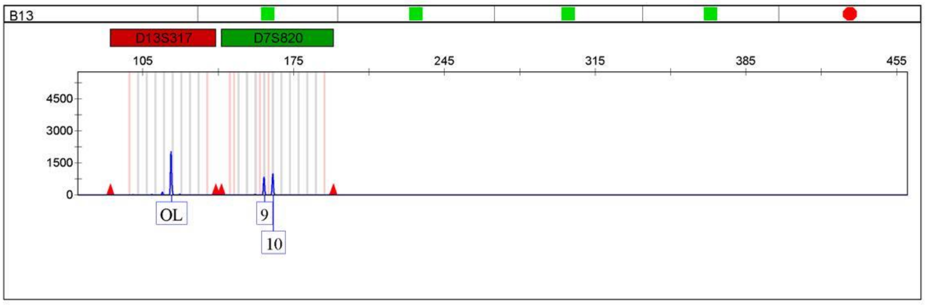

Sample Name	SOS	SQ	SSPK	MIX	CGQ
B13					

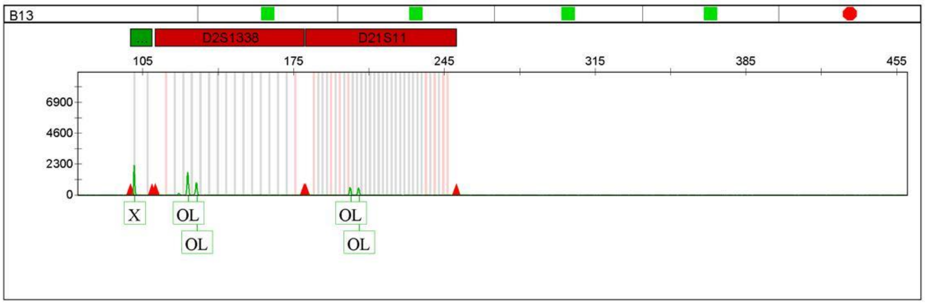

Sample Name	SOS	SQ	SSPK	MIX	CGQ
B13					

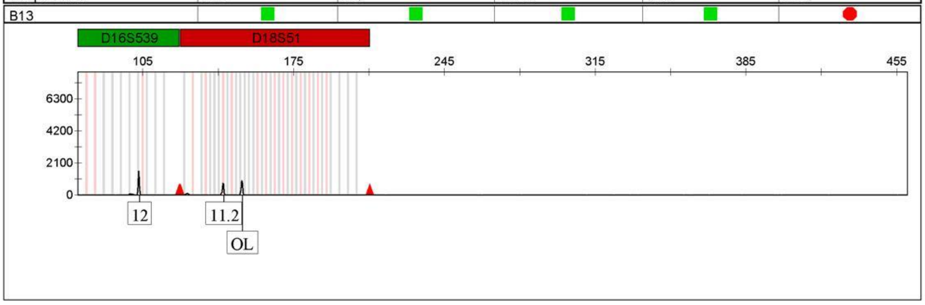

Sample Name	SOS	SQ	SSPK	MIX	CGQ
B13					

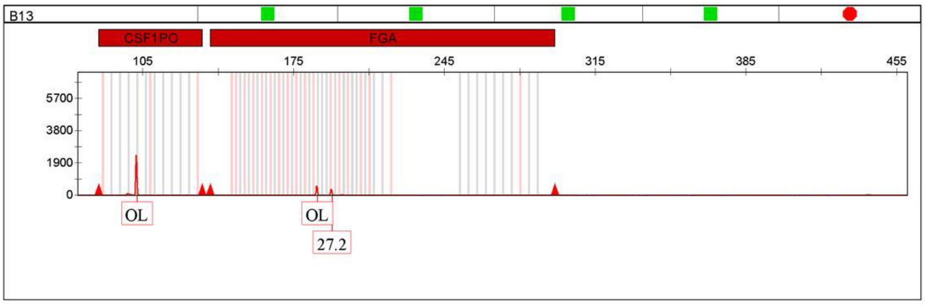

Sample Name	SOS	SQ	SSPK	MIX	CGQ
B34					

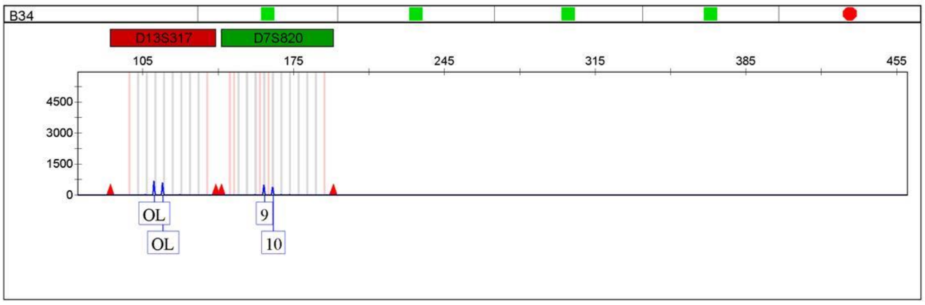

Sample Name	SOS	SQ	SSPK	MIX	CGQ
B34					

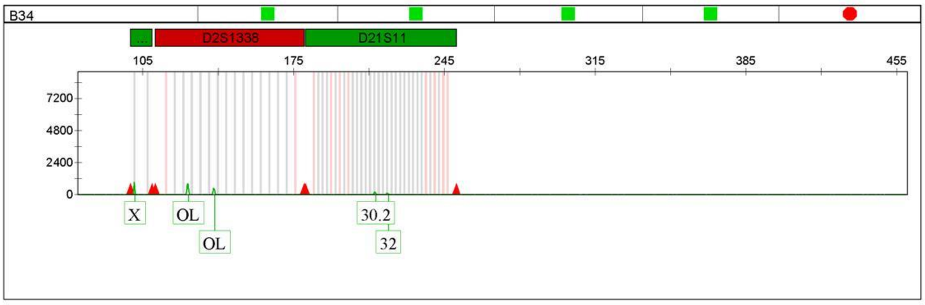

Sample Name	SOS	SQ	SSPK	MIX	CGQ
B34					

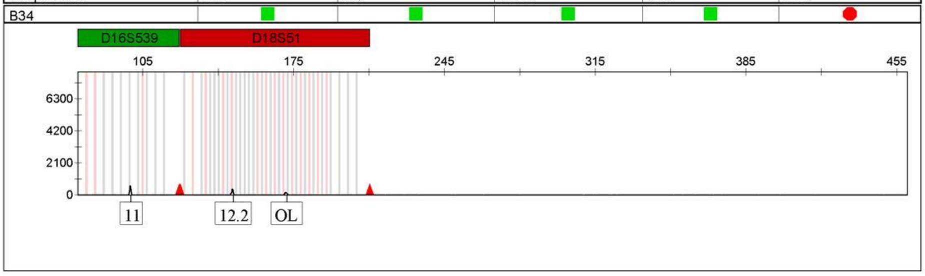

B34					

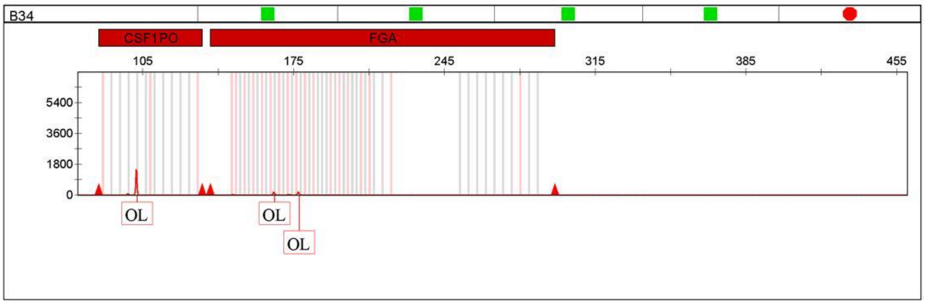

1					

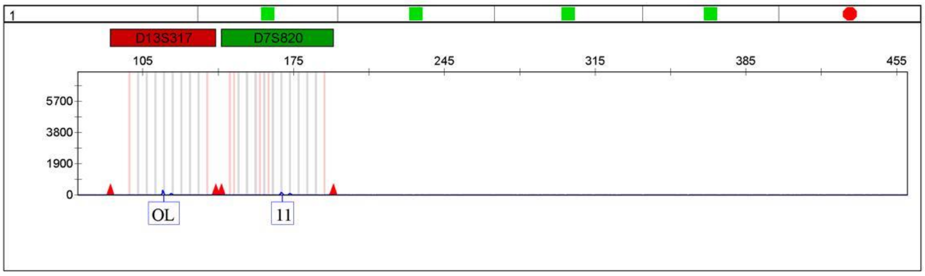

1					

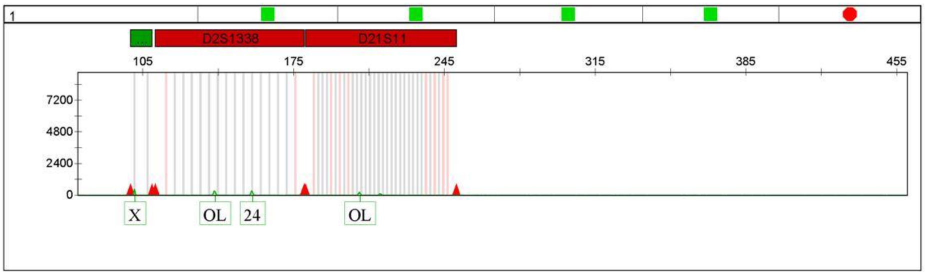

Sample Name	SOS	SQ	SSPK	MIX	CGQ
1					

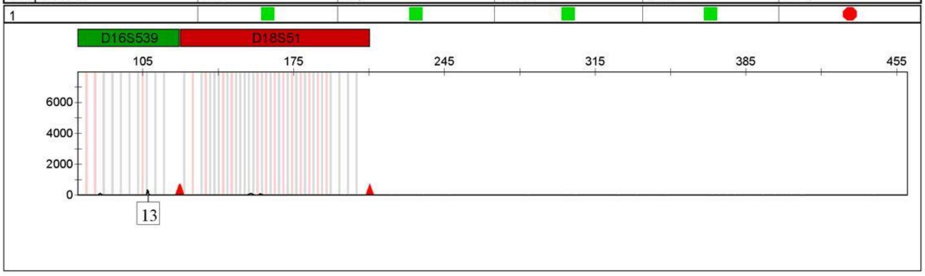

Sample Name	SOS	SQ	SSPK	MIX	CGQ
1					

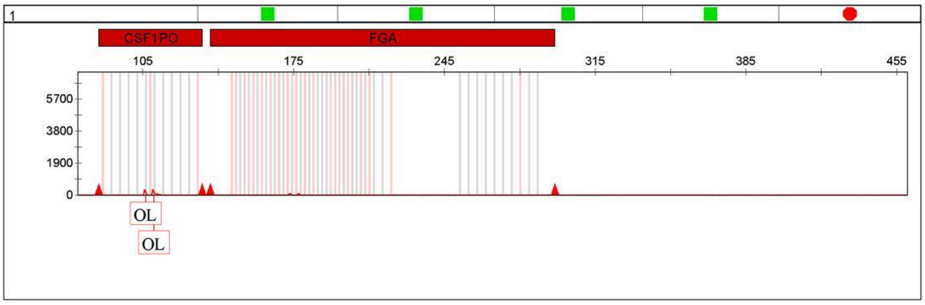

Sample Name	SOS	SQ	SSPK	MIX	CGQ
9					

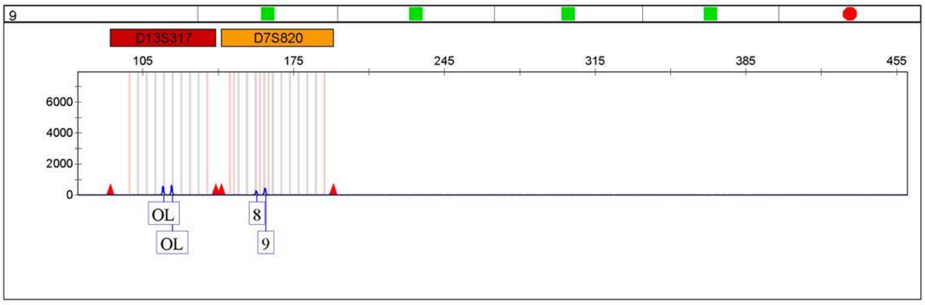

Sample Name	SOS	SQ	SSPK	MIX	CGQ
9					

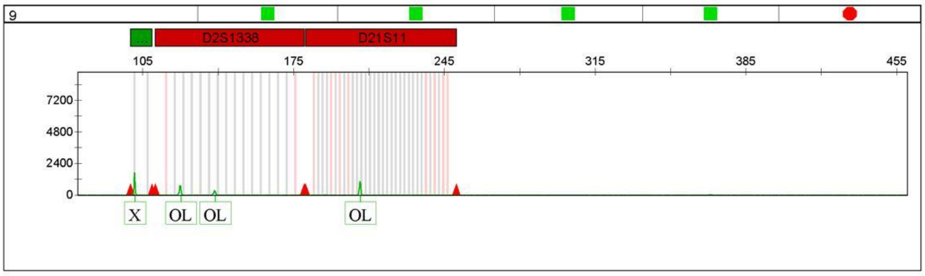

Sample Name	SOS	SQ	SSPK	MIX	CGQ
9					

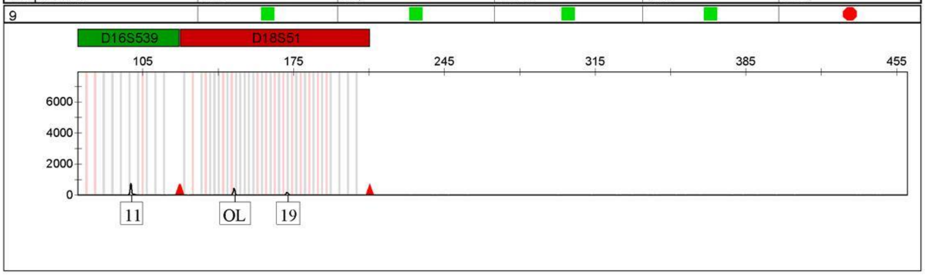

9

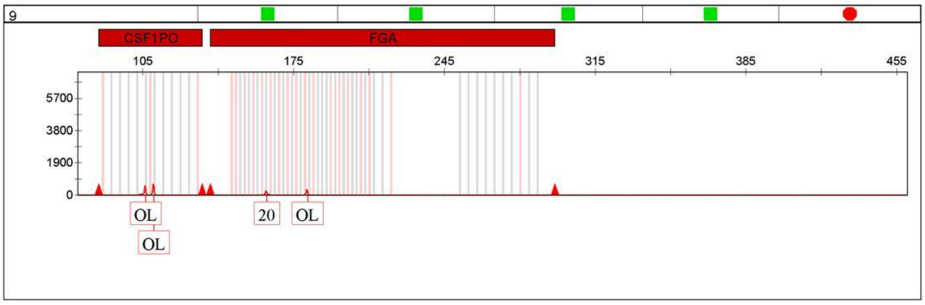

17

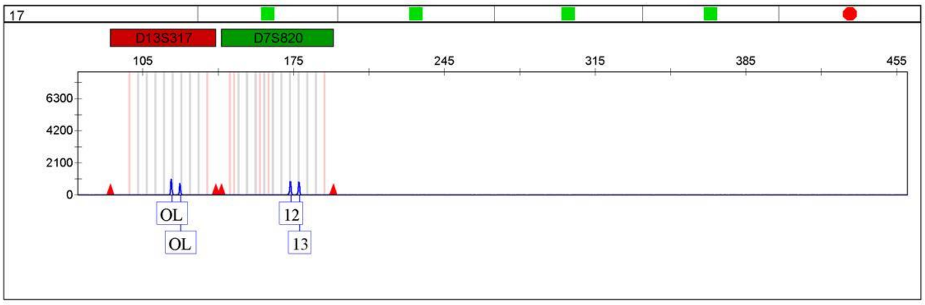

17

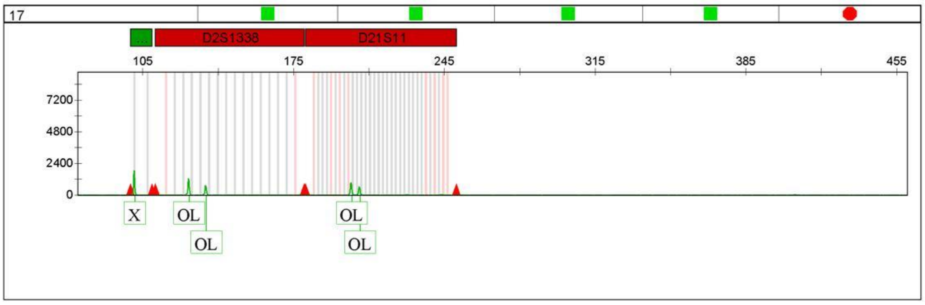

Sample Name	SOS	SQ	SSPK	MIX	CGQ
17					

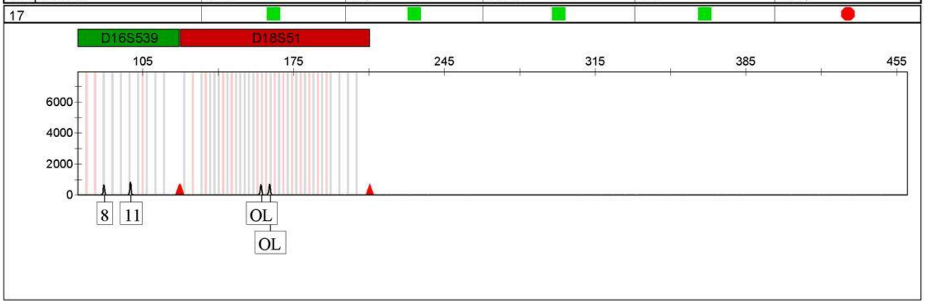

17					

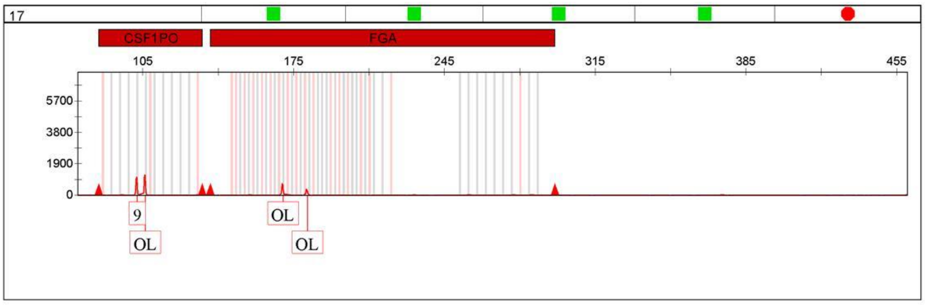

25					

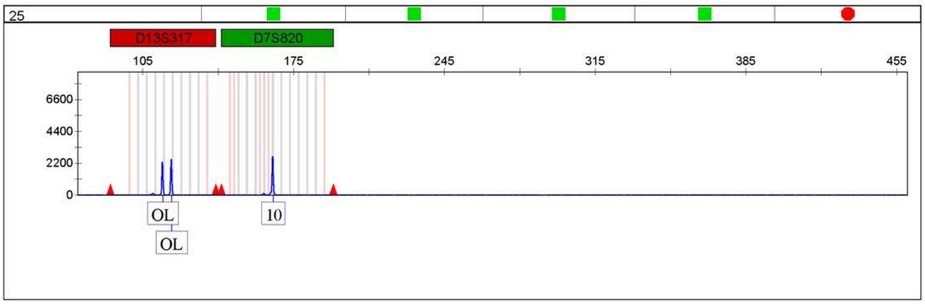

25					

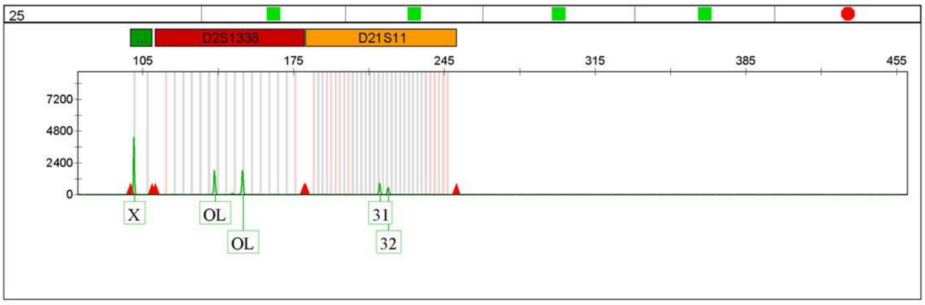

Sample Name	SOS	SQ	SSPK	MIX	CGQ
25					

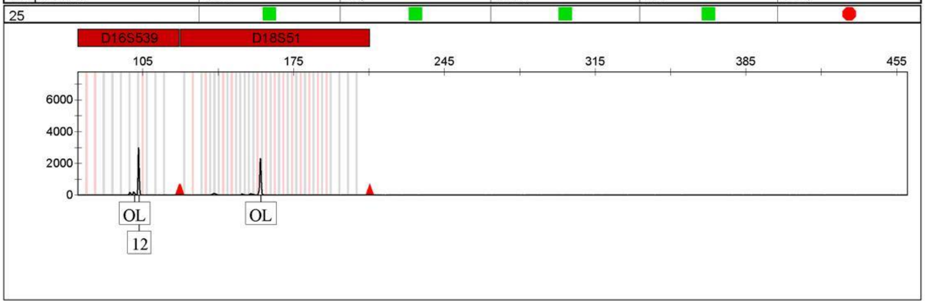

25					

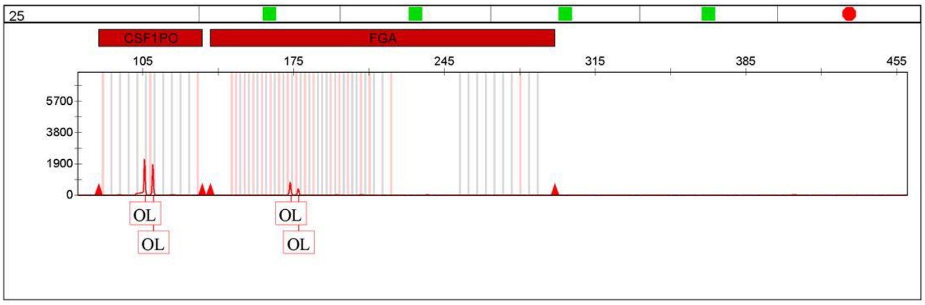

6B					

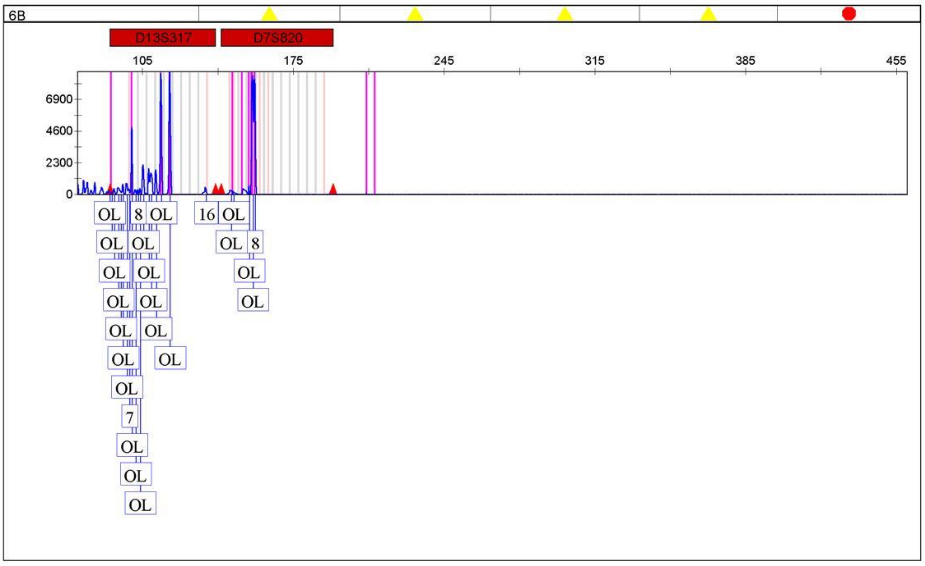

Sample Name	SOS	SQ	SSPK	MIX	CGQ
6B					

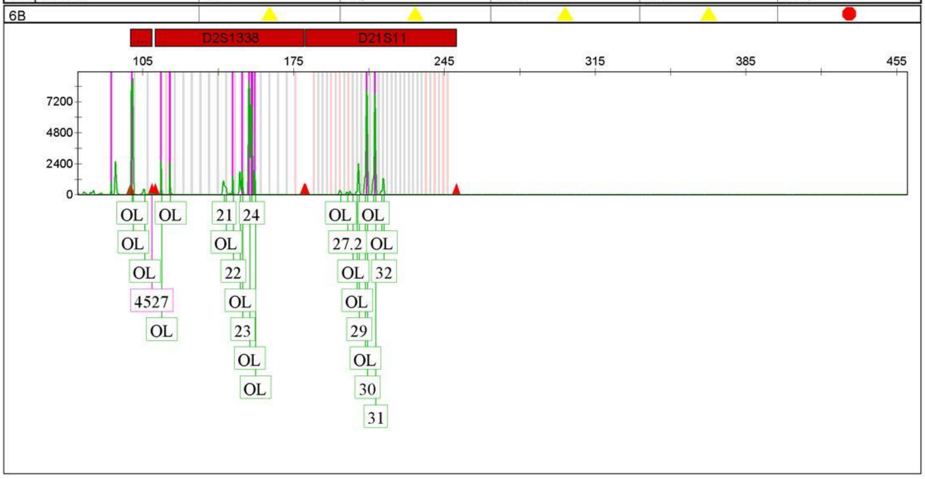

6B					

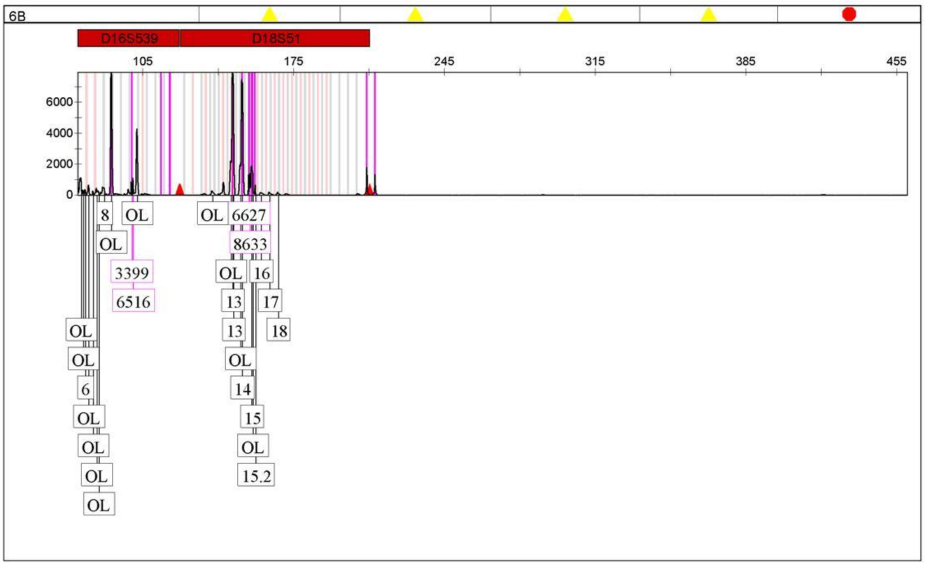

Sample Name	SOS	SQ	SSPK	MIX	CGQ
6B					

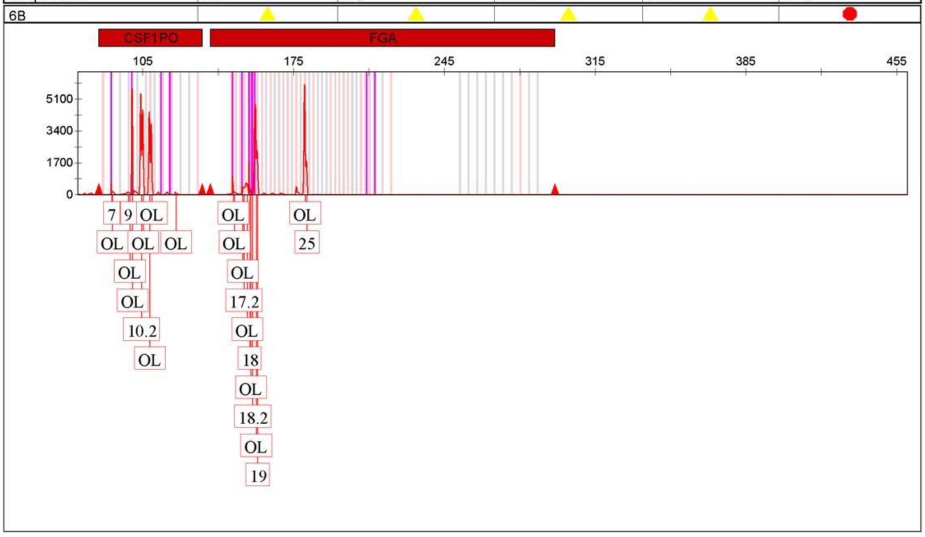

A33					

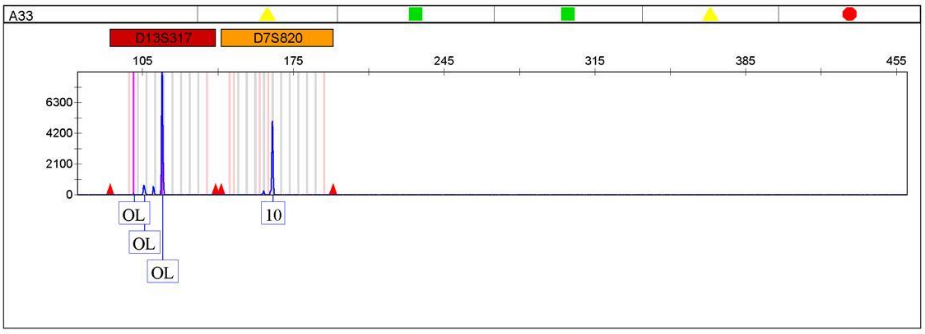

A33					

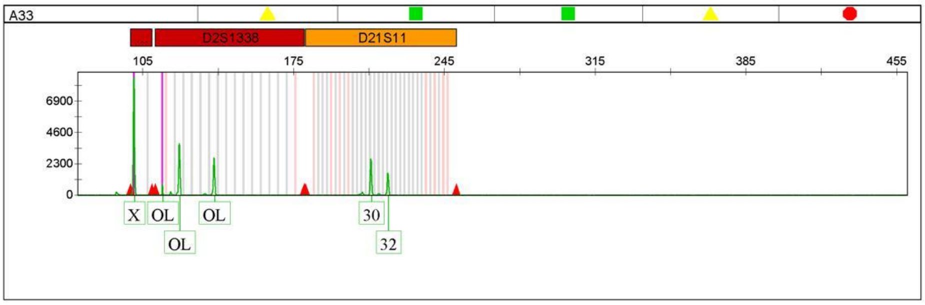

Sample Name	SOS	SQ	SSPK	MIX	CGQ
A33					

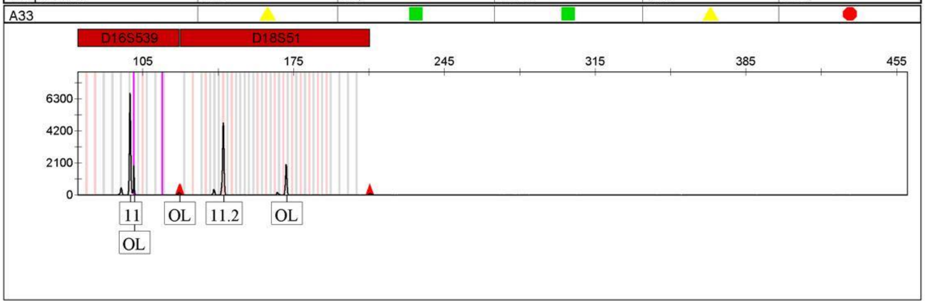

A33					

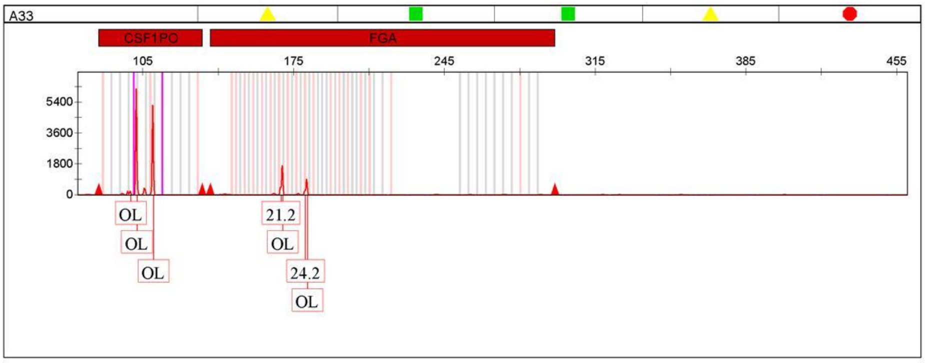

A79					

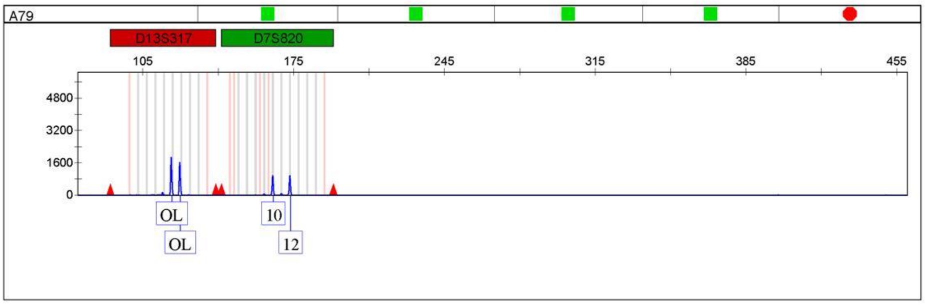

Sample Name	SOS	SQ	SSPK	MIX	CGQ
A79					

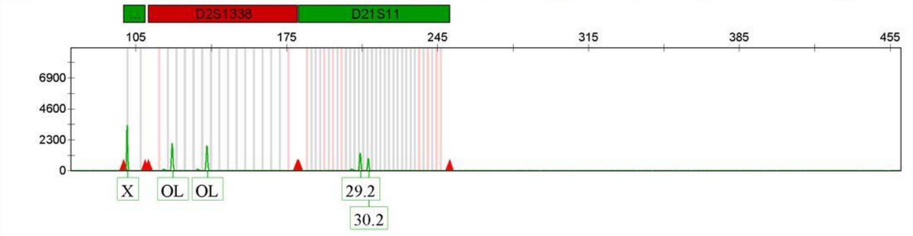

A79					

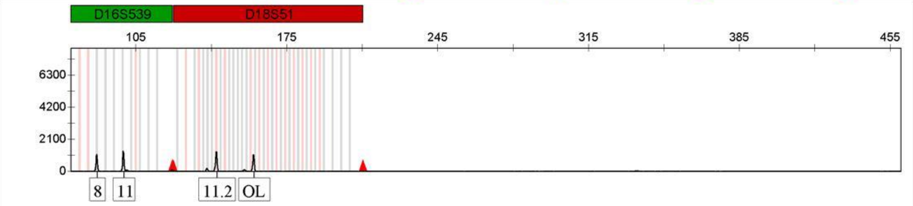

A79					

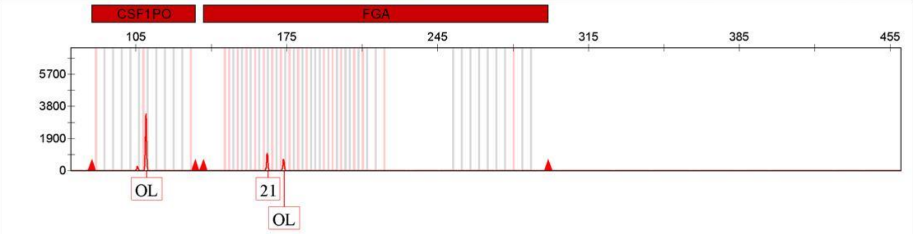

A94					

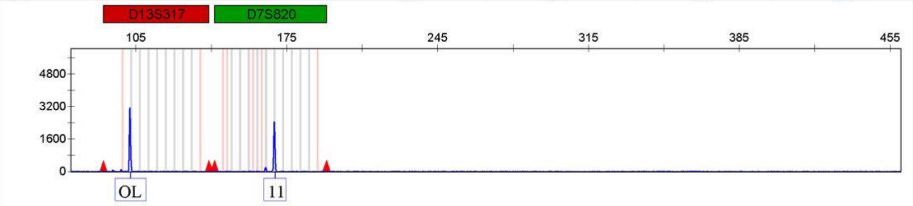

Sample Name	SOS	SQ	SSPK	MIX	CGQ
A94					

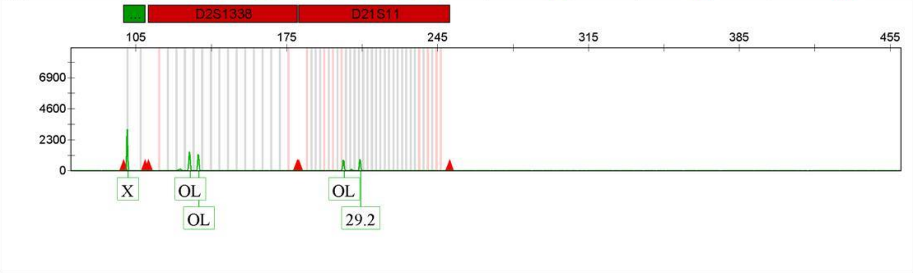

A94					

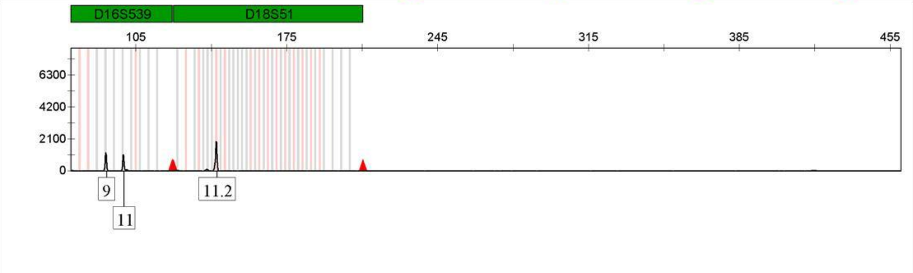

A94					

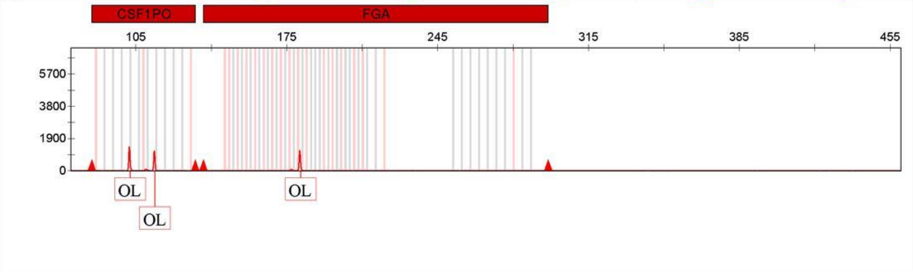

A25					

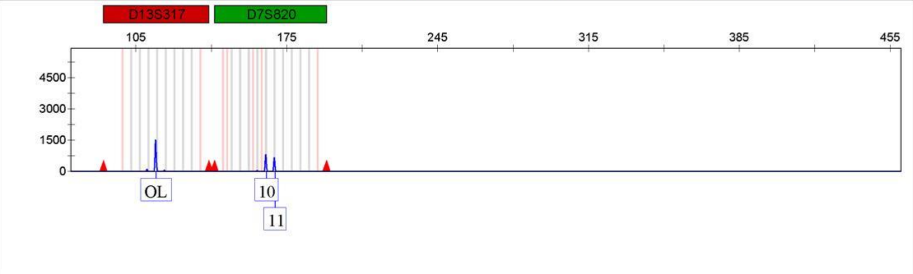

Sample Name	SOS	SQ	SSPK	MIX	CGQ
A25					

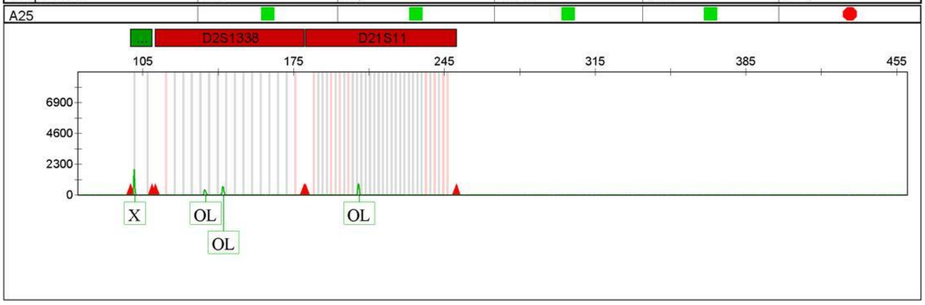

A25					

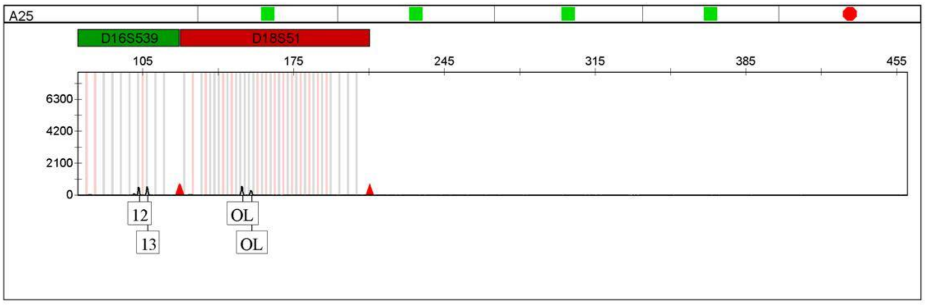

A25					

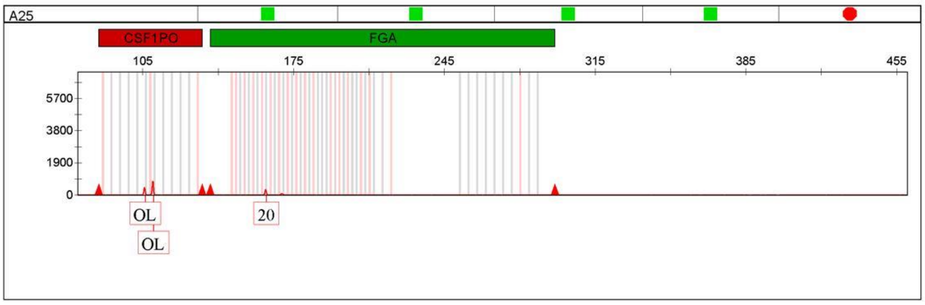

B38					

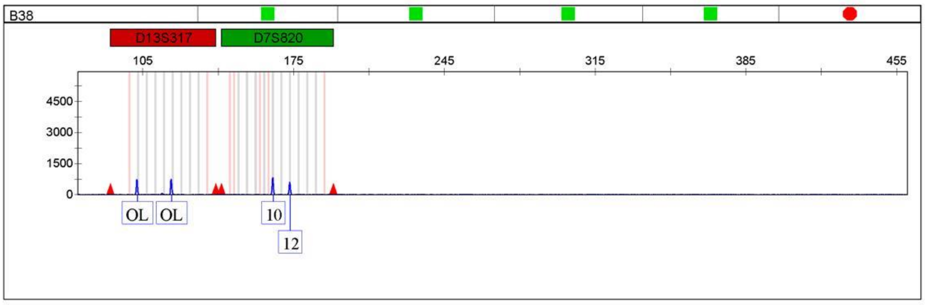

Sample Name	SOS	SQ	SSPK	MIX	CGQ
B38					

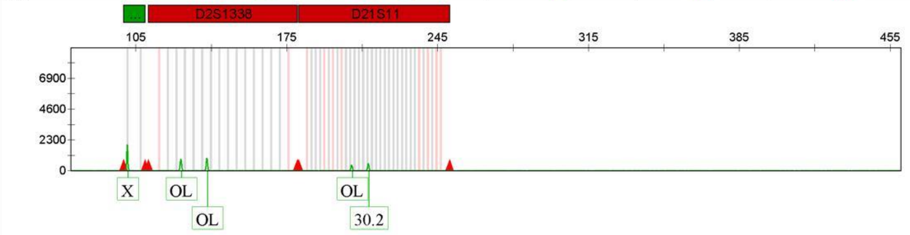

B38					

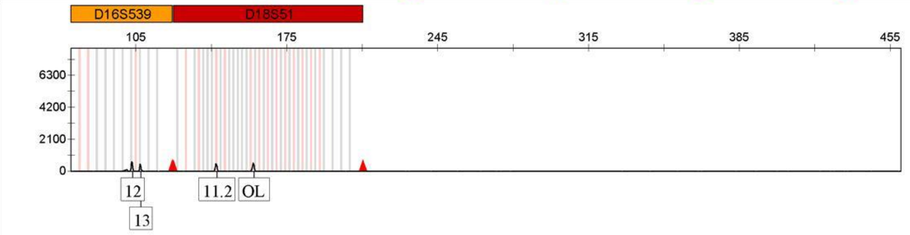

B38					

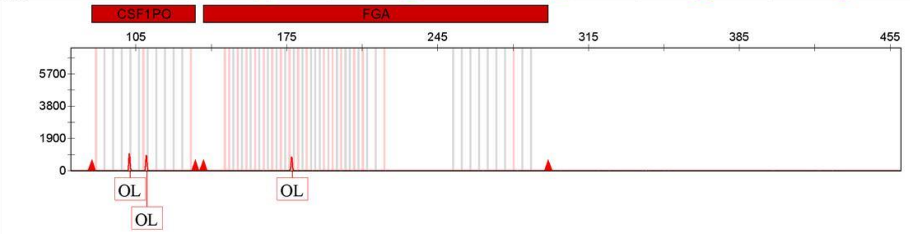

5					

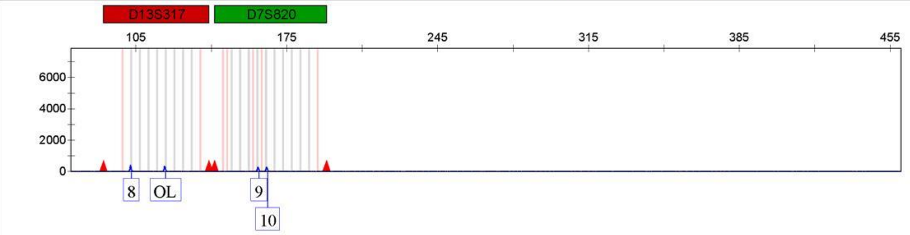

Sample Name	SOS	SQ	SSPK	MIX	CGQ
5					

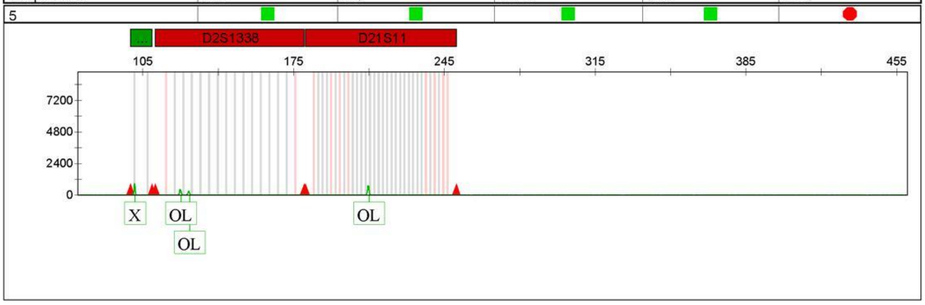

5					

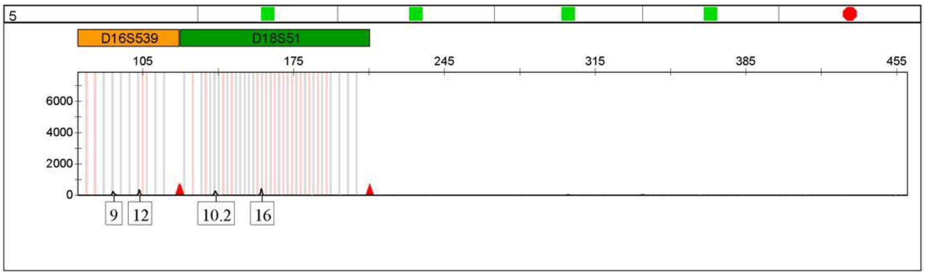

5					

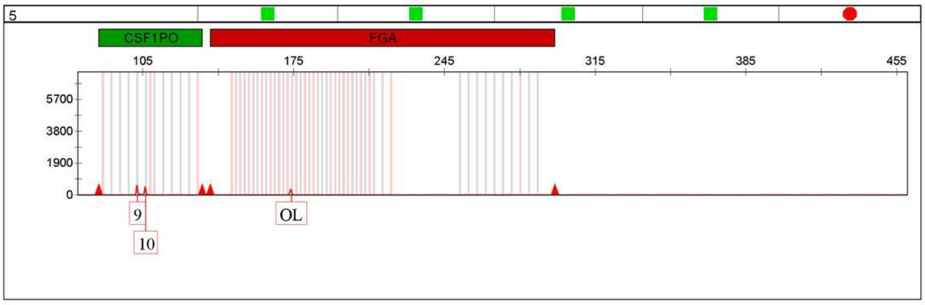

13					

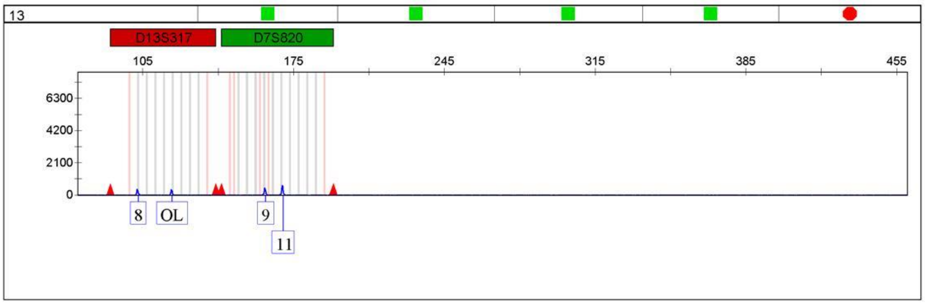

Sample Name	SOS	SQ	SSPK	MIX	CGQ
13					

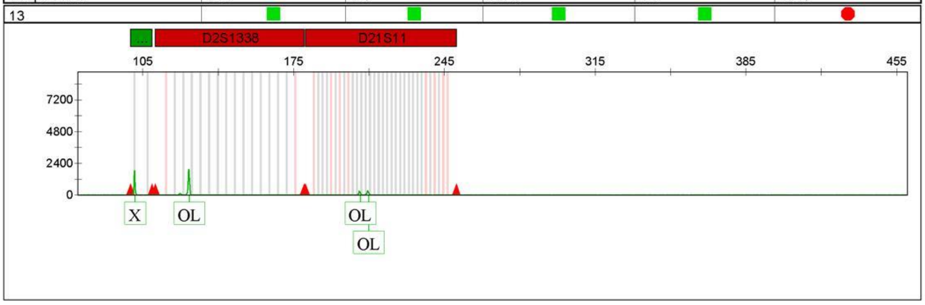

Sample Name	SOS	SQ	SSPK	MIX	CGQ
13					

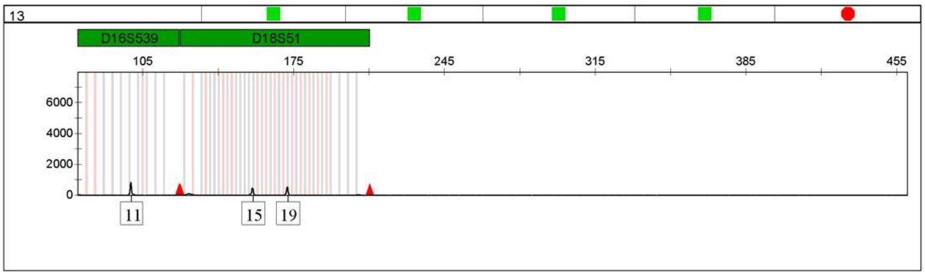

Sample Name	SOS	SQ	SSPK	MIX	CGQ
13					

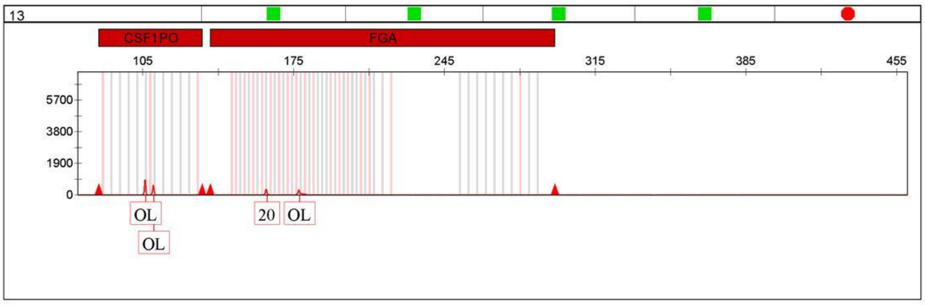

Sample Name	SOS	SQ	SSPK	MIX	CGQ
21					

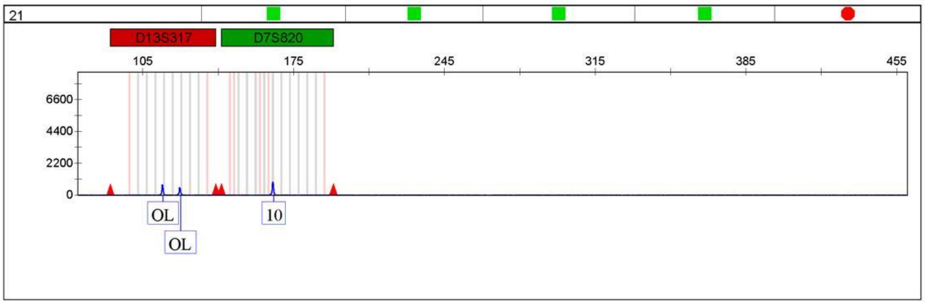

Sample Name	SOS	SQ	SSPK	MIX	CGQ
21					

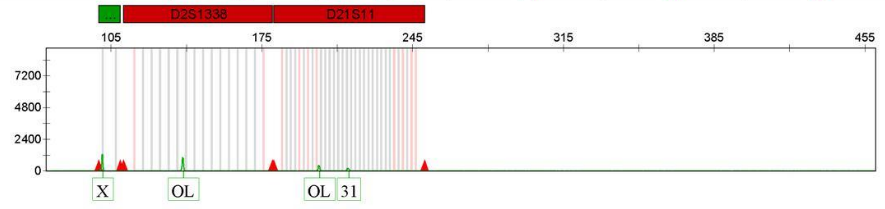

Sample Name	SOS	SQ	SSPK	MIX	CGQ
21					

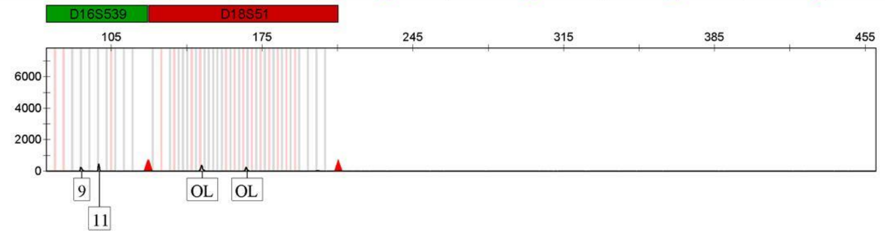

Sample Name	SOS	SQ	SSPK	MIX	CGQ
21					

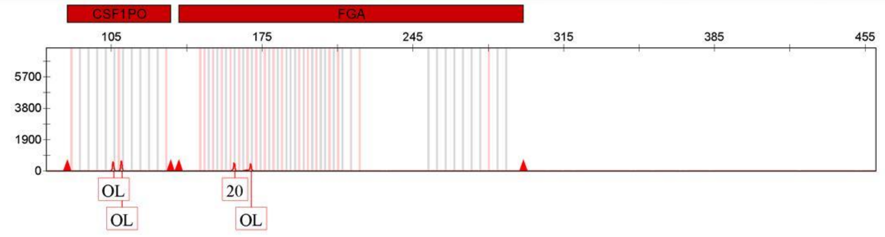

Sample Name	SOS	SQ	SSPK	MIX	CGQ
30					

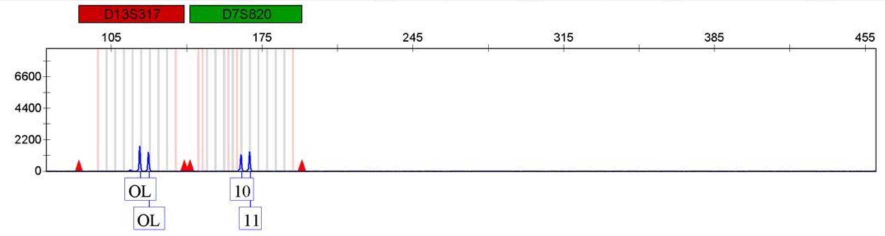

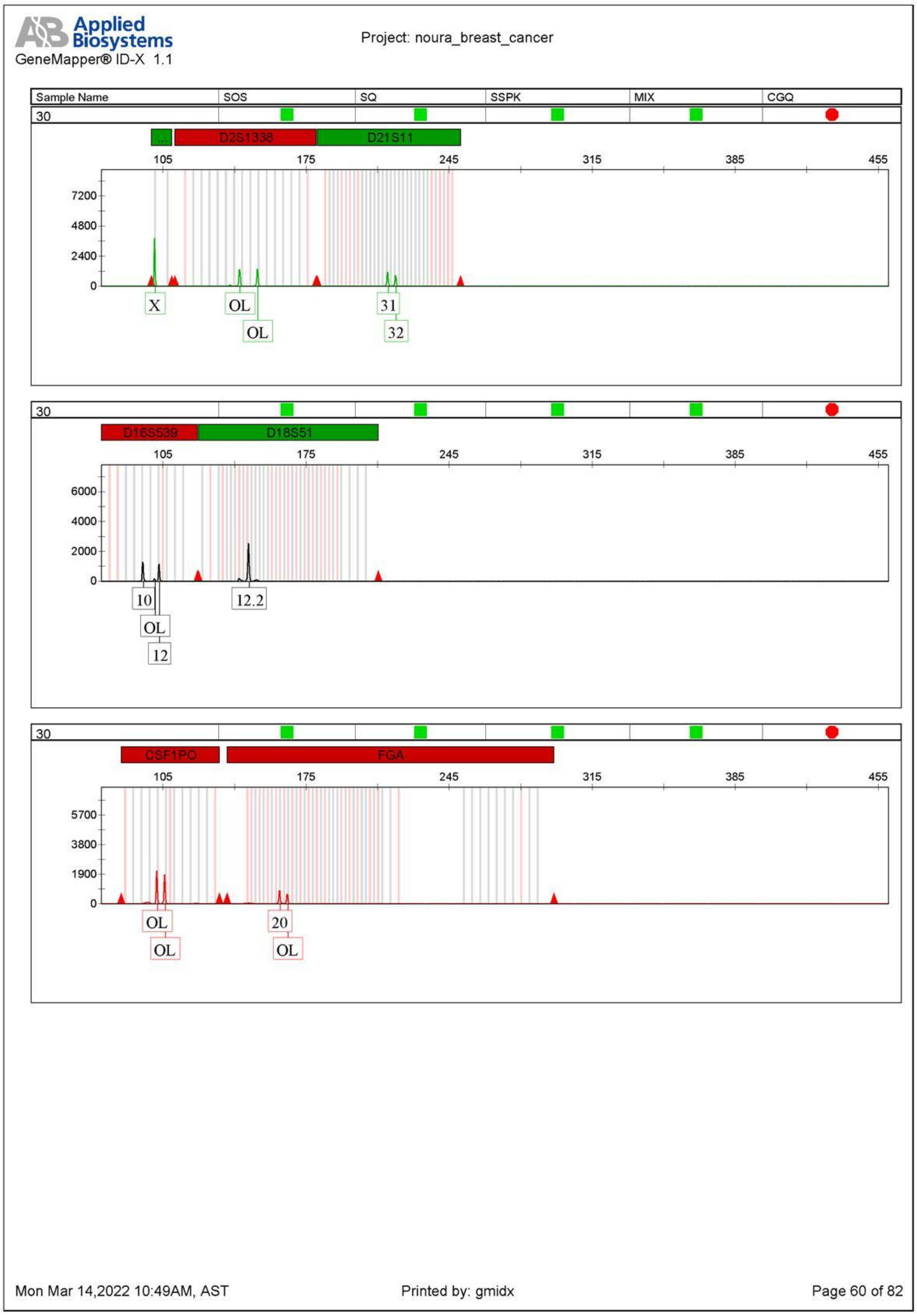

Figure S1. Electropherogram generated from short tandem repeat profiling of cfDNA and genomic DNA extracted from BC patients.
