# Supplementary material for: Utility of Circulating Cell-Free DNA in Assessing Microsatellite Instability and Loss of Heterozygosity in Breast Cancer Using Human Identification Approach
Source: Genes (Basel). 2022 Mar 25;13(4):590. doi: 10.3390/genes13040590 (PMC9027523; doi:10.3390/genes13040590)
Supplement: Supplementary file 1 [file genes-13-00590-s001.zip › Supplementary Table S1.pdf]

Table S1. STR profiling results of Cf-DNA and genomic DNA extracted from healthy controls' samples.

Sample ID	D13S317	D7S820	D2S1338	D21S11	D16S539	D18S51	CSF1P0	FGA	Alleles (%)	Genetic alterations
C1	11,12	11,12	20,24	29,31	11,13	14.2,16	11,12	23,24	12/16 (75%)	-LOH in D13S317: deletion of allele (12)<br>-LOH in D7S820: deletion of allele (12)<br>-Allelic dropout in D18S51 and FGA
Cp1	11,11	11,11	20,24	29,31	11,13	DO	11,12	DO		
C2	9,13	9,10	17,17	29,29	10,11	13.2,17	12,12	20,24	16/16 (100%)	
Cp2	9,13	9,10	17,17	29,29	10,11	13.2,17	12,12	20,24		
C3	12,12	8,12	19,20	29.2,29.2	9,11	13,16	11,11	24,25	16/16 (100%)	
Cp3	12,12	8,12	19,20	29.2,29.2	9,11	13,16	11,11	24,25		
C4	11,13	9,12	16,17	28.2,28.2	11,12	11,15	12,12	19,21	16/16 (100%)	
Cp4	11,13	9,12	16,17	28.2,28.2	11,12	11,15	12,12	19,21		
C5	8,12	9,10	16,17	29.2,29.2	9,12	11,16	10,11	23,23	16/16 (100%)	
Cp5	8,12	9,10	16,17	29.2,29.2	9,12	11,16	10,11	23,23		

C6	8,12	10,11	18,20	29.2,29.2	9,12	13.2,17	12,12	21,23	16/16 (100%)	
Cp6	8,12	10,11	18,20	29.2,29.2	9,12	13.2,17	12,12	21,23		
C7	10,12	10,12	20,25	28.2,29.2	11,11	11,17	11,11	22,24	16/16 (100%)	
Cp7	10,12	10,12	20,25	28.2,29.2	11,11	11,17	11,11	22,24		
C8	11,12	9,11	17,19	29,30	11,11	13,18	10,11	22,25	16/16 (100%)	
Cp8	11,12	9,11	17,19	29,30	11,11	13,18	10,11	22,25		
C9	11,12	8,9	16,20	28.2,28.2	11,11	13,19	11,12	20,25	16/16 (100%)	
Cp9	11,12	8,9	16,20	28.2,28.2	11,11	13,19	11,12	20,25		
C10	11,12	8,10	19,22	28,32	12,12	13,13.2	10,12	23,26	16/16 (100%)	
Cp10	11,12	8,10	9,22	28,32	12,12	13,13.2	10,12	23,26		
C11	11,11	10,10	20,21	28,29.2	11,12	13,21	11,12	23,24	16/16 (100%)	
Cp11	11,11	10,10	20,21	28.2,29.2	11,12	13,21	11,12	23,24		
C12	11,12	8,9	21,26	28,28	11,11	13.2,14.2	11,11	20,26	16/16 (100%)	
Cp12	11,12	8,9	21,26	28,28	11,11	13.2,14.2	11,11	20,26		
C13	8,12	9,11	17,17	28.2,29.2	11,11	14.2,19	11,12	20,24	16/16 (100%)	

Cp13	8,12	9,11	17,17	28.2,29.2	11,11	14.2,19	11,12	20,24		
C14	9,12	10,11	16,19	29.2,31	9,12	12,19	11,11	19,22	12/16 (75%)	Allelic dropout in D21S11 and FGA
Cp14	9,12	10,11	16,19	DO	9,12	12,19	11,11	DO		
C15	12,14	8,10	20,23	29,32.2	12,13	12,20	10,10	19,23	16/16 (100%)	
Cp15	12,14	8,10	20,23	29,32.2	12,13	12,20	10,10	19,23		
C16	8,14	8,10	17,23	28,29.2	9,11	15,17	11,12	19,25	16/16 (100%)	
Cp16	8,14	8,10	17,23	28,29.2	9,11	15,17	11,12	19,25		
C17	12,13	12,13	17,19	28,29	8,11	16,17	9,11	22,25	16/16 (100%)	
Cp17	12,13	12,13	17,19	28,29	8,11	16,17	9,11	22,25		
C18	9,11	9,11	19,21	28,29	8,12	13,14	10,11	23,24	16/16 (100%)	
Cp18	9,11	9,11	19,21	28,29	8,12	13,14	10,11	23,24		
C19	8,13	10,11	18,25	28,29	12,13	15,17	10,12	22,23	16/16 (100%)	
Cp19	8,13	10,11	18,25	28,29	12,13	15,17	10,12	22,23		
C20	12,12	10,11	20,22	29,32.2	9,11	12,17	11,11	20,21	16/16 (100%)	
Cp20	12,12	10,11	20,22	29,32.2	9,11	12,17	11,11	20,21		

C21	11,13	10,10	20,20	28,31.2	9,11	13,18	11,12	20,22	16/16 (100%)	
Cp21	11,13	10,10	20,20	28,31.2	9,11	13,18	11,12	20,22		
C22	11,13	8,11	16,20	30,31.2	11,11	12,12	10,12	24,24	16/16 (100%)	
Cp22	11,13	8,11	16,20	30,31.2	11,11	12,12	10,12	24,24		
C23	9,11	8,9	17,23	28,30	11,12	13,14	10,11	20,22	16/16 (100%)	- LOH in D18S51: deletion of allele (13)<br>- LOH in FGA: deletion of allele (20)
Cp23	9,11	8,9	17,23	28,30	11,12	14,14	10,11	22,22		
C24	11,13	9,10	20,21	29,29.2	9,11	14,18	10,11	21,25	16/16 (100%)	
Cp24	11,13	9,10	20,21	29,29.2	9,11	14,18	10,11	21,25		
C25	11,12	10,10	20,23	31.2,32.2	11,12	16,16	11,12	23,24	16/16 (100%)	
Cp25	11,12	10,10	20,23	31.2,32.2	11,12	16,16	11,12	23,24		
C26	11,12	10,12	19,19	29,33.2	8,11	12,14	11,11	22,23	16/16 (100%)	
Cp26	11,12	10,12	19,19	29,33.2	8,11	12,14	11,11	22,23		
C27	11,11	8,8	21,24	29,29	11,13	16,20	12,12	22,23	16/16 (100%)	

Cp27	11,11	8,8	21,24	29,29	11,13	16,20	12,12	22,23		
C28	12,12	10,11	17,17	29,32.2	11,13	13,16	10,12	24,25	16/16 (100%)	
Cp28	12,12	10,11	17,17	29,32.2	11,13	13,16	10,12	24,25		
C29	8,12	8,8	19,20	29,32.2	10,10	13,14	11,12	23,25	16/16 (100%)	
Cp29	8,12	8,8	19,20	29,32.2	10,10	13,14	11,12	23,25		
C30	12,13	10,11	21,23	31.2,32.2	10,12	13,13	10,11	20,21	16/16 (100%)	
Cp30	12,13	10,11	21,23	31.2,32.2	10,12	13,13	10,11	20,21		
C31	12,13	10,10	20,23	29,33	12,13	16,16	10,11	21,27	16/16 (100%)	
Cp31	12,13	10,10	20,23	29,33	12,13	16,16	10,11	21,27		
C32	9,11	8,10	17,26	29,31.2	9,12	14,18	10,11	21,25	16/16 (100%)	
Cp32	9,11	8,10	17,26	29,31.2	9,12	14,18	10,11	21,25		
C33	11,12	11,12	16,18	29,32.2	11,14	15,16	10,11	24,29	16/16 (100%)	
Cp33	11,12	11,12	16,18	29,32.2	11,14	15,16	10,11	24,29		
C34	11,12	10,10	20,21	30,31.2	8,11	16,19	10,11	23,25	16/16 (100%)	
Cp34	11,12	10,10	20,21	30,31.2	8,11	16,19	10,11	23,25		

C35	10,12	10,11	25,25	28,29	8,8	13,17	10,10	21,24	16/16 (100%)	
Cp35	10,12	10,11	25,25	28,29	8,8	13,17	10,10	21,24		
C36	12,13	10,11	18,22	28,30	11,13	12,18	11,12	27,28	16/16 (100%)	
Cp36	12,13	10,11	18,22	28,30	11,13	12,18	11,12	27,28		
C37	8,12	8,12	17,23	32.2,32.2	9,12	12,13	10,13	23,24	16/16 (100%)	
Cp37	8,12	8,12	17,23	32.2,32.2	9,12	12,13	10,13	23,24		
C38	8,11	11,12	19,20	28,31.2	11,11	14,18	11,11	20,24	16/16 (100%)	
Cp38	8,11	11,12	19,20	28,31.2	11,11	14,18	11,11	20,24		
C39	12,13	10,12	20,21	30,31	9,12	14,19	10,10	25,25	16/16 (100%)	- LOH in D7S820: deletion of allele (12) -LOH in D18S51: Deletion of allele (19)
Cp39	12,13	10,10	20,21	30,31	9,12	14,14	10,10	25,25		
C40	8,13	8,8	20,23	31.2,32.2	11,11	12,16	10,13	21,23	16/16 (100%)	
Cp40	8,13	8,8	20,23	31.2,32.2	11,11	12,16	10,13	21,23		

C, genomic DNA STR profile; **Cp**, Cf-DNA STR profile; **DO**, allelic dropout; **LOH**, loss of heterozygosity; **MSI**, microsatellite instability.
