# Supplementary material for: Utility of Circulating Cell-Free DNA in Assessing Microsatellite Instability and Loss of Heterozygosity in Breast Cancer Using Human Identification Approach
Source: Genes (Basel). 2022 Mar 25;13(4):590. doi: 10.3390/genes13040590 (PMC9027523; doi:10.3390/genes13040590)
Supplement: Supplementary file 1 [file genes-13-00590-s001.zip › Supplementary Table S2.pdf]

Table S2. STR profiling results of Cf-DNA and genomic DNA of BC patients.

Sample ID	D13S317	D7S820	D2S1338	D21S11	D16S539	D18S51	CSF1P0	FGA	Alleles (%)	Genetic alterations
p1	12,14	10,10	22,24	29.2,30	11,11	12,14	11,11	23,25	2/16 (12.5%)	LOH: Allelic dropout except for **D7S820** locus
Pp1	DO	10,10	DO	DO	DO	DO	DO	DO		
p2	11,11	9,9	20,20	31,32.2	11,11	14,19	11,11	23,24	16/16 (100%)	LOH: Loss of allele (24) for locus **FGA**
Pp2	11,11	9,9	20,20	31,32.2	11,11	14,19	11,11	23,23		
p3	11,13	10,10	19,20	29.2,32.2	12,13	13,15	10,12	18,21	16/16 (100%)	
Pp3	11,13	10,10	19,20	29.2,32.2	12,13	13,15	10,12	18,21		
p4	11,11	9,11	19,20	28.2,31	11,13	14.2,17	10,12	20,22	8/16 (50%)	- **LOH in D7S820:** Loss of allele (11)<br>- **D21S11:** allele dropout<br>- **LOH** in **D16S539:** loss of allele (13)<br>- **D18S51:** allele dropout
Pp4	11,11	9,9	19,20	DO	11,11	DO	DO	DO		

										- **CSF1P0:** allele dropout<br>- **FGA:** allelic dropout
p5	11,12	8,9	19,24	28,29.2	9,12	14.2,16	9,12	20,23	0/16 (0%)	Complete allelic Dropout
Pp5	DO	DO	DO	DO	DO	DO	DO	DO		
p6	11,11	10,10	16,20	30,32	11,11	12,19	10,12	22,25	16/16 (100%)	
Pp6	11,11	10,10	16,20	30,32	11,11	12,19	10,12	22,25		
p7	8,12	10,12	17,20	28.2,30.2	12,13	12,16	10,12	24,24	16/16 (100%)	
Pp7	8,12	10,12	17,20	28.2,30.2	12,13	12,16	10,12	24,24		
p8	10,12	12,13	17,20	28.2,29.2	11,13	16,17	10,12	22,25	16/16 (100%)	- **D13S317:** Deletion of allele (12) and insertion of allele (11).<br>- **D16S539:** Deletion of alleles (11,13) and insertion of alleles (9,12)
Pp8	10,11	12,13	17,20	28.2,29.2	9,12	16,17	10,12	22,25		

p9	9,12	10,12	24,25	30,30	11,13	12,18	10,12	24,24	14/16 (87.5%)	- **D7S820: allelic dropout**<br>- LOH in **D18S51:**<br>- Deletion of allele (12)
Pp9	9,12	DO	24,25	30,30	11,13	18,18	10,12	24,24		
p10	10,11	11,12	20,25	28,28	8,11	12,13	9,12	22,26	16/16 (100%)	LOH in **CSF1P0: deletion of allele** (9)
Pp10	10,11	11,12	20,25	28,28	8,11	12,13	12,12	22,26		
p11	8,13	10,10	17,22,26	29.2,33	11,11	14,16	10,12	19,20	16/17 (94.12%)	- LOH in **D13S317:** Deletion of allele (8)<br>- Deletion of allele (26) (triallelic pattern)
Pp11	13,13	10,10	17,22	29.2,33	11,11	14,16	10,12	19,20		
p12	12,14	9,10	16,20	28,28	10,13	15,16	11,13	23,23	16/16 (100%)	
Pp12	12,14	9,10	16,20	28,28	10,13	15,16	11,13	23,23		
p13	8,11	11,11	19,21	28,29	11,13	16,19	9,12	25,26	16/16 (100%)	LOH in **CSF1P0: deletion of allele (9)**
Pp13	8,11	11,11	19,21	28,29	11,13	16,19	12,12	25,26		
p14	12,13	10,12	16,20	29.2,30.2	8,11	12,16	12,12	21,23	16/16 (100%)	

Pp14	12,13	10,12	16,20	29.2,30.2	8,11	12,16	12,12	21,23		
p15	12,13	7,9	20,20	28.2,31.2	11,13	14,16	9,10,11	18,22	17/17 (100%)	- MSI in **CSF1P0**<br>- **FGA:** Deletion of allele (22) and insertion of (21)
Pp15	12,13	7,9	20,20	28.2,31.2	11,13	14,16	9,10,11	18,21		
p16	12,12	8,10	19,25	28,31.2	11,11	14.2,16	10,13	19,25	16/16 (100%)	- **D13S317** MSI**: d**eletion of genotype (12,12) and insertion of (11,11)<br>- **D7S820** MSI**: d**eletion of (8,10) and insertion of (9,11)<br>- **D18S51:** Deletion of allele (16) and insertion of (17)<br>- LOH in **FGA:** Deletion of allele (19)
Pp16	11,11	9,11	19,25	28,31.2	11,11	14.2,17	10,13	25,25		
p17	9,11	10,11	17,17	29.2,29.2	11,13	14,14	11,11	23,24	16/16 (100%)	
Pp17	9,11	10,11	17,17	29.2,29.2	11,13	14,14	11,11	23,24		

p18	11,12	9,10	16,24	28,28.2	9,14	13,19	10,11	23,26	16/16 (100%)	LOH in **D2S1338:** Deletion of allele (24)
Pp18	11,12	9,10	16,16	28,28.2	9,14	13,19	10,11	23,26		
p19	10,10	8,11	21,24	28.2,30.2	8,9	12,14.2	10,11	22,25	16/16 (100%)	
Pp19	10,10	8,11	21,24	28.2,30.2	8,9	12,14.2	10,11	22,25		
p20	11,13	10,12	16,23	28.2,29.2	12,13	13.2,14.2	9,12,13	22,23	16/17 (94.12%)	**CSF1P0:** Deletion of the triallelic pattern by the deletion of allele (9)
Pp20	11,13	10,12	16,23	28.2,29.2	12,13	13.2,14.2	12,13	22,23		
p21	12,14	9,10	16,20	28,28	10,13	15,16	11,13	23,23	16/16 (100%)	
Pp21	12,14	9,10	16,20	28,28	10,13	15,16	11,13	23,23		
p22	8,8	11,11	18,19	28,29.2	9,11	12,12	10,13	25,25	16/16 (100%)	
Pp22	8,8	11,11	18,19	28,29.2	9,11	12,12	10,13	25,25		
p23	9,12	10,10	17,25	28,29	9,9	14,14	10,11	19,25	16/16 (100%)	
Pp23	9,12	10,10	17,25	28,29	9,9	14,14	10,11	19,25		

p24	10,14	10,11	18,24	29.2,29.2	11,13	13,16	11,12	20,26	16/16 (100%)	
Pp24	10,14	10,11	18,24	29.2,29.2	11,13	13,16	11,12	20,26		
p25	12,12	9,10	17,18	28,28.2	12,12	12,14	10,10	26,28	16/16 (100%)	
Pp25	12,12	9,10	17,18	28,28.2	12,12	12,14	10,10	26,28		
p26	9,12	8,12	20,20	28,31	10,11	18,23	12,12	21,22	16/16 (100%)	
Pp26	9,12	8,12	20,20	28,31	10,11	18,23	12,12	21,22		
p27	8,12	10,10	19,20	28,28.2	11,12	17,17	10,11	22,23	16/16 (100%)	
Pp27	8,12	10,10	19,20	28,28.2	11,12	17,17	10,11	22,23		
p28	11,11	10,12	17,23	27,29.2	11,11	18,18	10,15	21,22	16/16 (100%)	
Pp28	11,11	10,12	17,23	27,29.2	11,11	18,18	10,15	21,22		
p29	12,14	10,12	16,17	28.2,28.2	11,11	12,14.2	9,12	22,24	16/16 (100%)	
Pp29	12,14	10,12	16,17	28.2,28.2	11,11	12,14.2	9,12	22,24		

p30	11,11	10,11	19,21	28.2,28.2	12,13	14,15	11,12	20,22	16/16 (100%)	LOH in FGA: deletion of allele (22)
Pp30	11,11	10,11	19,21	28.2,28.2	12,13	14,15	11,12	20,20		
p31	8,12	10,10	20,25	33,33	11,11	13,16	12,12	22,24	16/16 (100%)	
Pp31	8,12	10,10	20,25	33,33	11,11	13,16	12,12	22,24		
p32	13,14	8,10	17,17	29.2,33	8,12	12,18	10,11	20,23	0/16 (0%)	Complete allelic Dropout
Pp32	DO	DO	DO	DO	DO	DO	DO	DO		
p33	10,11	9,10	17,20	30.2,32	11,11	13,19	10,10	21,24	16/16 (100%)	
Pp33	10,11	9,10	17,20	30.2,32	11,11	13,19	10,10	21,24		
p34	8,11	9,10	19,20	28,29	9,11	13,14	10,12	20,23	16/16 (100%)	
Pp34	8,11	9,10	19,20	28,29	9,11	13,14	10,12	20,23		
p35	12,13	9,13	20,20	28,30	11,12	16,20	10,12	23,25	16/16 (100%)	
Pp35	12,13	9,13	20,20	28,30	11,12	16,20	10,12	23,25		

p36	11,12	12,12	17,20	28,30	9,11	12,13	10,12	22,24	16/16 (100%)	LOH in **D21S11:** Deletion of allele (30)
Pp36	11,12	12,12	17,20	28,28	9,11	12,13	10,12	22,24		
p37	11,13	9,10	19,24	28,30	9,12	16,20	10,11	20,20	16/16 (100%)	
Pp37	11,13	9,10	19,24	28,30	9,12	16,20	10,11	20,20		
p38	10,12	10,12	23,23	28,29.2	13,13	13,16	10,11	21,25	16/16 (100%)	LOH in **D21S11** Deletion of allele (29.2)
Pp38	10,12	10,12	23,23	28,28	13,13	13,16	10,11	21,25		
p39	8,11	12,12	12,24	31,32	12,12	13,15	10,10	23,23	16/16 (100%)	- MSI in **D2S1338:**<br>- Deletion of allele (12) and insertion of (18)
Pp39	8,11	12,12	18,24	31,32	12,12	13,15	10,10	23,23		
p40	12,12	10,11	20,22	28,29.2	11,14	13.2,16	11,12	19,25	12/16 (100%)	Allelic dropout in **D16S539 and FGA**
Pp40	12,12	10,11	20,22	28,29.2	DO	13.2,16	11,12	DO		
p41	12,12	10,11	17,18	30,34	8,10,11	16,17	11,12	22,24	17/17 (100%)	MSI in **D16S539**

Pp41	12,12	10,11	17,18	30,34	8,10,11	16,17	11,12	22,24		

DO, allelic dropout; **LOH**, loss of heterozygosity; **MSI**, microsatellite instability; **P**, genomic DNA STR profile; **Pp**, Cf-DNA STR profile.
